# The Effectiveness of Mobile-Health Technology-Based Health Behaviour Change or Disease Management Interventions for Health Care Consumers: A Systematic Review

**DOI:** 10.1371/journal.pmed.1001362

**Published:** 2013-01-15

**Authors:** Caroline Free, Gemma Phillips, Leandro Galli, Louise Watson, Lambert Felix, Phil Edwards, Vikram Patel, Andy Haines

**Affiliations:** 1 Clinical Trials Unit, London School of Hygiene & Tropical Medicine, London, United Kingdom; 2 Department of Health Services Research and Policy, London School of Hygiene & Tropical Medicine, London, United Kingdom; 3 Warwick University, Coventry, United Kingdom; 4 Department of Population Health, London School of Hygiene & Tropical Medicine, London, United Kingdom; 5 Department of Primary Care and Public Health, Imperial College, London, United Kingdom; London School of Economics, United Kingdom

## Abstract

**Background:**

Mobile technologies could be a powerful media for providing individual level support to health care consumers. We conducted a systematic review to assess the effectiveness of mobile technology interventions delivered to health care consumers.

**Methods and Findings:**

We searched for all controlled trials of mobile technology-based health interventions delivered to health care consumers using MEDLINE, EMBASE, PsycINFO, Global Health, Web of Science, Cochrane Library, UK NHS HTA (Jan 1990–Sept 2010). Two authors extracted data on allocation concealment, allocation sequence, blinding, completeness of follow-up, and measures of effect. We calculated effect estimates and used random effects meta-analysis. We identified 75 trials. Fifty-nine trials investigated the use of mobile technologies to improve disease management and 26 trials investigated their use to change health behaviours. Nearly all trials were conducted in high-income countries. Four trials had a low risk of bias. Two trials of disease management had low risk of bias; in one, antiretroviral (ART) adherence, use of text messages reduced high viral load (>400 copies), with a relative risk (RR) of 0.85 (95% CI 0.72–0.99), but no statistically significant benefit on mortality (RR 0.79 [95% CI 0.47–1.32]). In a second, a PDA based intervention increased scores for perceived self care agency in lung transplant patients. Two trials of health behaviour management had low risk of bias. The pooled effect of text messaging smoking cessation support on biochemically verified smoking cessation was (RR 2.16 [95% CI 1.77–2.62]). Interventions for other conditions showed suggestive benefits in some cases, but the results were not consistent. No evidence of publication bias was demonstrated on visual or statistical examination of the funnel plots for either disease management or health behaviours. To address the limitation of the older search, we also reviewed more recent literature.

**Conclusions:**

Text messaging interventions increased adherence to ART and smoking cessation and should be considered for inclusion in services. Although there is suggestive evidence of benefit in some other areas, high quality adequately powered trials of optimised interventions are required to evaluate effects on objective outcomes.

*Please see later in the article for the Editors' Summary*

## Abstract 2

Caroline Free and colleagues systematically review a fast-moving field, that of the effectiveness of mobile technology interventions delivered to healthcare consumers, and conclude that high-quality, adequately powered trials of optimized interventions are required to evaluate effects on objective outcomes.

## Editors’ Summary

**Background:**

Every year, millions of people die from cardiovascular diseases (diseases of the heart and circulation), chronic obstructive pulmonary disease (a long-term lung disease), lung cancer, HIV infection, and diabetes. These diseases are increasingly important causes of mortality (death) in low- and middle-income countries and are responsible for nearly 40% of deaths in high-income countries. For all these diseases, individuals can adopt healthy behaviors that help prevent disease onset. For example, people can lower their risk of diabetes and cardiovascular disease by maintaining a healthy body weight, and, if they are smokers, they can reduce their risk of lung cancer and cardiovascular disease by giving up cigarettes. In addition, optimal treatment of existing diseases can reduce mortality and morbidity (illness). Thus, in people who are infected with HIV, antiretroviral therapy delays the progression of HIV infection and the onset of AIDS, and in people who have diabetes, good blood sugar control can prevent retinopathy (a type of blindness) and other serious complications of diabetes.

**Why Was This Study Done?:**

Health-care providers need effective ways to encourage "health-care consumers" to make healthy lifestyle choices and to self-manage chronic diseases. The amount of information, encouragement and support that can be conveyed to individuals during face-to-face consultations or through traditional media such as leaflets is limited, but mobile technologies such as mobile phones and portable computers have the potential to transform the delivery of health messages. These increasingly popular technologies—more than two-thirds of the world's population now owns a mobile phone—can be used to deliver health messages to people anywhere and at the most relevant times. For example, smokers trying to quit smoking can be sent regular text messages to sustain their motivation, but can also use text messaging to request extra support when it is needed. But is "mHealth," the provision of health-related services using mobile communication technology, an effective way to deliver health messages to health-care consumers? In this systematic review (a study that uses predefined criteria to identify all the research on a given topic), the researchers assess the effectiveness of mobile technology-based health behavior change interventions and disease management interventions delivered to health-care consumers.

**What Did the Researchers Do and Find?:**

The researchers identified 75 controlled trials (studies that compare the outcomes of people who do and do not receive an intervention) of mobile technology-based health interventions delivered to health-care consumers that met their predefined criteria. Twenty-six trials investigated the use of mobile technologies to change health behaviors, 59 investigated their use in disease management, most were of low quality, and nearly all were undertaken in high-income countries. In one high-quality trial that used text messages to improve adherence to antiretroviral therapy among HIV-positive patients in Kenya, the intervention significantly reduced the patients’ viral load but did not significantly reduce mortality (the observed reduction in deaths may have happened by chance). In two high-quality UK trials, a smoking intervention based on text messaging (txt2stop) more than doubled biochemically verified smoking cessation. Other lower-quality trials indicated that using text messages to encourage physical activity improved diabetes control but had no effect on body weight. Combined diet and physical activity text messaging interventions also had no effect on weight, whereas interventions for other conditions showed suggestive benefits in some but not all cases.

**What Do These Findings Mean?:**

These findings provide mixed evidence for the effectiveness of health intervention delivery to health-care consumers using mobile technologies. Moreover, they highlight the need for additional high-quality controlled trials of this mHealth application, particularly in low- and middle-income countries. Specifically, the demonstration that text messaging interventions increased adherence to antiretroviral therapy in a low-income setting and increased smoking cessation in a high-income setting provides some support for the inclusion of these two interventions in health-care services in similar settings. However, the effects of these two interventions need to be established in other settings and their cost-effectiveness needs to be measured before they are widely implemented. Finally, for other mobile technology–based interventions designed to change health behaviors or to improve self-management of chronic diseases, the results of this systematic review suggest that the interventions need to be optimized before further trials are undertaken to establish their clinical benefits.

**Additional Information:**

Please access these Web sites via the online version of this summary at http://dx.doi.org/10.1371/journal.pmed.1001362.

- A related *PLOS Medicine* Research Article by Free et al. investigates the ability of mHealth technologies to improve health-care service delivery processes
- Wikipedia has a page on mHealth (note: Wikipedia is a free online encyclopedia that anyone can edit; available in several languages)
- mHealth: New horizons for health through mobile technologies is a global survey of mHealth prepared by the World Health Organization’s Global Observatory for eHealth (eHealth is health-care practice supported by electronic processes and communication)
- The mHealth in Low-Resource Settings website, which is maintained by the Netherlands Royal Tropical Institute, provides information on the current use, potential, and limitations of mHealth in low-resource settings
- More information about Txt2stop is available, the UK National Health Service Choices website provides an analysis of the Txt2stop trial and what its results mean, and the UK National Health Service Smokefree website provides a link to a Quit App for the iPhone
- The US Centers for Disease Control and Prevention has launched a text messaging service that delivers regular health tips and alerts to mobile phones

## Introduction

Each year, worldwide, over 22 million people die from diseases such as cardiovascular disease, chronic obstructive pulmonary disease, HIV, lung cancer, and diabetes mellitus [1]. The majority of deaths (83%) occur in low- and middle-income countries; however, these are also important causes of mortality in high-income countries, accounting for about 39% of deaths [1].

Health care consumers adopting healthy behaviours can prevent the onset of many of these leading causes of mortality. Ten years after stopping smoking, the risk of lung cancer is half of that of those continuing smoking and 15 y after stopping smoking the risk of cardiovascular disease is similar to that of those who never smoked [2]. HIV can be prevented by adopting safer sexual practices [3]. In people who are overweight, weight loss can reduce the incidence of diabetes [4].

Optimal treatment of existing diseases can also reduce mortality and morbidity. In patients with existing coronary heart disease long term anti-platelet therapy reduces major vascular events (myocardial infarction, stroke, or vascular mortality) by about a quarter [5], ACE inhibitors reduce cardiovascular mortality by just under a fifth [6], beta blockers reduce mortality by almost a quarter [7], and lipid lowering therapy reduces coronary mortality by about a fifth [8]. Antiretroviral medication delays the progression of HIV infection [9]. Good control of diabetes reduces the onset of retinopathy by half and reduces the onset of nephropathy by two-thirds [10].

Optimal treatment of existing diseases requires the involvement of health care consumers in managing aspects of their disease. Health care consumers decide whether to adhere to prescribed medication and determine when they seek health care [11]. Involving health care consumers in self-management of diseases such as in monitoring their health/disease status and adjusting their medication dosage (e.g., insulin or asthma medications) can improve health outcomes [12]–[14].

An important function of health care services is therefore to encourage and support health care consumers to adopt healthy behaviours and to self-manage chronic diseases. However, the amount of information, encouragement, and support that can be conveyed during consultations, within existing service infrastructures or through other traditional media (such as leaflets), is limited.

Mobile technologies are a means for providing individual level support to health care consumers. Mobile health interventions for health care consumers have been designed to increase healthy behaviour (for example, to increase smoking cessation or activity levels) or improve disease management (for example, by increasing adherence to prescribed medication, improving management of diabetes or asthma, or delivering therapeutic interventions).

Mobile technologies include mobile phones; personal digital assistants (PDAs) and PDA phones (e.g., BlackBerry, Palm Pilot); smartphones (e.g., iPhone); enterprise digital assistants (EDAs); portable media players (i.e., MP3-players, MP4-players, e.g., ipod); handheld video-game consoles (e.g., Playstation Portable [PSP], Nintendo DS); handheld and ultra-portable computers such as tablet PCs (e.g., iPad), and Smartbooks.

These devices have a range of functions from mobile cellular communication using text messages (SMS), photos and video (MMS), telephone, and World Wide Web access, to multi-media playback and software application support. Technological advances and improved computer processing power mean that single mobile devices such as smart phones and PDA phones are increasingly capable of high level performance in many or all of these functions.

The features of mobile technologies that may make them particularly appropriate for providing individual level support to health care consumers relate to their popularity, their mobility, and their technological capabilities. The popularity of mobile technologies has led to high and increasing ownership of mobile technologies, which means interventions can be delivered to large numbers of people. In 2009, more than two-thirds of the world's population owned a mobile phone and 4.2 trillion text messages were sent [15]. In many high-income countries, the number of mobile phone subscriptions outstrips the population [16]. In low-income countries, mobile communication technology is the fastest growing sector of the communications industry and geographical coverage is high [17]–[20].

Mobile technologies are mobile and popular, such that many people carry their mobile phone with them wherever they go. This allows temporal synchronisation of the intervention delivery and allows the intervention to claim people's attention when it is most relevant. For example, health care consumers can be sent messages designed to sustain their motivation to quit smoking throughout the day. Temporal synchronisation of the intervention delivery also allows interventions to be accessed or delivered within the relevant context, i.e., the intervention can be delivered at any time and extra support can be requested wherever and whenever it is needed. For example smokers trying to quit can send text messages requesting extra support while they are experiencing cravings due to withdrawal from nicotine, or those with asthma can access advice regarding how to increase the use of inhalers during an exacerbation of asthma.

The technological capabilities of mobile technologies are continuing to advance at a high pace. Current technological capabilities allow low cost interventions. There are potential economies of scale as it is technically easy to deliver interventions to large populations (for example, mobile technology applications can easily be downloaded and automated systems can deliver text messages to large numbers of people at low cost). The technological functions that have been utilised for health care consumers include text messages (SMS), software applications, and multiple media (SMS, photos) interventions. The technology supports interactivity, which allows people to obtain extra help when needed [21],[22]. Motivational messages, monitoring, and behaviour change tools used in face-to-face support can be modified for delivery via mobile phones. Interventions can be personalised with the content tailored to the age, sex, and ethnic group of the participant or to the issues they face [21],[22].

Existing systematic reviews of M-health interventions focus on the application of specific devices (e.g., mobile phones [23]–[27], specific mobile technology functions (e.g., text messaging [28]–[30]), or individual diseases or types of illness (e.g., diabetes care or chronic disease management [26],[30],[31]). These reviews require updating. Some types of interventions for health care consumers targeting healthy behaviour or disease management have not been covered by previous reviews. A comprehensive review of interventions delivered to health care consumers is lacking and provides a valuable overview of the existing evidence.

This systematic review aimed to quantify the effectiveness of mobile technology-based interventions delivered to health care consumers for health behaviour change and management of diseases.

## Methods

We adhered to our published plan of investigation as outlined in the study protocol [32].

Participants were men and women of any age. We included all controlled trials employing any mobile technology interventions (mobile phones; PDAs and PDA phones [e.g., BlackBerry, Palm Pilot]; smartphones [e.g., iphone]; enterprise digital assistants [EDAs]; portable media players [i.e., MP3-players and MP4-players, e.g., ipod]; handheld video-game consoles [e.g., Playstation Portable (PSP), Nintendo DS]; handheld and ultra-portable computers such as tablet PCs [e.g., ipad and Smartbooks]) to improve or promote health or health service use or quality. Trials were included regardless of publication status or language.

We included studies in which the intervention delivered by mobile technology was the primary intervention component under evaluation. We excluded studies evaluating either mixed mobile technology and non-mobile technology based interventions in which the treatment and control group both received the mobile technology-based component or interventions in which treatments between the treatment and control groups differed in additional ways besides the components delivered by mobile technology, such as interventions involving face-to-face counselling with a text message intervention compared to a control group receiving information only. We included trials of immunisation reminders; trials of general appointment reminders are reported elsewhere [33].

The interventions in trials meeting the inclusion criteria and targeting health behaviour change or disease management interventions for health care consumers are reported here. Other trials identified targeted data collection or health care delivery processes, such as those directed to health care providers (e.g., for diagnosis and management) or those involving communication between health care services and health care consumers (e.g., appointment reminders, test result notification). These are reported in two separate articles [33]. No trial was excluded from the review based on the type of health or health care service targeted.

Primary outcomes were defined as any objective measure of health or health service delivery or use. Secondary outcomes were defined as the following: self-reported outcomes related to health behaviours, disease management, health service delivery or use, and cognitive outcomes. Outcomes reported for any length of follow-up were included.

We searched the following electronic bibliographic databases MEDLINE, EMBASE, PsycINFO, Global Health, The Cochrane Library (Cochrane Database of Systematic Reviews, Cochrane Central Register of Controlled Trials [CENTRAL], Cochrane Methodology Register), NHS Health Technology Assessment Database, and Web of Science (science and social science citation index) from 1990 to Sept 2010 and the reference lists of included trials. The list of subheadings (MeSH) and textwords used in the search strategy can be found in Text S3. All of these terms were combined with the Cochrane Library MEDLINE filter for controlled trials of interventions.

Two reviewers independently scanned the electronic records to identify potentially eligible trials.

Two reviewers independently extracted data on number of randomised participants, intervention, intervention components, mobile devices employed, mobile technology functions used, sequence generation, allocation concealment, blinding of outcome assessors, completeness of follow-up, evidence of selective outcome reporting, any other potential sources of bias, and measures of effect using a standardised data extraction form. The authors were not blinded to authorship, journal of publication, or the trial results. All discrepancies were agreed by discussion with a third reviewer. The behaviour change techniques used in behaviour change interventions were classified according to Abraham and Michie's taxonomy of behaviour change techniques [34]. Risk of bias was assessed according to the criteria outlined by the International Cochrane Collaboration [35]. We assessed blinding of outcome assessors and data analysts and we used a cut off of 90% complete follow-up for low risk of bias for completeness of follow-up. We contacted study authors for additional information about the included studies, or for clarification of the study methods as required.

All analyses were conducted in STATA v 11. We calculated risk ratios and standard mean differences. We used random effects meta-analysis to give pooled estimates where there were two or more trials using the same mobile technology function (e.g., SMS messages) and targeting the same disease (e.g., diabetes control) or behaviour (e.g., activity) and reporting the same outcome. We examined heterogeneity visually by examining the forest plots and statistically using both the χ^2^ test and the *I* ^2^ statistic. We assessed evidence of publication bias using Funnel plots.

## Results

**Figure 1:**
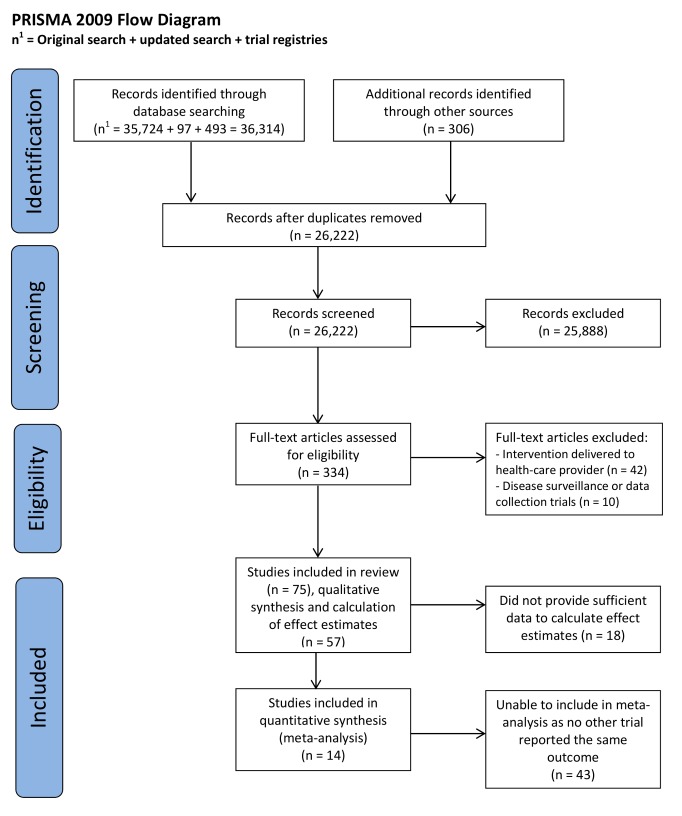
PRISMA 2009 flow diagram.

The combined search strategies identified 26,221 electronic records, which were screened for eligibility (Figure 1). The full texts of 334 potentially eligible reports were obtained for further assessment. Out of the 334 potentially eligible reports, 75 met the study inclusion criteria and were trials delivered to health care consumers; 26 interventions aimed to increase healthy behaviours, such as stopping smoking or increasing activity, which can prevent the onset or progression of disease (health behaviour change interventions); and 49 targeted disease management, including interventions designed to improve self-management of diseases like diabetes, and therapeutic interventions, such as interventions providing psychological support.

### Characteristics of Studies

#### Health behaviour change

The health behaviour change trials included 10,706 participants. Sample sizes ranged from 17 to 5,800. There were 26 randomised controlled trials with parallel groups, and one non-randomised controlled trial. There were five trials aiming to increase smoking cessation (Table 1), seven trials aimed to increase physical activity (Table 2), three trials aimed to reduce calorie intake (Table 2), seven trials aimed to increase physical activity and reduce calorie intake (Table 2), three trials aimed to increase safer sexual behaviour (Table 3), and one trial aimed to reduce alcohol consumption (Table 3).

**Table 1:** Description of trials of smoking cessation interventions. Health behaviour change: smoking-related studies.

Study	Study Design, Country, Device, Media	Participants	Aims	Intervention	Comparator
**Free 2011 ** [**36**]	Parallel group RCT; *Country:* UK; *Device*: Mobile telephone; *Media:* SMS	5,800 adult smokers aged ≥16 y. Mean age: 36.8 5 y (SD 11.05). Female 45%	To evaluate the effect of mobile phone-based SMS support on the point prevalence of smoking at 6 mo.	Participants received daily SMS before the quit date, then 5 SMS per day for 4 wk after the quit date. Between 4 and 26 wk participants received 3 SMS per week. Message content was tailored to participant interests and concerns about quitting smoking. Participants were offered a quit buddy contactable by mobile phone and an SMS craving helpline with an instant SMS response. The SMS system was fully automated. Duration: 26 wk.	Participants received fortnightly simple, short, generic SMS
**Free 2009 ** [**112**]	Parallel group RCT; *Country:* UK; *Device*: Mobile telephone; *Media:* SMS	200 adult smokers aged ≥16 y. Mean age: 36 y (SD 9.0). Female 38%	To evaluate the effect of mobile phone-based SMS support on the point prevalence of smoking at 4 wk.	Participants received daily SMS before the quit date, then 5 SMS per day for 4 wk after the quit date. Between 4 and 26 wk participants received 3 SMS per week. Message content was tailored to participant interests and concerns about quitting smoking. Participants were offered a quit buddy contactable by mobile phone and an SMS craving helpline with an instant SMS response. The SMS system was fully automated. Duration: 26 wk.	Participants received fortnightly simple, short, generic SMS.
**Haug 2009 ** [**113**]	Parallel group RCT *Country:* Germany; *Device:* Mobile telephone; *Media:* SMS	174 adult smokers. Mean age: Control 25.4 y (SD 4.9); Intervention 1 (1 SMS) 25.2 y (SD 4.8); Intervention 2 (3 SMS) 24.3 y (SD 3.8). Females: Control 63%; Intervention 1 56%; Intervention 2 52%.	Investigate the feasibility and acceptability of interactive mobile phone text messaging to support smoking cessation and the impact of different SMS frequency (intensity).	Participants received a weekly SMS with a question to assess their stage of change (transtheoretical model). Two intervention groups then received either 1 (1 SMS group) or 3 (3 SMS group) tailored feedback SMS per week. Participants attempting to quit had access to an SMS craving helpline which provided up to 60 tailored SMS responses. The SMS-COACH programme was fully automated. Duration: 14 wk	Participants received only the weekly SMS question to assess their stage of change (transtheoretical model).
**Rodgers 2005 ** [**39**]	Parallel group RCT *Country:* New Zealand; *Device:* Mobile telephone; *Media:* SMS	1.705 adult smokers aged ≥16 y. Mean age 25 y. Female 58%.	Assess the efficacy of SMS for supporting smoking cessation.	Participants set a quit date and received 5 SMS per day for 1 wk before and 4 wk after the quit date. Between 4 wk after the quit date and the end of the study (26 wk) participants received 3 messages per week. SMS contained information or advice on quitting smoking and some distractions (e.g.. sports news, quizzes. and polls/surveys). Participants received 1 mo of free outgoing text messages after their quit date. Participants were offered a quit buddy (another study participant) contactable by mobile telephone. Participants had access to an SMS craving helpline to receive an instant reply with tips on cravings. The SMS system was fully automated and a computer algorithm was used to match the SMS sent to the participant characteristics. Duration: 26 wk.	Participants only received one text message every 2 wk, thanking them for being in the study, providing study centre contact details, informing them that those who completed follow-up would be rewarded with a free month of text messaging (whether they quit or not), and reminding them of the time until their free month at the end of follow-up.
**Vidrine 2006 ** [**114**]	Parallel group RCT *Country:* USA; *Device:* Mobile telephone; *Media:* Voice	95 HIV positive adult smokers aged ≥18 y. Mean age: Control 43.1 y (SD 8.1); Intervention 42.6 y (SD 8.2). Females: Control 17%; Intervention 27.1%.	Assess the efficacy of a mobile telephone smoking cessation counselling intervention aimed at a multiethnic, economically disadvantaged HIV-positive population.	Participants set a quit date with their physician and received a personalized smoking cessation plan and a general self-help materials. Participants were given a prepaid mobile telephone and received 8 phone-counselling sessions during 2 mo. The counselling sessions were more often close to the quit date. Participants could also call the hotline when they needed additional smoking cessation support. The phone counselling and support was provided by a study researcher. Duration: 3 mo.	Participants received usual care: they set a quit date with their physician who offered a 10-wk supply of nicotine replacement therapy and received the personalized smoking cessation plan and a general self-help materials.

RCT, randomized controlled trial; SD, standard deviation.

**Table 2:** Description of trials of interventions targeting physical activity, diet, and physical activity and diet combined.

Intervention	Study	Study Design, Country, Device, Media	Participants	Aims	Intervention	Comparator
**Physical Activity**	**King 2008 ** [**115**]	Parallel group RCT; *Country:* USA; *Device:* PDA; *Media:* Application software	37 adults aged ≥50 y with low physical activity levels. Mean age: Control 59.6 y (SD 7.6); Intervention 60.7 y (SD 6.8). Females: Control 44%; Intervention 42%.	Investigate the effect of a behavioural intervention delivered by PDA on short-term changes in moderate- or vigorous intensity physical activity in underactive middle or older-aged adults	Participants monitored physical activity and motivation twice daily on a Dell Axim ×5. Participants set daily and weekly goals on the PDA, which provided textual and graphical feedback on progress to goals. The PDA had an alarm to remind participants to complete the short standardized survey on the PDA. Participants also received standard physical activity education materials at the beginning of the intervention. Participants received a pedometer to monitor physical activity and one introductory session on how to use the PDA. Duration: 8 wk	Participants received standard health educational written materials related to physical activity in middle- and older-aged adults.
	**Liu 2008 ** [**116**]	Parallel group RCT; *Country:* Taiwan; *Device:* Mobile telephone; *Media:* MP3/audio	60 male COPD patients aged 40–80 y in stable condition. Mean age: Control 72.8 y (SD 1.3); Intervention 71.4 y (SD 1.7)	Document the clinical efficacy, compliance and applicability of a home-based exercise training programme supervised via a mobile phone	Participants performed daily endurance exercise training by walking in speed with music played on a mobile phone. The tempo of the music was changed as appropriate every 3 mo as fitness changed. Duration: 1 y	Participants were provided with a home rehabilitation programme booklet and a DVD, including written instructions for home walking exercise training. Reinforcement telephone call every 2 wk for first 3 mo.
	**Newton 2009 ** [**42**]	Parallel group RCT; *Country:* New Zealand; *Device:* Mobile telephone; *Media:* SMS	78 adolescents aged 11–18 y; attending outpatient clinic in 4 regional adolescent diabetes services in New Zealand	To assess whether pedometers and text messaging increase physical activity in adolescents with type 1 diabetes	Participants wore an open pedometer with a daily goal of at least 10,000 steps. Participants received a weekly motivational text message reminding them to wear the pedometer and be active. Duration: 12 wk	Standard diabetes care.
	**Nguyen 2009 ** [**117**]	Parallel group RCT; *Country:* USA; *Device:* PDA phone; *Media:* Application software, SMS, Voice	17 COPD patients aged 40 y with stable condition. Mean age: Intervention 1 (Mobile-C) 72 y (SD 9); Intervention 2 (Mobile-SM) 64 y (SD 12). Females: Intervention 1 67%; Intervention 2 63%	Determine the feasibility and efficacy of a mobile phone-based exercise persistence intervention for patients with COPD following completion of pulmonary rehabilitation	Participants were asked to do 150 min of moderate-intensity exercise per week in 3 to 5 sessions and received booklets with exercise tops, local resources, and pictures of exercises. Two intervention groups: (1) submitted daily information about their COPD symptoms and exercise via SMS on the PDA phone and received 1 SMS reply per week to thank them for the information only; (2) submitted COPD symptom and exercise information and received personalized weekly feedback and encouragement SMS written by the study nurse or telephone calls to discuss problems in more detail. Duration: 6 mo	None
	**Prestwich 2009 ** [**118**]	Parallel group RCT; *Country:* UK; *Device:* Mobile telephone; *Media:* SMS	Exercise less than 3 times per week; own mobile telephone; fluent in English; aged 18–40 y	Test whether the effects of implementation intentions on exercise can be strengthened by combining them with text message reminders	Participants read recommendations about exercising for 20 min at least 3 times per week. Three intervention groups: (1) formed implementation intentions for achieving the exercise goal and were offered opportunity to receive SMS reminder of these plans; (2) SMS reminders to exercise (no implementation intentions formed); (3) formed implementation intentions for achieving the exercise goal only. Duration: 4 wk	Two control groups: (1) no treatment; (2) read recommendations about exercising for 20 min at least 3 times per week and were informed of the benefits of forming implementation intentions (but were not asked to form any).
	**Prestwich 2010 ** [**96**]	Parallel group RCT; *Country*; UK *Device:* Mobile telephone; *Media:* SMS	149 adults with low physical activity levels. Mean age: Control 23.6 y (SD 4.5); Intervention 1 (SMS plan reminder) 22.2 y (SD 5.0); Intervention 2 (SMS goal reminder) 24.4 y (SD 6.9). Females: Control 68%; Intervention 1 56%; Intervention 2 64%.	Test whether interventions that paired implementation intentions with text messages reminders of plans or goals increased brisk walking in a student-based sample	Participants set goals for and formed implementation intentions for walking for at least 3 min 5 or more days per week. Two intervention groups: (1) received SMS reminders of the plans; (2) received SMS reminders of the brisk walking goal. Participants received at least 1 SMS reminder during the 4-wk study and could change the content, time and frequency of SMS through a secure website. Duration: 4 wk	Participants were asked to try to walk briskly for at least 3 min on ≥5 d per week to meet recommended physical activity levels.
	**Sirriyeh 2010 ** [**94**]	Parallel group RCT; *Country:* UK; *Device:* Mobile telephone; *Media:* SMS	120 participants aged between 16 and 19 y, in full time further education and in possession of a personal mobile phone.	To develop and pilot the feasibility and efficacy of a novel intervention using affective messages as a strategy to increase physical activity levels in adolescents.	The SMS text messages for the **single instrumental intervention group** included statements regarding the instrumental gains associated with regular moderate and vigorous physical activity. Examples of these messages are, “Physical activity can help maintain a healthy weight. What activity will you do today?” and “Physical activity can keep your heart healthy. What activity will you do today?” **For the combined affective and instrumental intervention group**, SMS text messages included statements regarding either the affective, or instrumental, gains associated with regular moderate and vigorous physical activity. An equal number of messages from interventions 1 and 2 were presented in the intervention period. Duration: 2 wk.	Participants also received SMS text messages over the same 14-d period. However, this was limited to two messages (1 per week). The content of these messages was neutral, using only the final element of the phrase used in the intervention groups, “What activity will you do today?” for comparability.
**Diet**	**Atienza 2008 ** [**119**]	Parallel group RCT; *Country:* USA; *Device:* PDA; *Media:* Application software	36 adults aged ≥50 y. Mean age: Control 58 y (SD 5.8); Intervention 63.2 y (SD 9.1). Females: Control 70%; Intervention 69%.	Evaluate the efficacy of a PDA for increasing vegetable and whole-grain intake in middle and older aged adults	Participants received a Dell Axim ×5 PDA and used it to record consumption of vegetables and whole grains and contextual information twice daily. The PDA was set with an alarm to remind participants to complete the diary entry. Participants received standard nutrition-education materials as recommended by national health organizations. All participants were given goals of 5 servings of vegetables and 3 servings of whole grains per day. Duration: 8 wk	Participants received standard written materials related to nutrition in middle-aged and older adults as recommended by national health organizations
	**Beasley 2008 ** [**41**]	Parallel group RCT; *Country:* USA; *Device:* PDA;	174 overweight (BMI 25–40 kg/m^2^) adults. Mean age: Control 54 y (SD 10); Intervention 52 y (SD 12). Females: Control 77%; Intervention 83%.	Evaluate effectiveness of DietMatePro on adherence to a diet regimen and compare concordance on energy and nutrient intakes with a paper diary	Participants recorded all food and drink intake daily on the PDA which had a database of food nutritional information installed and had personalized calorie and nutritional intake targets. The PDA provided feedback on progress towards targets and recipe and meal plans. Participants received a food portion education leaflet and two commercial diet books. The PDA had an alarm set for meal times. Duration: 4 wk	Participants received a 31-page food diary booklet to record all food and drink intake. Participants were given personalized calorie and nutritional intake targets and all the supplementary information given to the intervention group (leaflet and books).
	**Ellrott 2005 ** [**120**]	Parallel group RCT; *Country:* Germany; *Device:* Other handheld computer	85 overweight (BMI 25–30 kg/m^2^) women aged 30–60 y.	To determine the effect of a portable computer on the success of and compliance to a self-help weight-loss programme from the German Society for Nutrition	Participants received a handheld computer (Mealus) and used it to record all food and drink consumed. The handheld computer was installed with a database of the nutrient composition of foods. The computer provided instant summaries of recorded calorie and nutrient intake and recommended intake based on age, weight, and desired weight change. Participants received the German Society for Nutrition self-help manual.	Participants recorded food and drink intake in a paper diary. Participants received the German Society for Nutrition self-help manual.
**Diet and Physical Activity**	**Burke 2010 ** [**40**]	Parallel group RCT; *Country:* USA; *Device:* PDA; *Media:* Application software	210 individuals between 18 and 59 y of age and had a BMI between 27 and 43 kg/m2. Mean age: Control = 47.4%; Intervention = 46.7%. Males: Control = 15.3%; Intervention = 14.7%	To determine whether self-monitoring diet and exercise using a PDA, with or without tailored feedback (PDA or PDA+feedback), was superior to using a PR for promoting and maintaining weight loss.	All three treatment groups received the same standard behavioural intervention. The intervention included: (i) daily self-monitoring of eating and exercise behaviours, (ii) group sessions, (iii) daily dietary goals, and (iv) weekly exercise goals. Participants in the PDA and PDA+feedback groups were provided with PDAs with self-monitoring software that tracked energy and fat consumption and displayed current intake related to daily goals and also provided easily accessed nutrition information. Participants in the PDA+feedback group had a custom software program on their PDAs with a feedback algorithm that provided daily messages tailored to their entries and provided positive reinforcement and guidance for goal attainment. The feedback messages varied by the time of day and conditions of reported intake, e.g., if between 10:00 am and noon, a participant had reported consuming >40% of the calorie allowance but only 20%–40% of the fat goal, a sample message could be “Good job making choices low in fat. Watch portion sizes to control calories.” The feedback messages focused on diet could be delivered between 10:00 am and 9:00 pm.	Participants in the PR group were given standard paper diaries and instructed to record all foods eaten, the calories, and fat grams, as well as minutes of exercise. At the first group session, they were given a reference book that contained nutrition information and were trained in how to determine the calorie and fat gram content of their foods.
	**Haapala 2009 ** [**121**]	Parallel group RCT; *Country:* Finland; *Device:* Mobile telephone; *Media:* SMS	125 overweight (BMI 25–36 kg/m^2^) adults aged 25–44 y. Mean age: Control 38 y (SD 4.7); Intervention 38.1 y (SD 4.7). Females: Control 76%; Intervention 79%.	Investigate the effectiveness of a mobile phone weight-loss programme among healthy overweight adults	Participants received a daily automated SMS indicating a daily target weight and progress to that goal, the percent reduction in food consumption compared to normal diet and total target Kcals, days remaining until reaching target weight. Participants also received SMS tips on how to reduce calorie intake/increase physical activity. Participants could adjust the target weight at 3-monthly clinic visits. Participants also had access to a secure website to view weight loss progress. Duration: 1 y.	No treatment
	**Patrick 2009 ** [**122**]	Parallel group RCT; *Device:* Mobile telephone, MMS; *Media:* SMS	78 overweight (BMI≥25–39.9) adults aged 25–55 y. Mean age: Control 42.4 y (SD 7.5); Intervention 47.4 y (SD 7.1). Females: Control 84%; Intervention 76%.	Investigate the effectiveness of daily text messages to prompt behaviour, support, and aid self-monitoring in overweight adults trying to reduce their body weight	Participants received daily automated SMS in behavioural and dietary strategies to support weight control. The topic changed weekly with a database of 3,000 unique messages. Participants reported their weight weekly by SMS and set goals for weight loss/dietary change. Participants received a manual on nutrition and behavioural strategies. A health counsellor called participants for 5–15 min each month to discuss progress and problems. Duration: 4 mo	Participants received short printed materials once a month for 4 mo providing information on weight loss, nutrition, and physical activity.
	**Shapiro 2008 ** [**123**]	Parallel group RCT; *Country:* USA; *Device:* Mobile telephone; *Media:* SMS	58 children aged 5–13 y. Mean age: Control 1 (group sessions) 8.5 y (SD 2.3); Control 2 (group sessions and paper diary) 9.3 y (SD 2.2); Intervention 8.4 y (SD 2.3). Females: Control 59%; Intervention 72%.	Assess the impact of SMS on self-monitoring behaviours related to weight management in children and on dietary and physical activity behaviour change	Families attended 3 educational group sessions on increasing physical activity, reducing sugar-sweetened beverage consumption and screen time (computer or television). Parent and child pairs were instructed to send 2 SMS daily to the study team, reporting separately for the parent and child: the number of steps taken (measured by pedometer); the number of sugar-sweetened beverages consumed; the minutes of screen time. Parents and children received automated feedback messages selected from a database using an algorithm based on the number of goals met, comparison to the previous day. Duration: 8 wk.	Families attended the same educational group sessions as the intervention group. There were 2 control groups: (1) attended group sessions only; and (2) attended group sessions and recorded target behaviours in a paper diary
	**Shay 2009 ** [**124**]	Parallel group RCT; *Country:* USA; *Device:* PDA; *Media:* Custom software	73 overweight (BMI ≥25.0 kg/m^2^) military personnel in active duty enrolled in Navy weight management program. Mean age: Control 1 (paper diary) 36.5 y (SD 9.6); Control 2 (web diary) 35.7 y (SD 10.5); Intervention 34 y (SD 8.5). Females: Control 1 46%; Control 2 25%; Intervention 50%.	Investigate the effect of diary preference on adherence to a food/exercise diary self-efficacy and change in body composition	Participants received a PDA (Tungsten/e2 Palm with Calorie King Palm OS program installed, which provided calorie content of foods and exercise calorie expenditure. Participants recorded all physical activity and food and drink intake daily. Duration: 12 wk	Two control groups: (1) paper diary and 302-page booklet of nutritional information of foods and calorie expenditure during exercise; (2) web diary with same information as on the PDA given to the intervention group. Participants recorded all physical activity and food and drink intake daily.
	**Stewart Agras 1990 ** [**125**]	Parallel group RCT; *Country:* USA; *Device:* Other handheld computer	90 overweight (BMI 25–35) women aged ≥18 y. Mean age 45.2 y (SD 12.4)	Evaluate the effectiveness of a commercially available hand-held computer with therapist support for supporting weight loss	Participants received Casio PB-700, which they used to set daily goals for calorie intake and exercise and to enter calorie information for snacks and meals during the day (using a handbook containing the calorie content of common foods). The computer provided instant calorie intake and exercise summaries at any time. Other functions included: meal planning; random supportive electronic messages; long term graphical monitoring. Two intervention groups: (1) computer plus one introductory weight-loss group session; (2) computer plus introductory session and 4 more group sessions on technical difficulties and tried interventions on weight loss, diet, and exercise. Duration: 12 wk	Participants attended 10 group weight-loss therapy sessions over 12 wk following a standard treatment manual
	**Turner-Mcgrievy 2009 ** [**126**]	Parallel group RCT; *Country:* USA; *Device:* Portable media player; *Media:* MP3/audio	94 overweight (BMI 25–40) adults. Mean age: Control 39.6 y (SD 12.2); Intervention 41 y (SD 11.8). Females: Control 81%; Intervention 68%.	Compare the effects on body weight change of a weight-loss podcast based on health behaviour change theories to a web-available podcast with no theoretical basis	Participants received two podcasts per week for aiding weight-loss, designed using social cognitive theory constructs (expectancies, expectations, self-efficacy, behavioural capability). Participants were shown how to download podcasts at an introductory session. Participants received a book on nutritional content of popular foods. Duration: 12 wk.	Participants received a currently available weight-loss podcast (2 per week) considered to be accurate and popular based on a content analysis.

BMI, body mass index; COPD, chronic obstructive pulmonary disease; RCT, randomized controlled trial; SD, standard deviation.

**Table 3:** Description of trials of sexual health and alcohol reduction interventions.

Intervention	Study	Study Design, Country, Device, Media	Participants	Aims	Intervention	Comparator
**Sexual health**	**Delamere 2006 ** [**127**]	Parallel group RCT; *Country:* Ireland; *Device:* Mobile telephone; *Media:* SMS	60 young people aged 17–18 y attending a sexual health clinic	Determine the acceptability and impact of text messages to promote condom use in adolescents.	Participants received weekly SMS reminding them to use a condom. SMS were written and sent by the study team. Duration: 3 mo.	No treatment
	**Jones 2008 ** [**128**]	Non-randomised parallel group trial; *Country:* USA; *Device:* Other handheld computer; *Media:* MP4/video	76 sexually active women aged 18–29 y. Mean age: 21 y.	Determine the effectiveness of a video based on sex script theory to reduce women's expectations of having to engage in unprotected sex with male partners.	Participants viewed the video, a Story about Toni, Mike, and Valerie based on sex script theory and the theory of power as knowing participation in change, on a handheld computer (Sony Vaio UX series).Video duration: 43 min.	Participants viewed a 43-min video describing careers in health care and computer technology. on a handheld computer (Sony Vaio UX series).
	**Lim 2007 ** [**129**]	Parallel group RCT; *Country:* Australia; *Device:* Mobile telephone; *Media:* SMS	994 people aged 16–29, recruited at a music festival in Melbourne.	To trial a novel method of sexual health promotion - sending email and SMS messages about safe sex and STI to promote reduction in STI behaviours and increases in STI knowledge and testing.	Text messages were sent every 3–4 wk for a 12-mo period and included catchy STI prevention slogans. Emails were also sent monthly, and contained detailed information about STI topics and links to related websites. Duration: 1 y	No treatment
**Alcohol**	**Weitzel 2007 ** [**130**]	Parallel group RCT; *Country*: USA; *Device:* Other handheld computer; *Media*: SMS	40 university/college students drinking >1 time per week aged ≥18 y. Mean age 19.2 y. Female 55%.	Assess the feasibility and effectiveness of daily tailored health messages delivered on wireless handheld computers to promote changes in alcohol-related attitudes and behaviours and whether messages change survey adherence.	Participants completed daily questionnaires on the handheld computer about alcohol consumption during the previous day, with email and telephone reminders if the survey was not completed. Two intervention groups: (1) handheld computer questionnaire only; (2) handheld computer questionnaire plus daily messages to the handheld computer about avoiding alcohol-related negative consequences tailored to the questionnaire responses. Messages were sent manually by the research team from a pre-developed database of message content. Duration: 2 wk.	None

MED, mobile electronic device; RCT, randomized controlled trial; STI, sexually transmitted infection.

All trials were conducted in high-income countries.

#### Disease management

The 49 disease management trials recruited 6,832 participants with sample sizes ranging from 16 to 273 participants. Thirty-four were randomised controlled trials with parallel groups, three were cluster randomised controlled trials, five were crossover trials, and seven were non-randomised parallel group trials. Nine trials aimed to improve the management of illnesses presenting acutely. Of these nine trials, seven provided cardiopulmonary resuscitation instruction (Table 4), one provided the information required by a lay-helper to manage an emergency, and one provided accident and emergency department discharge information for sprains and lacerations. For chronic conditions six trials aimed to increase adherence to medication (Table 5). Thirteen trials aimed to improve diabetes control (Table 6), six aimed to improve asthma control (Table 7), and two aimed to improve hypertension control (Table 8). Seven interventions provided psychological support (Table 9). One trial aimed to increase self-care behaviours in patients following a lung transplant, one trial aimed to improve monitoring and provide appropriate advice regarding chemotherapy-related toxicity symptoms, one trial aimed to monitor vital parameters (e.g., blood pressure, heart rate, weight) and provide appropriate advice to people with heart failure, one trial aimed to monitor home care blood transfusions in people with severe haemophilia so the clinic could be alerted regarding “significant” bleeds. One trial aimed to improve the recall of rehabilitation goals in people with acquired head injury by sending text messages regarding their goals. These varied trials are described in Table 10.

**Table 4:** Description of trials of cardiopulmonary resuscitation interventions.

Intervention	Study	Study Design, Country, Device, Media	Participants	Aims	Intervention	Comparator
**CPR**	**Choa 2009 ** [**131**]	Parallel group RCT; *Country:* Korea; *Device:* Mobile telephone; *Media:* Custom software	84 non-medics trained in CPR.	To compare the skill retention of two groups of lay persons 6 mo after their last CPR training. Mean age: Control = 31 (Range: 23–57), 44.7% Male; Intervention = 31.4 (Range: 22–53), 47.6% Male	As the participants from both groups entered the room, they were instructed to perform CPR to a manikin positioned in front of them according to the instructions told during training. The intervention group completed 3 rounds of CPR with the aid of a cellular phone video clip.	The control group was instructed to perform CPR without any assistance
	**Yang 2009 ** [**132**]	Parallel group RCT; *Country:* Taiwan; *Device:* Mobile telephone; *Media:* MP3/audio, MP4/video	96 Mandarin Chinese speakers, ≥16 y with no CPR training in the last 5 y.	To assess the effect of adding interactive video communication to dispatch instruction on the quality of bystander chest compressions in simulated cardiac arrests. Mean age: Control = 50.4 (SD: 12.7); Intervention = 50.1 (SD: 11.5)	All candidates were taken into a study room where they confronted the standardised scenario of a collapsed adult male on the floor in cardiac arrest. The subjects received standardised briefing of the scenario. Intervention group received interactive voice and video demonstration and feedback. Duration: 4 min.	Same as intervention, but the phone used allowed these participants only to get voice CPR instructions.
	**Bolle 2009 ** [**133**]	Non-randomised parallel group trial; *Country:* Norway; *Device:* Mobile telephone; *Media:*MP4/video	60 high school students.	To determine if video communication could improve the quality of lay people CPR by enhancing communication between bystanders and dispatchers during telephone CPR (t-CPR). Mean age: Control = 17.9 (Range: 16–32); Intervention = 17.3 (Range: 15–34), Male: Control 34.4%; Intervention 26.7%.	Intervention group communicated via video call with experienced nurse dispatchers at a hospital emergency medical dispatch centre. CPR was performed on a recording resuscitation manikin during simulated cardiac arrest. Duration: 10 min.	Control group communicated via normal audio call with experienced nurse dispatchers at a hospital emergency medical dispatch centre. CPR was performed on a recording resuscitation manikin during simulated cardiac arrest.
	**Choa 2008 ** [**131**]	Cluster RCT; *Country:* Korea; *Device:* Mobile telephone; *Media:* MP3/audio, MP4/video	16 hospital employees, who attended a mandatory CPR training course.	Compare the effectiveness of animation-assisted CPR instruction with dispatcher-assisted instruction in participants with no previous CPR training. Mean age: Control = 28.4 (Range 21–54); Intervention = 28.1 (Range: 20–49). Male: Control 48.7%; Intervention: 45.4%	The participants of the study were instructed to perform three CPR cycles on a manikin according to the instruction provided by audiovisual animation through a cellular phone.	The participants of the study were instructed to perform three CPR cycles on a manikin according to the instruction provided by voice through a cellular phone.
	**Zanner 2007 ** [**132**]	Parallel group RCT; *Country:* Germany; *Device:* Mobile telephone; *Media:* Application software	119 of the general population, mostly final year high school students.	To evaluate the possible benefit of a mobile phone application (M-AID) in the setting of an out-of-hospital cardiac arrest. Mean age: Control = 22 (Range: 14–33), 65.6% Male; Intervention = 20 (Range: 15–53), 56.4% Male.	The participants were confronted with an actor complaining of acute onset chest pain radiating in the left arm and the neck, dyspnoea, and anxiety. After 1 min the actor became unconscious due to cardiac arrest and the participants were redirected to a manikin for further action. The intervention group was equipped with a mobile phone running the software, which they were instructed to use for support whenever they thought it beneficial. The software consisted of 59 pages linked through simple “yes” or “no” questions according to emergency situation/first aid guidelines.	Participants in the control group had to deal with the emergency situation without support.
	**Yang 2008 ** [**133**]	Parallel group RCT; *Country:* Taiwan; *Device:* Mobile telephone; *Media:* MP4/video	96 volunteers >16 y who have not received any CPR training within the last 5 y.	To assess the impact of adding interactive video communication to dispatch instructions on the quality of rescue breathing in simulated cardiac arrests. Mean age: Control = 50.4 (SD: 12.7); Intervention = 50.1 (SD: 11.5)	Subjects confronted the standardized scenario of a collapsed adult male on the floor in cardiac arrest. They received standardized briefing on the case scenario and instruction to seek help from an EMS dispatcher using the study cell phone already set up. Intervention group received simultaneous video and voice communication between the caller and the dispatcher.	Control group: telephone instructions were given by the simulated dispatcher via the cell phone.
	**Merchant 2010 ** [**87**]	Parallel group RCT; *Country:* USA; *Device:* Mobile telephone; *Media:* Voice	160 veterans or caregiver of a veteran aged 18–60 y.	To evaluate the extent to which prerecorded audio CPR instructions delivered by a cell telephone will improve the quality of CPR provided by untrained and trained lay rescuers.	Individuals randomized to the telephone group were informed that holding down the number 1 key on the telephone would speed-dial a number with prerecorded CPR instructions. All participants were asked to pretend that the manikin was a person who had suddenly collapsed. They were to assume that 911 was being called and that they should demonstrate what they would do to help the person while awaiting arrival of EMS.	Similar scenario, except control participants were not given the opportunity to hear CPR prompts/instructions on a telephone.
**Other**	**Ertl 2007 ** [**134**]	Parallel group RCT; *Country:* Germany; *Device:* PDA phone; *Media:* Custom software	101 members of associations and sports clubs	To determine if the knowledge required by lay-helper for the management of an emergency could be provided by an expert system on a PDA and if the use of this system leads to a significant improvement in quality of pre-hospital emergency care.	Both groups were confronted with two standard emergency situations. The intervention group acted with the help of the system on the PDA which provided an interactive audible and visual display of instructions. The core principle of the system was the stepwise guidance through checklists for emergency care. Duration: 15 min.	The control group acted only with their current knowledge.

CPR, cardiopulmonary resuscitation; EMS, emergency medical services; RCT, randomized controlled trial; SD, standard deviation.

**Table 5:** Description of trials targeting medication adherence.

Study	Study Design, Country, Device, Media	Participants	Aims	Intervention	Comparator
**Armstrong 2009 ** [**136**]	Parallel group RCT; *Country:* USA; *Device:* Mobile telephone; *Media:* SMS	70 adults aged ≥18 y. Mean age: Control 34.3 y (SD 14.2); Intervention 32.9 y (SD 13.4). Males: Control 29%; Intervention 31%.	Evaluate the effectiveness of text messaging as a reminder tool for improving adherence to sunscreen application.	Participants received daily text message reminders to apply sunscreen. Duration: 6 wk.	No treatment
**Cocosila 2009 ** [**137**]	Parallel group RCT; *Country:* Canada; *Device:* Mobile telephone; *Media:* SMS	102 adults aged ≥18 y. Mean age: Control 23.9 y (SD 7); Intervention 23.8 y (SD 7.3). Males: Control 46%; Intervention 44%.	Determine whether SMS reminders increase adherence to vitamin C in healthy people	Participants were sent a daily SMS reminder to take the vitamin C tablet. Participants were asked to reply with one letter by SMS to acknowledge that they had taken the tablet. If participants responded they received a subsequent SMS reinforcing the behaviour. If participants did not reply, they received a correcting SMS reminding them of benefits of vitamin C. Frequency of messages reduced to every other day in the last 2 wk of the study. Duration: 1 mo	No treatment
**Lester 2010 ** [**37**]	Parallel group RCT; *Country:* Kenya; *Device:* Mobile telephone; *Media:* SMS	538 patients initiating ART recruited from three different HIV clinics that are involved in intense ART provision scale-up. Mean age: Control 36.6 (SD 7.9); Intervention 36.7 (SD 8.5). Males: Control 44%; Intervention 45%	To assess whether mobile phone communication between health-care workers and patients starting antiretroviral therapy in Kenya improved drug adherence and suppression of plasma HIV-1 RNA load.	A text message via SMS was sent each week to inquire about their status and thus to remind them about the availability of phone-based support. Typically, the slogan “Mambo?” was sent, which is Kiswahili for “How are you?” The health workers used multiple recipient (bulk) messaging functions to improve efficiency. Patients in the intervention group were instructed to respond within 48 h that either they were doing well (“Sawa”) or that they had a problem (“Shida”). The clinician then called patients who said they had a problem or who failed to respond within 2 d. Participants were instructed that healthcare workers were available to respond during clinic hours only.	Usual care
**Ollivier 2009 ** [**138**]	Parallel group RCT; *Country:* France; *Device:* Mobile telephone; *Media:* SMS	424 military personnel returning from duty in a malaria-endemic region.	Assess the impact of a daily SMS reminder about malaria chemoprophylaxis for soldiers. Assess feasibility and acceptability	A commercial SMS messaging service was used to send a standardized automated daily message at midday reminding participants to take their prophylactic doxycycline pill with advice to contact a physician if any fever occurs. Duration: 27 d	No treatment
**Vilella 2004 ** [**139**]	Non-randomised parallel group trial; *Country:* Spain; *Device:* Mobile telephone; *Media:* SMS	2,349 travellers who attended the clinic to start a vaccination course with the hepatitis A+B vaccine and the hepatitis A vaccine, who were 18 y and older.	The effectiveness of an intervention measure was evaluated, namely, text messaging to the mobile phone, with a view to obtaining stricter compliance with a medical prescription: the vaccination schedule.	The travellers received the SMS a few days before the date foreseen, that is, for the reminder of the second hepatitis A+B dose, within 30 d of the primary dose, and for the second hepatitis A dose and the third hepatitis A+B dose within 6 mo of the primary dose. Duration: 4 mo.	No reminder.
**Yang 2008 ** [**140**]	Parallel group RCT; *Country:* USA; *Device:* Mobile telephone; *Media:* SMS	201 patients receiving a new prescription	Determine whether SMS text messaging (and also email or telephone calls) increase patient compliance to prescribed antibiotics	Participants received one SMS on day 4 of the 10-d regimen reminding them to take the antibiotic medication Duration: 9 d.	No treatment

RCT, randomized controlled trial; SD, standard deviation.

**Table 6:** Description of trials of diabetes management interventions.

Study	Study Design, Country, Device, Media	Participants	Aims	Interventions	Comparators
**Benhamou 2007 ** [**141**]	Crossover RCT; *Country:* France; *Device:* Mobile telephone, PDA; *Media:* SMS	30 patients with poorly controlled diabetes. Mean age: 41.3 y	To assess the relevance of telecare in adult patients with type 1 diabetes under continuous subcutaneous insulin infusion.	Patients were requested to download their blood glucose values at weekly intervals during 1 y, and to download the quality of life questionnaire every 3 mo, within 1 wk before of after the visit at the clinic. Investigators acknowledged reception of glucose data and sent therapeutic advice through SMS at weekly intervals. Duration: 1 y.	In the control condition patients did not receive SMS.
**Cho 2009 ** [**134**]	Parallel group RCT; *Country:* Korea; *Device:* Mobile telephone; *Media:* SMS	75 patients with type 2 diabetes mellitus. Mean age: Control = 45.2 (SD: 11.3); Intervention = 51.1 (SD: 13.2).	To compare the effect of a mobile phone with a glucometer integrated into the battery pack (the “Diabetes Phone”) on management of type 2 diabetes to the IBGMS.	The diabetes phone is a mobile phone containing a device to measure capillary blood glucose on site and transmit blood glucose data to a web server automatically without manual input. One doctor reviewed all the information about self monitoring BG data onindividual web-based charts at least once every other week and sent recommendations to the phone group by SMS on the diabetes phone. Patients in both groups received visual displays with graphs showing blood glucose levels and the degree of glucose control for the previous 24 h, 1 wk, and 1 mo. Duration: 3 mo	Participants in the control group were taught about accessing and using the specialized, web-based diabetes patient management system and how to communicate with a management team through their individualized, web-based charts on the Internet website, at least once every other week.
**Faridi 2008 ** [**44**]	Cluster RCT; *Country:* USA; *Device:* Mobile telephone; *Media:* SMS	30 adults with diabetes controlled by either diet or oral medications and a BMI >25 kg/m^2^. Mean age: Control = 56.7 (SD: 10.6); Intervention = 55.3 (SD: 8.7).	To examine the feasibility of using the NICHE technology to assist with diabetes self-care management in a clinical population and determine whether the NICHE technology improves type 2 diabetes patients' health outcomes.	Intervention patients were taught how to use the NICHE system for type 2 diabetes management. The technology uses wireless remote technology to provide tailored feedback and reminders based on patient-specific data to patients and providers via messages on the cell phone. Patients were required to test their glucose once daily (upon awakening) and wear their pedometers during the day. Subjects were required to upload data onto the NICHE server once daily. They received tailored messages via mobile phone based on the uploaded data. Duration: 3 mo.	Control subjects continued with standard diabetes self-management and tracked their step count using a pedometer.
**Franklin 2006 ** [**135**]	Parallel group RCT; *Country:* Scotland; *Device:* Mobile telephone; *Media:* SMS	60 patients with type 1 diabetes on conventional insulin therapy. Mean age: Control = 12.7 (IR: 10.5–14.8); Intervention = 14.1 (IR: 11.7–15.6).	To assess Sweet Talk, a text-messaging support system designed to enhance self-efficacy, facilitate uptake of insulin therapy and improve glycaemic control in paediatric patients with type 1 diabetes.	All patients continued with conventional care during the study. Those allocated to the Sweet Talk intervention participated in goal setting at clinic visits. They were also given a card detailing the functions of the text-messaging service. Duration: 12 mo	All patients continued with conventional care including 3–4-monthly clinic visits and access to an emergency hotline.
**Hanauer 2009 ** [**136**]	Parallel group RCT; *Country:* USA; *Device:* Mobile telephone; *Media:* SMS	40 insulin-treated patients, 12–25 y old. Had an SMS capable phone and home internet with email access. Mean age: Control = 18.2 (SD: 2.3); Intervention = 17.7 (SD: 3).	To test the feasibility of implementing a fully automated, two way test messaging system to encourage increased BG monitoring in teens and young adults with diabetes using a CARDS.	CARDS would send cell phone text message to remind participants to check their BG. If CARDS did not receive a response form the participants within 15 min a single repeat reminder was sent. Participants could also submit BG values directly onto the website. Duration: 3 mo	Control group received emails from CARDS.
**Holman 1996 ** [**137**]	Crossover RCT; *Country:* UK; *Device:* Other handheld computer; *Media:* Custom software	12 patients with established insulin-dependent diabetes. Mean age: 33.8 y (SD: 11.7).	To evaluate a POIRO.	The intervention is a hand-held computer that supports algorithms based on published clinical guidelines. Before each meal patients are asked to record their blood glucose values, anticipated meal size, expected post meal exercise level, and state of health together with time and severity of any hypoglycaemic episodes that may have occurred. Insulin dose adjustments are made according to algorithms taking into account physician's dose and earlier behaviour (i.e., earlier insulin injections, meal size etc). For the duration of the study, patients were requested to test their blood glucose at least 4 times a day. During the intervention 3-wk study period the computerised insulin dose adviser was on. Duration: 3 wk.	As intervention but the computerised insulin dose adviser on the MED was turned off.
**Istepanian 2009 ** [**138**]	Parallel group RCT; *Country:* UK; *Device:* Mobile telephone; *Media:* WAP	137 patients with diabetes. Mean age: Control = 57 y (SD: 13); Intervention = 60 y (SD: 12).	To evaluate an m-health system against usual care in an unselected population of patients with mainly type 2 diabetes.	Patients in the telemonitoring arm were trained to self measure capillary blood sugar. The monitor was adapted to transmit their recordings wirelessly by Bluetooth to a mobile phone. Duration: 9 mo	Patients in the control group did not use a mobile phone to transmit data. They received their care from the diabetes centre and/or the local practitioners.
**Kim 2007 ** [**139**]	Parallel group RCT; *Country:* South Korea; *Device:* Mobile telephone; *Media:* SMS	80 type 2 diabetic patients. Mean Age: 48.1 y	To compare biochemical profiles and clinical status between diabetic patients who used researcher's system for 12 wk and those who received the conventional outpatient management over the same time period.	A knowledge matrix containing information on proper diet and exercise for diabetic patients was developed. A device that had the dual function of a glucometer and a pedometer when connected to the patient's cellular phone transmitted data on the device automatically to participants personal data sheet on a website. Patients were asked to keep a record on the website of how much and what kind of food they ate, as well as how much they exercised. When these data were sent to the main menu, researcher's system automatically composed messages that were then sent to the patient. In addition, patients could check their clinical data by logging into the website where they could obtain information on diabetes and incorporate the information into their daily lives for better self- management of diabetes. Patients in the intervention group were taught how to use system for 12 wk without any outpatient visits.	Patients in the control group were provided with glucometers and received their usual outpatient management from their physicians.
**Quinn 2008 ** [**140**]	Parallel group RCT; *Country:* USA; *Device:* Mobile telephone; *Media:* Application software	30 18–70 y olds, who had a diagnosis of type 2 diabetes for at least 6 mo.	To test the feasibility of a cell phone-based diabetes management software system used in conjunction with web-based data analytics and therapy optimization tools. To assess the usability and impact of a remote BG-monitoring system on patient HBA1C outcomes and HCPs prescribing behaviours.	Intervention group received a Bluetooth-enabled One Touch Ultra BG meter, adequate BG testing strips and lancets for the duration of the trial and a cell phone equipped with WellDoc's proprietary DiabetesManager software. Their log books were sent electronically to their HCPs by the WellDoc System every 4 wk or sooner if needed. Duration: 3 mo	Control group patients received One Touch Ultra BG Meters and adequate BG testing strips and lancets for the duration of the trial. They faxed or called in their BG logbooks every 2 wk to their HCPs. HCPs were asked to follow their usual standards of case.
**Rami 2006 ** [**141**]	Crossover RCT; *Country:* Austria; *Device:* Mobile telephone; *Media:* SMS	36 adolescents aged 10–19 y. Median age: Control = 16.2 y (range: 10.7–19); Intervention = 14.5 y (range: 12.9–19.3)	To evaluate whether VIEDIAB (a telemedical system and program) is feasible in adolescents with type 1 diabetes mellitus and if it would improve their glycaemic control.	Patients were instructed to measure at least four blood glucose values per day. They were advised to send their data every time they measured a blood glucose value or at least once daily. Once weekly, the patients with telemedicine support (TM phase) received either an automatically generated SMS (e.g., glycaemic control OK, nothing to be changed), or a personalized message with more specific advice (e.g., increase basal insulin in the morning by 2 IE). The analysis and the specific advice were conducted by the two diabetologists. Duration: 3 mo	In the control arm of the trial participants kept a paper diary. During this 3-mo time, there was no contact scheduled to or from the clinic.
**Schrezenmeir 2002 ** [**142**]	Parallel group RCT; *Country:* Germany; *Device:* Other handheld computer; *Media:* Custom software	50 people with diabetes. Mean age: Control = 36 y (SD: 3.5); Intervention = 33.3 y (SD: 3.6).	To investigate the effect of a pocket computer assistance in the intensive (meal related) insulin therapy.	The intervention was a specialized pocket computer that can register patient-entered data. An adaptation of the insulin doses to the current metabolic situation of the patient is achieved through continuous recalculations of individual patient data, such as insulin sensitivity, insulin-BG- and insulin-carbohydrate-equivalents. The intervention patients were asked to follow the dose recommendations given by the computer. Nevertheless, they had the opportunity to make their own decisions or ask their physician about the dose in case of doubts and critical situations. Duration: 6 mo	The control group patients were asked to document the following data: BG values, carbohydrate intake, sports, events, and injected insulin dosages.
**Vähätalo Ma 2004 ** [**46**]	Non-randomised parallel group trial; *Country:* Finland; *Device:* Mobile telephone; *Media:* SMS	203 patients in a Diabetes Outpatient Clinic. Mean age: Control = 43.1 y (SD: 13.6); Intervention = 42.8 y (SD: 11.4)	To assess transmission of glucose values by cellular phone in the treatment of type 1 diabetic patients.	Patients were advised to test their plasma glucose and send the results via the phone. The doctor checked all results and sent the patient a comment, even if no change of the therapy was needed. The patients were provided with 25 plasma glucose strips per week. The patients were encouraged to perform more measurements if the number of transferred measurements was too low according to clinical judgement. This was done by sending an SMS to the patient's phone. Duration: 1 y	Patients continued their normal visits to their diabetes physician at 3- to 4-mo intervals during the study.
**Yoo 2009 ** [**45**]	Parallel group RCT; *Country:* Korea; *Device:* Mobile telephone	123 patients with both type 2 diabetes and hypertension at least 1 y previously. Mean age: Control = 59.4 y (SD: 8.4); Intervention = 57 y (SD: 9.1).	To examine whether a UCDC system using both the internet and cellular phones could facilitate chronic disease self-management and improve multiple metabolic parameters in overweight patients with both type 2 diabetes and hypertension.	Patients in the intervention groups received a cellular phone with a modular blood glucose measuring device, strips, and lancets. They also received an automatic blood pressure monitoring device as well as body weight scales. First, the UCDC system sent out an alarm on the cellular phone to remind the participant to measure their blood glucose, blood pressure twice a day, and body weight once a day. The Anycheck device attached to their cellular phone conducted the glucose measurements and automatically sent the results to a central study database—participants immediately received messages of encouragement, reminders, and recommendations according to a pre-defined algorithm. The system automatically recorded participant's exercise time. Participants received information via SMS three times a day regarding healthy diet and exercise methods, along with general information about diabetes, hypertension, and obesity. Furthermore, physicians could follow participant's trends in blood glucose levels, blood pressure, and body weight changes, allowing them to send individualized recommendations to patients when needed. Duration: 3 mo	Patients in the control group visited their clinic according to their routine schedule and received the usual out-patient treatment from their physicians during the study period.

BG, blood glucose; BMI, body mass index; CARDS, computerized automated reminder diabetes system; HCP, health care provider; IBGMS, Internet-based glucose monitoring system; NICHE, novel interactive cell-phone technology for health enhancement; POIRO, patient oriented insulin regimen optimizer; RCT, randomized controlled trial; SD, standard deviation; UCDC, ubiquitous chronic disease care.

**Table 7:** Description of trials of asthma and chronic obstructive pulmonary disease interventions.

Study	Study Design, Country, Device, Media	Participants	Aims	Interventions	Comparators
**Liu 2007 ** [**143**]	Parallel group RCT; *Device:* Mobile telephone; *Media:* Application software	90 patients with chronic moderate to severe persistent asthma.	To determine if a mobile phone-based self-care system improves asthma control	In the intervention group, subjects operated a mobile phone with software to input these data on the phone through GPRS to a website for recording and analysis. The asthma severity assessed by PEFR, PEFR variability, and asthma symptoms was provided immediately on the mobile phone with according management suggestions. Duration: 6 mo	All subjects received asthma education, self-management plan, standard treatment, and were asked to measure daily PEFR and asthma symptoms but did not have access to the mobile device.
**Meltzer 2008 ** [**144**]	Crossover RCT; *Country:* USA; *Device:* Mobile telephone; *Media:* SMS	24 subjects ≥12 y of age with mild to moderate persistent asthma for at least 6 mo. Mean age: 36.17 y (Range: 16–61)	To assess use and patient acceptance of the VOCEL Mobile Diary in comparison with standard paper reporting methods in documenting the efficacy, safety, compliance, and convenience of inhaled ciclesonide in study participants.	Intervention subjects were issued a VOCEL Mobile Diary and instructed to complete this diary twice daily for a period of 2 wk. In addition, they received an electronic alert from the device at each scheduled time for diary entry throughout the evaluation period. Duration: 2 wk.	Control group used a paper diary.
**Mosnaim 2008 ** [**145**]	Parallel group RCT; *Country:* USA; *Device:* Portable media player; *Media:* MP3/audio	27 African American adolescents with an asthma diagnosis. Mean age: Control = 12.5 y (SD: 2.2); Intervention = 14.3 (SD: 2.4)	To evaluate the ability of the ADEPT for asthma and to increase asthma knowledge in our target population.	The intervention was celebrity (well-known musicians and athletes in the African-American community) asthma health messages delivered on a hand-held MP3 player (called Media Hub). The device is programmed so that the asthma content cannot be skipped to go to the next music track. Asthma health messages can be heard (1) when the Media Hub is first turned on, (2) before a selected stored music track is played, and (3) between two selected music tracks played consecutively. The network control system collects and stores information on the Media Hub user's choice of music tracks, asthma messages received, as well as amount of time spent with the device. Duration: 12 wk	The control group gets general health messages delivered on a hand-held MP3 player (called Media Hub) between music tracks. The network control system collects and stores information on the Media Hub user's choice of music tracks, general health messages received, as well as amount of time spent with the device.
**Ostojic 2005 ** [**47**]	Parallel group RCT; *Country:* Croatia; *Device:* Mobile telephone; *Media:* SMS	16 patients with moderate persistent asthma for at least 6 mo. Mean age: Control = 24.5 y (SD: 7.1); Intervention = 24.8 y (SD: 16.3)	To assess the feasibility and reliability of SMS as a tool of asthma monitoring and to ascertain its impact on control of asthma.	Patients in the SMS group were instructed to send their peak expiratory flow results daily via SMS. These then received weekly instructions by SMS from an asthma specialist on adjustments of therapy and recommended follow-up, based on the PEF values. When data received from a patient in the SMS group indicated significant asthma exacerbation, a request for an office visit was sent to the patient by SMS. Duration: 16 wk.	The control patients were to note PEF measurements, medication use, and symptoms in a paper diary, they were then seen in the office at the end of the study period, when their diary data were reviewed.
**Prabhakara 2010 ** [**79**]	Parallel group RCT; *Country:* Singapore; *Device:* Mobile telephone; *Media:* SMS	120 adults admitted for an acute exacerbation of asthma. Mean age: Control = 40 y; Intervention = 37 y	To evaluate the feasibility of using SMS for symptom monitoring through mobile phones from the user's perspective, and to evaluate patient compliance with SMS monitoring as measured by response rates.	The patients in the intervention group had SMS monitoring to assist with the management of their asthma control for 3 mo.	The patients in the control group were left to self manage their asthma for three mo.
**Strandbygaard 2010 ** [**146**]	Parallel group RCT; *Country:* Denmark; *Device:* Mobile telephone; *Media:* SMS	26 patients with asthma based on a clinical history and daily symptoms. Mean age: Control = 30.7 y; Intervention = 34.4 y.	To examine the impact of receiving a daily text message reminder on one's cell phone on the adherence to asthma treatment.	Intervention subjects received the following daily SMS reminder to take their asthma medication: “Remember to take your asthma medication morning and evening. From the Respiratory Unit” Duration: 8 wk.	Control subjects did not receive an SMS reminder

ADEPT, Adolescents' Disease Empowerment and Persistency Technology; GPRS, General Packer Radio Service; PEFR, peak expiratory flow rate; RCT, randomized controlled trial; SD, standard deviation.

**Table 8:** Description of trials targeting hypertension.

Study	Study Design, Country, Device, Media	Participants	Aims	Interventions	Comparators
**Carrasco 2008 ** [**48**]	Parallel group RCT; *Country:* Spain; *Device:* Mobile telephone *Media:* SMS	273 patients with hypertension. Mean Age: Control = 62.8 y (SD: 12.5); Intervention = 62.1 y (SD: 11.9).	To determine whether the patient–GP interaction based on the study protocol is sufficient to improve the control of hypertension achieved in primary care and without the intervention of any specialized setting. Also, to evaluate the improvement in hypertension during follow-up, the impact on patient quality of life and anxiety, and certain economic aspects concerning the viability of the telemedicine system in this context.	The intervention equipment in this project consists of a cellular phone with WAP-GPRS capability and a sphygmomanometer. During the 6-mo follow-up period, the intervention patients sent their mean sSBP and sDBP, based on three measurements made at 3-min intervals under fasting conditions in the morning and at night, four times a week, and their pulse rate and weight once a week. During each WAP session, they had the option of responding to a simple questionnaire. At some later moment, the GPs accessed the data sent by their patients via the web. According to his or her own criteria, the GP could send an SMS regarding any related issue to the patient's phone. Duration: 6 mo	The control patients followed the same SBPM protocol, with the exception that the results were recorded on paper in a data collection notebook; they sent no data to the computer system, and thus, had no interaction with their GPs.
**Marquez Contreras 2004 ** [**147**]	Cluster RCT; *Country:* Spain; *Device:* Mobile telephone; *Media:* SMS	104 patients with uncontrolled hypertension treated with monotherapy, willing to initiate anti-hypertension treatment with combined anti-hypertensive drugs. Mean age: Control = 59.43 y (SD: 10.94); Intervention = 56.26 y (SD: 10.22).	To analyze the effect of an intervention to provide information with mobile phone text messages to patients with hypertension.	SMS messages were sent twice a week between 11 am and 2 pm (not on weekends) randomly, during the 6 mo of follow-up. The objective of the messages: give information on hypertension, enhance compliance, suggest hygienic dietary habits, and remind the patients about taking their medication during the 6 mo of follow-up. Duration: 6 mo	Controls received usual care.

GP, general practitioner; RCT, randomized controlled trial; SBPM, self-blood pressure monitoring; SD, standard deviation; sDBP, self-measured diastolic blood pressure; sSBP, self-measured systolic blood pressure; WAP, wireless application protocol.

**Table 9:** Description of trials of psychological support interventions.

Study	Study Design, Country, Device, Media	Participants	Aims	Interventions	Comparators
**Mosso 2009 ** [**148**]	Parallel group RCT; *Country:* Mexico; *Device:* Mobile telephone; *Media:* MP4/video	21 patients who underwent an ambulatory surgical operation. Mean age: Control = 55.4 y (SD 21.2); Intervention = 44.4 y (SD 13.2). Males: Control = 40%; Intervention = 72.73%	To verify the effectiveness of VR in reducing anxiety in patients undergoing ambulatory operations under local or regional anaesthesia.	The intervention was a pre-recorded video of the Green Valley, a very relaxing environment showing a mountain landscape around a calm lake presented together with relaxing music and soft sounds. Patients have the impression of walking around the lake, they can observe nature, and virtually sit on a comfortable deck chair. Patients wore the head mounted display and the headphones connected to the mobile few minutes before the anaesthetic injection. Duration: 90 min	No distraction during the operation was provided.
**Riva 2007 ** [**149**]	Parallel group RCT; *Country:* Italy; *Device:* Mobile telephone, Portable media player; *Media:* MP3/audio, MP4/video	30 female university students, performing an exam within a week.	To determine whether portable audio/video support helps manage exam stress.	MP3 intervention: audio only narrative. Mobile intervention: audio and video narrative.	Usual behaviour prior to exams.
**Patel 2006 ** [**150**]	Parallel group RCT; *Country:* USA; *Device:* Handheld video game console; *Media:*Application software	74 children aged 4–12 y undergoing general anaesthesia for elective surgery in an ambulatory surgery centre. Mean age: Control = 6.6 y (SD 0.4); Intervention = 7.0 y (SD 0.4). Males: Control = 61%; Intervention = 63%.	To evaluate the efficacy of an interactive distraction, a handheld VG in reducing pre-operative anxiety in children.	Children and parents remained in private cubicles in the ambulatory surgery unit before transfer into the OR. Children were then given a VG. At least 20 min after the intervention, parents and patient were escorted into the OR where standard pre-anaesthesia procedures were carried out prior to mask induction of general anaesthesia. Patients were allowed to play on the VG through the induction of the mask.	Children and parents remained in private cubicles in the ambulatory surgery unit before transfer into the OR. At least 20 min later, parents and patient were escorted into the OR where standard pre-anaesthesia procedures were carried out prior to mask induction of general anaesthesia.
**Riva 2006 ** [**151**]	Parallel group RCT; *Country:* Italy; *Device:* Mobile telephone; *Media:* MP3/audio, MP4/video	33 commuters on the Milano-Saronno train line aged between 20–25 y.	To assess if mobile narratives may be used to improve the mood state in their users.	Intervention group 1: mobile narratives: this group experienced four mobile narratives based on a trip in a desert tropical beach. Intervention group 2: new age videos: this group experienced four commercial videos with new age music. Duration: 2 d	No treatment was given.
**Pijnenborg 2010 ** [**152**]	Quasi-random controlled trail; *Country:* The Netherlands; *Device:* Mobile telephone; *Media:* SMS	62 patients that received care in the Department of Psychotic Disorders with observed limitations in goal directed behaviour in daily life. Mean age: 28.8 y	To evaluate the efficacy of SMS text messages in prompting behaviours.	Patients were prompted with SMS text messages to improve their everyday functioning. The intervention was tailored to individual needs by encouraging patients to choose their own goals. For each patient, a schedule of SMS text messages was developed and entered into a website. For each goal, two prompts were sent. The first was sent an hour before the goal behaviour should take place, to enable patients to fit the target action into their current schedule. A second prompt was provided 10 min before goal behaviour was due, so patients could then initiate relevant actions. Duration: 7 wk	No SMS reminders were sent prior to goal behaviour.
**Grassi 2009 ** [**153**]	Parallel group RCT; *Country:* Italy; *Device:* Mobile telephone, Portable media player; *Media:* MP3/audio, MP4/video	120 university commuters aged 20 to 25 y.	To determine if a narrative based on relaxation exercises and associated with video content could be more effective in positive emotion induction, significantly decreasing anxiety, than a video only condition.	Each of the intervention groups had 4 sessions over 2 consecutive days (morning and evening journey). Each session lasted 10 min. **Int 1:** presented with video content of a virtual island associated with audio/relaxation content. **Int 2:** video content of a virtual island (no audio). **Int 3:** audio relaxation content only. Duration: 2 d	The control group filled out questionnaires without having received any intervention.
**Gorini 2010 ** [**154**]	Parallel group RCT; *Country:* Italy; *Device:* Mobile telephone; *Media:* Application software	21 consecutive patients aged 18–50 y with a diagnosis of GAD (as determined by the DSM-IV-TR criteria).	To assess the impact of a virtual reality on computer and mobile phone with or without biofeedback on patients with GAD.	Patients in the intervention groups received virtual reality treatments in therapy sessions plus the same treatment at home, on their mobile phones, with and without biofeedback (biofeedback in therapy session only). These treatments involved the patient exploring a beautiful tropical island following a pre-defined path leading to different relaxing areas: campfire, beach, and waterfall. Each experience was accompanied by an audio narrative based on muscle relaxation and/or autogenic techniques.	Control participants did not receive any VR treatment, by mobile phone or otherwise.

DSM-IV-TR, Diagnostic and Statistical Manual of Mental Disorders; GAD, generalised anxiety disorder; OR, operating room; RCT, randomized controlled trial; SD, standard deviation; VG, video game; VR, virtual reality.

**Table 10:** Description of trials of other disease management interventions.

Interventions	Study	Study Design, Country, Device, Media	Participants	Aims	Interventions	Comparators
**Other disease management interventions**	**Devito Dabbs 2009 ** [**38**]	Parallel group RCT; *Country:* USA; *Device:* PDA; *Media:* Custom software	30 patients post-lung transplant, post-ICU recovery period. Mean age: Control = 57 y (SE: 11); Intervention = 55 y (SE: 12.7); 60% male	To evaluate the efficacy of Pocket Path (personal assistant for tracking health) for promoting self-care agency, self-care behaviours and health-related quality of life in the early months after lung transplantation.	In addition to standard care, the intervention group were given the Pocket PATH device and a user manual, along with training. Patients post-lung transplant entered data on daily health parameters using the device, review data trends by using the screens and graphs, and follow feedback instructions regarding reporting changes to their transplant coordinator. Duration: 2 mo.	The control patients used standard paper/pencil logs to record data.
	**Kearney 2009 ** [**155**]	Parallel group RCT; *Country:* UK; *Device:* Mobile telephone; *Media:* SMS	110 patients diagnosed with lung, breast, or colorectal cancer commencing a new course of chemotherapy treatment or receiving outpatient chemotherapy. Mean age: Control = 56.9 y (SD: 10.5); Intervention = 56 y (SD: 10.5)	To evaluate the impact of a mobile phone-based remote monitoring ASyMS on the incidence, severity and distress of 6 chemotherapy-related symptoms (nausea, vomiting, fatigue, mucositis, hand-foot syndrome, and diarrhoea).	Intervention group used ASyMS throughout 4 cycles of chemotherapy. On days 1 to 14, in the morning, evening and at any time they felt unwell, they completed a symptom questionnaire. This information was sent to the study server. Patients immediately received feedback on the phone comprising tailored self-care advice directly related to the severity of the symptoms they had reported. Duration: 12–16 wk.	Standard care following guidelines and procedures related to the monitoring and reporting of chemotherapy-related toxicity in their local area. This included written information and verbal information from the nurses.
	**Scherr 2009 ** [**156**]	Parallel group RCT; *Country:* Austria; *Device:* Mobile telephone; *Media:* SMS	108 patients with acute worsening of heart failure. Mean age: Control = 67 y (IR: 61–72); Intervention = 65 y (IR: 62–72).	To evaluate the impact of home-based telemonitoring using Internet and mobile phone technology on the outcome of heart failure patients after an episode of acute decompensation.	Tele group patients were asked to measure vital parameters (blood pressure, heart rate, body weight) on a daily basis at the same time. Patients were advised to enter these values as well as their dosage of heart failure medication into the mobile phone's Internet browser and send them to the monitoring centre. Study physicians had access to a secure website providing both numerical and graphical depiction of data for each patient. Whenever necessary, study physicians could contact patients using the mobile phone. If transmitted values went outside individually adjustable borders, study physicians were sent an email alert. Additionally, an email alert was generated if a patient's body weight increased or decreased more than 2 kg in 2 d. After receiving an alert, study physicians could contact the patient directly via the mobile phone to confirm the parameters and, if appropriate, could ask the patient to adjust his or her medication. Duration: 6 mo.	Usual pharmacological treatment care. There was no planned interaction between study site and patients in the control group.
	**Walker 2004 ** [**157**]	Parallel group RCT; *Country:* Canada; *Device:* Other handheld computer; *Media:* Application software	40 patients with severe haemophilia (factor VIII or FIX<1%) participating in a home care infusion programme. Mean age: Control = 26 y (IQR: 13–44); Intervention = 25 (IQR: 15–42).	To compare hand-held computers with paper diaries in terms of data recording and transfer, time interval between individual infusions and the receipt of data; the number of reminder calls to retrieve data; the accuracy of data; and comprehensiveness of data.	Data capture and reporting was carried out using a hand-held computer. Essential data required from both groups included: date of infusion, purpose, site of bleeding (if any), factor concentrate lot number(s), and number of vials. Data for each infusion were transmitted at the touch of a button to a server at the haemophilia clinic. The server then transmitted an e-mail message to the clinic coordinator, with a special alert for high-risk bleeds. Duration: 6 mo	The control group reported on infusion data by paper diary. The paper diaries, designed by the Canadian Association of Nurses in Hemophilia Care, are widely used across Canada. Each page on the paper diary was pressure-sensitive and duplicated with one copy to be retained by the patient.
	**Culley 2010 ** [**89**]	Non-randomised parallel group trial; *Country:* UK, Scotland; *Device:* Mobile telephone; *Media:* SMS	22 participants from two post-acute brain-injury rehabilitation centres that offer goal-oriented approaches. Mean age: 36 y	To examine the effectiveness of sending SMS messages to participants with an acquired brain injury as a means of improving recall for their rehabilitation goals.	Each patient had at least 6 goals that they were working toward in the rehabilitation facility. Participants were involved in setting cue words for each of the goals before the study began. Three goals per participant were randomly selected to be allocated to the “text” condition. Texts were set to be delivered to the participant's mobile phone three times per day (9.30 am, 3 pm, 7 pm) for 14 d. The first text message was received following completion of the baseline assessment. Duration: 14 d	For three of the goals selected for each of the participants, no text message reminder was sent.
	**Choi 2009 ** [**158**]	Non-randomised parallel group trial; *Country:* Korea; *Device:* Mobile telephone; *Media:* MP4/video	188 patients with lacerations or sprains who were discharged from the emergency department.	To assess the effectiveness of MDIVs in improving the comprehension of discharge instructions among patients with lacerations or sprains. To assess the convenience of and satisfaction with MDIVs. Mean age: Control = 36 y (SD: 8.1); Intervention = 38.1 y (SD: 8). Male: Control 23%; Intervention 27%.	MDIVs, defined as discharge instructions on video, are watched by adult patients or the parents/guardians of children using their mobile phones. The MDIV was sent to the adult patients or to the parents/guardians through their mobile phone service provider's multimedia message mail service. Duration: 1 min, 15 s.	The printed discharge instructions were directly given to the adult patients or to the parents/guardians at discharge.
**Ante-natal care**	**Jareethum 2008 ** [**49**]	Parallel group RCT; *Country:* Thailand; *Device:* Mobile telephone; *Media:* SMS	68 pregnant women who received antenatal care and planned to deliver at the study centre. Mean age: Control = 29.57 y (SD 6.1), Intervention = 28.72 y (SD 4.9).	To compare the satisfaction levels of antenatal care between healthy pregnant women who received SMS via mobile phone for prenatal support and those who did not. Also compared the confidence, anxiety levels and pregnancy outcomes.	This group received 2 SMS messages per week from the 28 wk of gestation until delivery. Both groups had the same antenatal and perinatal care. The SMS messages contained information and warnings relating to abnormal symptoms, which would require that, they consult the doctor. The SMS messages were appropriate to the women's gestational age. All participants received a phone call at 32 wk of gestation. The aim of the phone call was to check that they still contacted the Siriraj antenatal clinic and confirmed that the intervention group could receive and understand the SMS messages.	The control group received no treatment other than standard antenatal care and a phone call at 32 wk.

ASyMS, advanced symptom management system; ICU, intensive care unit; IQR interquartile range, MDIV, mobile discharge instruction video; RCT, randomized controlled trial; SD, standard deviation.

One disease management trial was conducted in a low-income country (Kenya), and two were conducted in middle-income countries (Mexico, Thailand). All other trials were conducted in high-income countries.

### Interventions

We describe the interventions according to the device (mobile phone, PDA, etc.) and functions used (SMS, multi-media messaging service MMS, software application, video). Behaviour change interventions are described according to the study authors' descriptions of the behavioural change theory underpinning the intervention and behaviour change techniques employed. Further details of each intervention, as described by authors, are provided in Tables 1–10.

#### Health behaviour change

Sixteen of the health behaviour change interventions used mobile phones, of which 13 used the SMS function, one used the MP3 function, one used the MP4 function, and one used the telephone function. One intervention was delivered using a PDA phone using application software, SMS, and telephone functions. Five interventions were delivered using PDAs using application software, two used hand held computers employing application software or MP4/video functions, one used the audio functions provided by a MP3 audio players.

Of the 26 studies, seven reported using behavioural theories to underpin their intervention. The maximum number of techniques reported by any intervention description was 18 [21],[22],[36] and the median number was six. The most commonly used behaviour change techniques were the following: provide feedback on performance (13 interventions), goal setting (12 interventions), provide information on the consequences of behaviour generally (11 interventions), tailoring (11 interventions), prompt self-monitoring of behaviour (10 interventions), and identify barriers to behaviour/problem solving/identify ways of overcoming barriers (eight interventions). The behavioural theories and behaviour change techniques reported in the description of interventions in the trial reports are found in Tables 11 and 12.

**Table 11:** Techniques employed in behaviour change interventions.

Behaviour Change Technique^a^	Number of Studies^b^	Behaviour Change Technique^a^	Number of Studies^b^
Provide feedback on performance	13 [36,105–107,109,112,115–122]	Facilitate social comparison	1 [125]
Provide information on consequences of behaviour in general	11 [36,86,105–107,112–113,119,122–123,125,172]	Model/Demonstrate the behaviour	1 [125]
Tailoring	11 [36,105–107,112,114–119,127]	Provide information on where and when to perform the behaviour	1 [119]
Goal setting (behaviour)	12 [83,86,109,111–114,119–120,122–123],	Prompt practice	1 [113]
Prompt self-monitoring of behaviour	10 [36,105–106,111–112,114,116–117,120–121,123]	Prompt review of behavioural goals	1 [113]
Barrier identification/problem solving	8 [36,105–106,109,112,114,119,122,172]	Provide rewards contingent on successful behaviour	1 [105–106]
Action planning	5 [36,86,105–106,112–113]	Agree behavioural contract	0
Goal setting (outcome)	5 [36,105–106,112,115,118]	Emotional control training	0
Prompt self-monitoring of behavioural outcome	5 [105,109,115,118–119,122]	Fear arousal	0
Provide instruction on how to perform the behaviour	5 [36,105–106,109,112,127]	General communication skills training	0
Use of follow-up prompts	5 [36,86,105–106,113,172]	Motivational interviewing	0
Plan social support/social change	3 [36,105–106,172]	Prompt anticipated regret	0
Relapse prevention/coping planning	3 [36,105–106,172]	Prompting generalisation of a target behaviour	0
Teach to use prompts/cues	3 [86,111,119]	Prompt identification as role model/position advocate	0
Environmental restructuring	2 [36,105–106]	Prompt self talk	0
General planning	2 [36,105–106]	Prompt use of imagery	0
Prompting focus on past success	2 [36,105–106]	Provide rewards contingent on effort or progress towards behaviour	0
Provide information on consequences of behaviour to the individual	2 [36,105–106]	Shaping	0
Provide information about others' approval	2 [36,105–106]	Stress management	0
Provide normative information about others' behaviour	2 [123,125]	Stimulate anticipation of future rewards	0
Prompt review of outcome goals	2 [118–119]	Time management	0
Set graded tasks	2 [36,105–106]		

a Using taxonomy of behaviour change techniques.

b Total may add up to more than 26 as some interventions used more than one behaviour change technique.

**Table 12:** Frequency of reported use of behaviour change theories.

Behaviour Change Theory	Number of Studies
Social cognitive theory	4 [109,114,118,123]
Elaboration likelihood theory	1 [123]
Implementation theory/automotive model	1 [113]
Protection motivation theory	1 [86]
Social learning theory	1 [114]
Transtheoretical model	1 [107]

#### Disease management

Forty-two of the 49 disease management interventions were delivered by mobile phone or PDA mobile phone with 27 using the SMS function, three using MP4 video functions, four using MP3 audio and MP4 video functions, six using application software, one using WAP for data transfer, and one using telephone functions. Two interventions were delivered via PDA, three used hand held computers, one used a video console all of which employed application software. One used a portable media player/MP3 employing audio functions.

### Outcomes

#### Health behaviour change

The health behaviour change trials reported between one and 23 outcomes. Primary outcomes were as follows: Four trials reported four different medical outcomes such as HBA1C (*n* = 1), blood pressure (*n* = 1), and deaths (*n* = 1). Fourteen trials reported seven different anthropometric outcomes (e.g., height, weight). Eight trials reported four different objective measures of behaviour.

Secondary outcomes were as follows: Thirteen trials reported 38 different self-reported behaviour outcomes and four trials reported seven different knowledge, cognitive, or other mediator outcomes.

#### Disease management

The disease management trials reported between one and 25 outcomes. Primary outcomes were as follows: Seventeen trials measured 28 different medical outcomes. Three trials measured four different anthropometric outcomes. Secondary outcomes were as follows: Thirty trials reported 125 different medical, disease control, or medical process outcomes such as hospitalization, cardiopulmonary resuscitation (CPR) skills, adherence to treatment, and self-monitoring of disease. Eleven cognitive mediator or other mediator outcomes were reported (such as knowledge and attitudes).

### Study Quality

#### Health behaviour change

**Figure 2:**
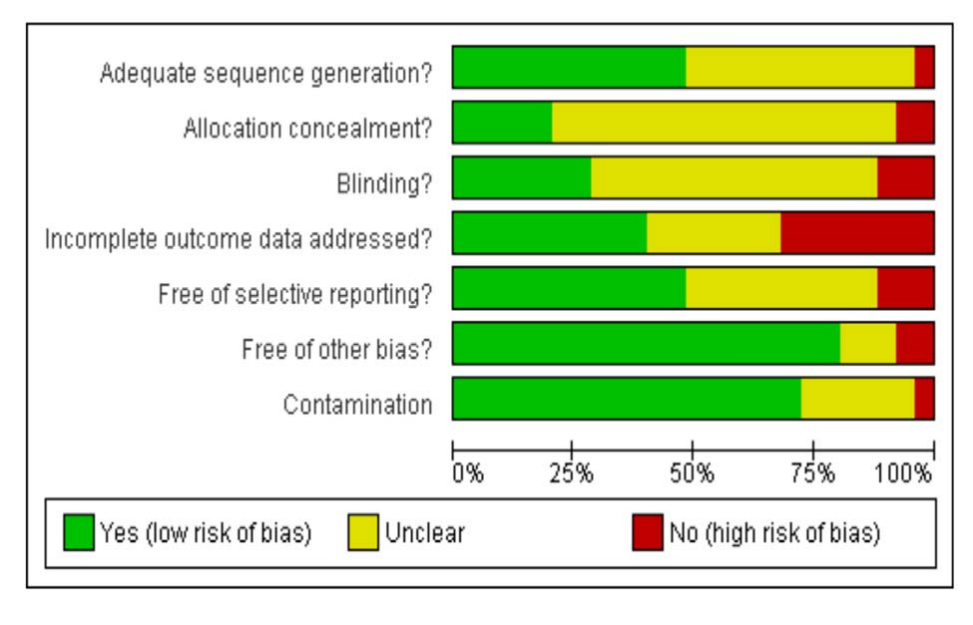
Cochrane summary risk of bias for health behaviour change trials.

The assessment of **s**tudy quality is reported in Table 13 and the Cochrane risk of bias summary is reported in Figure 2. Two trials were at low risk of bias for all quality criteria [21],[36]. No evidence of publication bias was demonstrated on visual or statistical examination of the funnel plots.

**Table 13:** Methodological quality summary for health behaviour change interventions.

Trial	Sequence Generation	Allocation Concealment	Blinding	Incomplete Outcome Data	Selective Outcome Reporting Bias	Contamination	Other Bias
**Physical activity and diet**							
Agras (1990) [125]	L	U	U	U	L	L	L
Burke 2010) [40]	U	U	U	U	L	U	L
Haapala (2009) [121]	U	U	U	H	U	L	L
Patrick (2009) [122]	U	U	H	H	U	L	L
Shapiro (2008) [123]	L	U	U	H	U	L	L
Shay (2009) [124]	U	U	U	U	U	L	L
Turner-Mcgrievy (2009) [126]	U	U	U	L	L	L	H
**Physical activity**							
King (2008) [115]	U	U	U	L	U	U	L
Liu (2008) [116]	L	U	U	U	H	L	L
Newton (2009 [42])	U	U	L	L	U	L	U
Nguyen (2009) [117]	U	L	L	L	L	L	L
Prestwich (2009) [96]	L	U	U	U	H	U	L
Prestwich (2010) [118]	L	L	L	L	U	L	L
Sirriyeh 2010 [10]	L	L	L	L	L	U	U
**Diet**							
Atienza (2008) [119]	L	U	U	H	L	L	L
Beasley (2008) [41]	L	H	H	H	L	H	H
Ellrott (2005) [120]	U	U	U	U	U	U	U
**Smoking**							
Free (2009) [112]	L	L	L	L	L	L	L
Free (2011) [36]	L	L	L	L	L	L	L
Haug (2009) [113]	L	U	L	H	L	L	L
Rodgers (2005) [39]	L	L	L	H	L	L	L
Vidrine (2006) [114]	L	U	U	L	H	L	L
**Sexual Health**							
Delamere (2006) [127]	U	U	U	H	U	U	L
Jones (2008) [128]	H	H	H	L	L	L	L
Lim 2007 [62]	U	U	U	U	U	L	L
**Alcohol**							
Weitzel (2007) [130]	U	U	U	L	L	L	L

L, low; H, high; U, unclear; N, no; Y, yes.

#### Disease management

**Figure 3:**
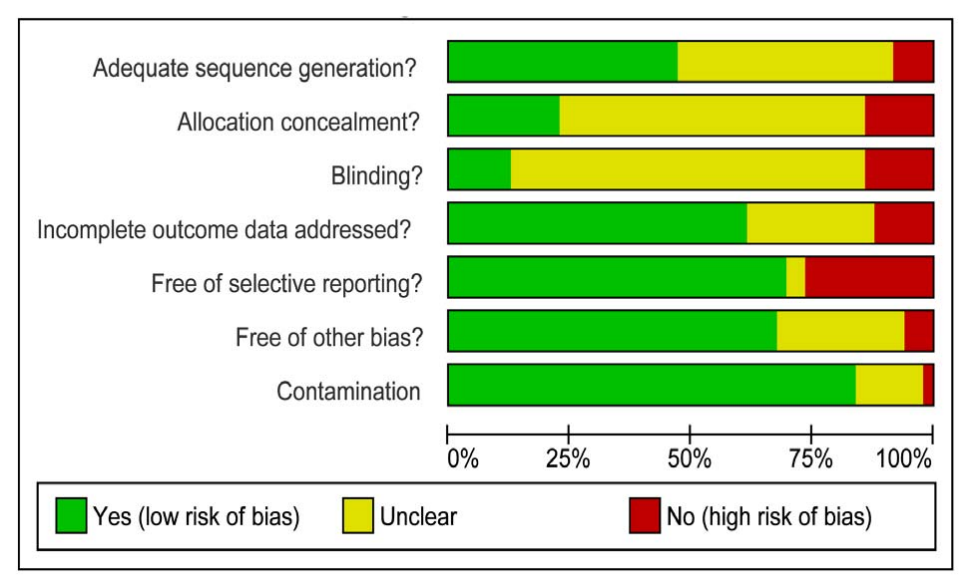
Cochrane risk of bias summary for emergency care by lay people and self management of diseases.

The assessment of **s**tudy quality is reported in Table 14 and the Cochrane risk of bias summary is reported in Figure 3. Two trials were low risk of bias in all areas assessed [37],[38]. No evidence of publication bias was demonstrated on visual or statistical examination of the funnel plots.

**Table 14:** Cochrane risk of bias summary for emergency care delivered by lay people and self-management of diseases.

Trial	Sequence Generation	Allocation Concealment	Blinding	Incomplete Outcome Data	Selective Outcome Reporting Bias	Contamination	Other Bias
**Acute disease**							
Bolle 2009 [133]	U	H	U	L	L	L	L
Choa 2009 [131]	L	U	U	L	L	L	L
Choa 2008 [159]	L	U	L	L	L	L	L
Choi 2009 [134]	H	H	L	L	L	L	L
Ertl 2007 [162]	L	U	U	L	L	L	L
Merchant 2010 [97]	L	U	U	L	L	U	L
Yang 2009 [132]	U	U	U	L	L	L	L
Zanner 2007 [160]	U	U	U	L	L	L	U
**Chronic disease**							
Benhamou 2007 [143]	L	L	U	L	H	L	L
Carrasco 2008 [48]	L	U	U	H	L	L	H
Cho 2009 [134]	U	U	U	L	H	H	U
Devito Dabbs 2009 [38]	L	L	L	L	L	L	L
Faridi 2008 [44]	U	U	U	U	L	L	L
Franklin 2006 [134]	L	L	U	L	H	L	L
Hanauer 2009 [136]	U	U	U	H	L	L	U
Holman 1996 [137]	L	U	U	L	L	U	L
Istepanian 2009 [138]	L	U	U	H	L	L	U
Kearney 2009 [155]	L	U	U	H	L	L	U
Kim 2007 [139]	U	U	U	U	H	L	L
Liu 2007 [143]	U	U	U	U	H	U	U
Marquez Contreras 2004 [147]	L	U	U	H	L	L	L
Meltzer 2008 [144]	U	U	U	U	L	L	U
Mosnaim 2008 [145]	L	U	L	L	L	U	L
Ostojic 2005 [47]	L	U	U	L	L	L	L
Quinn 2008 [140]	U	U	H	U	L	L	L
Prabhakaran 2010 [79]	U	L	U	L	L	L	U
Rami 2006 [141]	U	U	U	L	L	L	L
Scherr 2009 [156]	L	U	H	L	H	L	H
Schrezenmeir 2002 [142]	U	L	U	U	H	L	L
Strandbygaard 2010 [146]	L	L	H	U	H	L	L
Vähätalo 2004 [46]	U	H	U	U	L	L	U
Walker 2004 [157]	L	H	H	L	L	L	L
Yoo2009 [45]	U	U	H	L	L	L	L
**Medication adherence**							
Armstrong 2009 [165]	L	L	U	L	L	L	L
Cocosila 2009 [163]	L	L	U	L	L	L	H
Lester 2010 [37]	L	L	L	L	L	L	L
Ollivier 2009 [104]	L	U	U	L	U	U	L
Villella 2004 [139]	H	H	U	L	L	L	L
Yang 2008 [161]	U	U	U	U	U	U	U
**Treatment programmes**							
Gorini 2010 [154]	H	H	H	U	H	L	U
Jareethum 2008 [49]	L	U	U	L	L	L	L
**Other**							
Culley 2010 [89]	U	L	L	L	L	L	U
Grassi 2009 [153]	U	U	U	L	H	L	U
Mosso 2009 [148]	U	U	U	U	L	L	L
Patel 2006 [150]	L	L	U	L	L	L	L
Pijnenborg 2010 [152]	H	H	H	H	H	U	L
Riva 2007 [149]	U	U	U	U	H	L	L
Riva 2006 [151]	U	U	U	U	H	L	L

L, low; H, high; U, unclear; N, no; Y, yes.

### Effects

For each type of intervention we report primary outcomes and a summary of secondary outcomes. Full details of the secondary outcomes are available in Tables 15–17.

**Table 15:** Secondary outcomes health behaviour change interventions.

Clinical area	Trial	Intervention	Outcome	MD	LCI	UCI
**Safe sex**	Delamere 2006 [127]	Weekly SMS for 3 mo	Change of sexual partner	3.66	0.95	14.05
	Delamere 2006	Weekly SMS for 3 mo	Unprotected sexual intercourse	2.03	0.47	8.81
**Smoking**	Free 2011 [36]	5 SMS/day post quit date versus no SMS	Point prevalence of smoking cessation - 6 wk	2.44	2.25	2.65
	Free 2011	5 SMS/day post quit date versus no SMS	Point prevalence of smoking cessation - 6 mo	1.32	1.19	1.47
	Free 2011	5 SMS/day post quit date versus no SMS	28-d continuous abstinence - 6 mo	1.47	1.30	1.66
	Free 2009 [112]	5 SMS/day post quit date versus no SMS	Point prevalence of smoking cessation (no smoking in last week, self-reported) - 6 mo	0.76	0.41	1.41
	Free 2009	5 SMS/day post quit date versus no SMS	Currently not smoking (at follow-up - self-reported)- 6 wk	2.08	1.11	3.89
	Free 2009	5 SMS/day post quit date versus no SMS	28-d continuous abstinence - 6 mo	0.79	0.41	1.52
	Rodgers 2005 [39]	5 SMS/day versus no SMS	Point prevalence of smoking cessation (1 wk, self-reported) - 6 wk	2.20	1.79	2.70
	Rodgers 2005	5 SMS/day versus no SMS	Point prevalence of smoking cessation (1 wk, self-reported) - 26 wk	1.07	0.91	1.26
	Rodgers 2005	5 SMS/day versus no SMS	24 wk near-abstinence (<4 lapses of <3 cigarettes) - 26 wk	1.64	1.12	2.42
	Rodgers 2005	5 SMS/day versus no SMS	24 wk complete abstinence - 26 wk	1.50	0.92	2.44
	Vidrine 2006 [114]	Counselling sessions on mobile phone versus usual care	24 hour abstinence (at 3-mo follow-up)	1.31	1.08	1.59
	Vidrine 2006	Counselling sessions on mobile phone versus usual care	Longest period of continuous abstinence (days)	14.20	2.92	25.48
	Haug 2009 [113] - 1 Sms	1 SMS/wk with tailored feedback versus weekly SMS	Cigarettes per day - 3 mo	0.70	−1.55	2.95
	Haug 2009 - 3 Sms	3 SMS/wk with tailored feedback versus weekly SMS	Cigarettes per day - 3 mo	0.20	−1.91	2.31
**Energy expenditure**	Newton 2009 [42]	Motivational SMS versus standard care	Exercise - change (minutes per week)	9.90	−20.70	40.50
	Prestwich 2010 [96]	SMS goal reminder versus no SMS	Exercise - days per week ≥30 min	0.53	−0.27	1.33
	Prestwich 2009 [118]	SMS plan reminder versus no SMS	Exercise - days per week ≥30 min	0.85	−47.81	49.51
	Prestwich 2010	SMS goal reminder versus no SMS	Physical activity - walking - days per week ≥30 min	0.81	−0.01	1.63
	Prestwich 2009	SMS plan reminder versus no SMS	Physical activity - walking - days per week ≥30 min	0.81	0.02	1.60
	King 2008 [115]	Motivational PDA software versus standard written guidelines	Exercise (minutes per week)	166.60	1.60	331.60
	King 2008	Motivational PDA software versus standard written guidelines	Energy expenditure (Kcal per kg per week)	9.20	−1.40	19.80
**Diet and physical activity**	Shapiro 2008 [123]	SMS versus no SMS	Exercise (minutes per day)	23.20	−96.34	142.74
	Shapiro 2008	SMS versus no SMS	TV/computer screen usage (minutes per day)	−31.20	−89.01	26.61
	Turner-Mcgrievy 2009 [126]	Weight-loss podcast	Inactivity - sitting (hours per day)	−1.00	−3.34	1.34
	Turner-Mcgrievy 2009	Weight-loss podcast	Physical activity - moderate (days per week)	0.30	−0.64	1.24
	Turner-Mcgrievy 2009	Weight-loss podcast	Physical activity - vigorous (days per week)	0.70	−0.08	1.48
	Turner-Mcgrievy 2009	Weight-loss podcast	Physical activity - walking (days per week)	0.10	−0.91	1.11
**Dietary outcomes: secondary**	Shapiro 2008	SMS versus no SMS	Sugar sweetened beverage servings per day	0.30	−0.23	0.83
	Haapala 2009	SMS versus no SMS	Energy-dense food consumption score - SMD	0.00	−0.43	0.43
	Turner-Mcgrievy 2009	Weight-loss podcast	Fatty food consumption score - SMD	−0.20	−0.65	0.25
	Turner-Mcgrievy 2009	Weight-loss podcast	Fruit consumption score – SMD	0.00	−0.45	0.45
	Turner-Mcgrievy 2009	Weight-loss podcast	Vegetable consumption score -SMD	0.00	−0.45	0.45
**Behaviour change mediators: secondary**	Turner-Mcgrievy 2009	Weight-loss podcast	Adherence - number of podcasts downloaded	0.90	−2.59	4.39
	Haapala 2009	SMS versus no SMS	Self efficacy in dieting score - SMD	−0.13	−0.56	0.31
	Shay 2009 [124]	SMS versus no SMS	Self efficacy for weight management score - SMD	0.05	−0.70	0.81
	Turner-Mcgrievy 2009	Weight-loss podcast versus standard weight-loss podcast	Cognitive load score - SMD	1.14	0.66	1.62
	Turner-Mcgrievy 2009	Weight-loss podcast versus	Weight loss knowledge score - SMD	0.99	0.52	1.47
	Turner-Mcgrievy 2009	Weight-loss podcast versus Standard weight-loss podcast	1.20	0.71	1.69	—
	Turner-Mcgrievy 2009	Weight-loss podcast versus standard weight-loss podcast	0.90	0.43	1.37	—
**Diet: total energy intake**	Ellrott 2005 [120]	Nutrition-related information software on handheld computer	Total daily calorie intake	−132.66	−302.32	37.00
**Diet: Energy intake from food groups: secondary**	Ellrott 2005	Nutrition-related information software on handheld computer	Percent of energy intake from fat	−2.00	−4.77	0.77
	Ellrott 2005	Nutrition-related information software on handheld computer	Percent of energy intake from carbohydrate	1.82	−1.39	5.03
	Ellrott 2005	Nutrition-related information software on handheld computer	Percent of energy intake from protein	−0.14	−1.63	1.35
**Fruit and vegetable intake: secondary**	Ellrott 2005	Nutrition-related information software on handheld computer	Fruit and vegetable consumption - grams per day	29.21	−45.64	104.06
	Ellrott 2005	Nutrition-related information software on handheld computer	Fruit and vegetable consumption - servings per day	0.30	−0.28	0.88
**Protein intake: secondary**	Ellrott 2005	Nutrition-related information software on handheld computer	Protein consumption - grams per day	−5.30	−12.89	2.29
**Carbohydrate intake: secondary**	Ellrott 2005	Nutrition-related information software on handheld computer	Carbohydrate consumption - grams per day	−10.23	−31.09	10.63
**Fibre intake: secondary**	Ellrott 2005	Nutrition-related information software on handheld computer	Dietary fibre consumption - grams per day	−0.88	−4.82	3.06
**Fat intake: secondary**	Ellrott 2005	Nutrition-related information software on handheld computer	Cholesterol consumption- milligrams per day	−30.77	−73.40	11.86
	Ellrott 2005	Nutrition-related information software on handheld computer	Fat consumption - grams per day	−8.09	−16.98	0.80
**Vitamin C intake: secondary**	Ellrott 2006	Nutrition-related information software on handheld computer	Vitamin C consumption - milligrams per day	2.32	−24.06	28.70
	Jones 2008 [128]	Video based on sex-script theory viewed on handheld computer versus video on careers in healthcare	Self-efficacy for negotiating contraceptive use - score	−2.66	−6.62	1.30

**Table 16:** Secondary outcomes for emergencies.

Area	Trial	Intervention	Outcome	RR/MD	LCI	UCI
Cardiopulmonary resuscitation	Merchant 2010 [97]	Pre-recorded message versus no assistance	Compression: adequate rate	36.00	9.14	141.73
	Choa 2008 [159]	Animation-assisted CPR instructions on mobile phone versus no assistance	Compression: adequate rate	1.25	0.52	3.00
	Yang 2009 [132]	Interactive voice and video CPR instructions versus voice only	Compression: proportion done with sufficient rate	1.46	0.86	2.49
	Choa 2009 [131]	Animation-assisted CPR instructions on mobile phone versus no assistance	Check response/call for help	0.30	0.26	0.34
	Bolle 2009 [133]	CPR instructions via video call versus audio call	Number where there were no compressions or ventilations	0.13	0.01	2.38
	Bolle 2009	CPR instructions via video call versus audio call	Number where no ventilations	0.30	0.03	2.70
	Zanner 2007 [160]	M-AID CPR application on mobile phone versus no support	Proportion: check consciousness on unresponsive patient	0.74	0.45	1.24
	Zanner 2007	M-AID CPR application on mobile phone versus no support	Proportion: check for breathing	2.11	1.29	3.45
	Zanner 2007	M-AID CPR application on mobile phone versus no support	Proportion: chest compressions	0.66	0.40	1.09
	Zanner 2007	M-AID CPR application on mobile phone versus no support	Proportion: emergency phone call	1.08	1.00	1.17
	Zanner 2007	M-AID CPR application on mobile phone versus no support	Proportion: improved survival	0.75	0.39	1.47
	Zanner 2007	M-AID CPR application on mobile phone versus no support	Proportion: initial check for consciousness	0.96	0.88	1.04
	Zanner 2007	M-AID CPR application on mobile phone versus no support	Proportion: mouth-to-mouth resuscitation	0.71	0.44	1.15
	Zanner 2007	M-AID CPR application on mobile phone versus no support	Proportion: upper airway inspection	1.78	1.09	2.92
	Yang 2008 [161]	Interactive voice and video CPR instructions versus voice only	Proportion of inflation volume >500 ml	1.67	1.03	2.70
	Choa 2008	Animation-assisted CPR instructions on mobile phone versus no assistance	Ventilation: adequate volume	1.00	0.07	13.37
	Choa 2008	Animation-assisted CPR instructions on mobile phone versus no assistance	Compression: correct position	1.25	0.52	3.00
	Choa 2008	Animation-assisted CPR instructions on mobile phone versus no assistance	Ventilation: adequate flow rate	0.50	0.06	4.47
	Bolle 2009	CPR instructions via video call versus audio call	Compression: proportion done without error	0.90	0.14	5.92
	Merchant 2010	Pre-recorded message versus no assistance	Compression: hand placement	1.28	1.12	1.47
	Zanner 2007	M-AID CPR application on mobile phone versus no support	Proportion: proper position	1.59	0.80	3.16
	Yang 2008	Interactive voice and video CPR instructions versus voice only	Open airway	1.59	1.25	2.02
	Yang 2008	Interactive voice and video CPR instructions versus voice only	Open airway: lift chin	1.49	1.19	1.87
	Yang 2008	Interactive voice and video CPR instructions versus voice only	Open airway: tilt forehead	1.12	0.97	1.30
	Yang 2008	Interactive voice and video CPR instructions versus voice only	Proportion of inflation volumes between 500 ml and 600 ml	0.62	0.12	3.21
	Yang 2008	Interactive voice and video CPR instructions versus voice only	Ventilation: open airway	1.46	1.15	1.87
	Yang 2008	Interactive voice and video CPR instructions versus voice only	Ventilation: pinch nose	1.13	1.00	1.26
	Yang 2008	Interactive voice and video CPR instructions versus voice only	Visible chest rise	2.38	1.43	3.94
	Bolle 2009	CPR instructions via video call versus audio call	Compression: proportion done to correct depth	1.12	0.52	2.41
	Yang 2009	Interactive voice and video CPR instructions versus voice only	Compression: proportion done to correct depth	20.86	1.24	351.52
	Merchant 2010	Pre-recorded message versus no assistance	Compression: rate	56.00	55.25	56.75
	Merchant 2010	Pre-recorded message versus no assistance	Compression: depth	10.00	9.48	10.52
	Yang 2008	Interactive voice and video CPR instructions versus voice only	Ventilations: average volume	198.50	2.32	394.68
	Ertl 2007 [162]	Interactive CPR instructions on PDA versus no support	First aid skills score	7.10	5.88	8.32
	Merchant 2010	Pre-recorded message versus no assistance	Compression: time to first	30.00	29.65	30.35
	Choa 2008	Animation-assisted CPR instructions on mobile phone versus no assistance	Compression: time to first	−39.50	−41.36	−37.64
	Merchant 2010	Pre-recorded message versus no assistance	Compression: mean total pauses	−15.00	−16.03	−13.97
	Choa 2008	Animation-assisted CPR instructions on mobile phone versus no assistance	Time to complete first CPR cycle	−44.10	−46.16	−42.04
**Other Secondary Outcomes**	Choi 2009	Discharge instructions by SMS versus usual procedure	Improvement in comprehension of discharge instructions.	0.30	0.07	0.53

CPR, cardiopulmonary resuscitation; LCI, lower confidence interval; UCI,upper confidence interval.

**Table 17:** Secondary outcomes for chronic disease management trials.

Area	Trial	Intervention	Outcome	MD/RR	LCI	UCI
**Adherence**	Lester 2010 [37]	SMS intervention versus no SMS	Self-reported adherence (>95%)	0.81	0.69	0.94
	Cocosila 2009 [163]	SMS reminder versus no SMS	Percent of participants reporting increased adherence	1.38	1.11	1.71
	Ollivier 2009 [164]	SMS reminder versus no SMS	Percent participants discontinuing malaria prophylaxis early	0.85	0.67	1.07
	Yang 2008 [161]	Telephone reminder	Percent participants taking pill/dose every day	1.16	0.93	1.46
	Armstrong 2009 [165]	SMS reminder versus no SMS	Self-rated adherence score (sunscreen application)	0.28	−0.22	0.78
	Armstrong 2009	SMS reminder versus no SMS	Mean percent of days adherent to sunscreen (6 wk)	26.10	24.60	27.60
	Cocosila 2009	SMS reminder versus no SMS	Number of doses missed in last 7 d	−0.80	−1.55	−0.05
**Asthma**	Ostojic 2005 [47]	Asthma monitoring using SMS versus no reminder	Average symptom score - wheezing	−0.09	−0.90	0.72
	Ostojic 2005	Asthma monitoring using SMS versus no reminder	Average symptom score - maximal tolerated activity	−0.30	−0.92	0.32
	Ostojic 2005	Asthma monitoring using SMS versus no reminder	Average symptom score - sleep quality	−0.37	−0.64	−0.10
	Ostojic 2005	Asthma monitoring using SMS versus no reminder	Average symptom score - cough	−0.43	−0.79	−0.07
**Diabetes**	Hanauer 2009 [136]	Diabetes management using mobile phone versus no device	Usage of reminder system - response rate	3.16	0.17	60.28
	Quinn 2008 [140]	Diabetes management using mobile phone versus no device	Medication errors identified	13.00	0.81	209.42
	Quinn 2008	Diabetes management using mobile phone versus no device	New diagnosis of depression	0.50	0.05	4.86
	Quinn 2008	Diabetes management using mobile phone versus no device	Self-reported control issues - confident about diabetes control	1.42	0.98	2.07
	Quinn 2008	Diabetes management using mobile phone versus no device	Self-reported control issues - management improved by receipt of blood sugars	3.00	1.39	6.46
	Quinn 2008	Diabetes management using mobile phone versus no device	Self-reported control issues - improved knowledge of food choices	1.83	0.98	3.45
	Quinn 2008	Diabetes management using mobile phone versus no device	Physician satisfaction - received more patient data during trial period	3.86	1.55	9.61
	Quinn 2008	Diabetes management using mobile phone versus no device	Physician satisfaction - patient self-management skills improved	9.00	1.98	40.97
	Quinn 2008	Diabetes management using mobile phone versus no device	Prescription safety - Medication intensified	3.67	1.32	10.16
	Faridi 2008 [44]	Diabetes support using mobile phone versus no device	Diabetes self care activities - general diet	−0.40	−1.87	1.07
	Faridi 2008	Diabetes support using mobile phone versus no device	Diabetes self care activities - blood glucose testing	0.40	−1.07	1.87
	Faridi 2008	Diabetes support using mobile phone versus no device	Diabetes self care activities - exercise	−0.40	−2.09	1.29
	Faridi 2008	Diabetes support using mobile phone versus no device	Diabetes self care activities - number of cigarettes/day	6.00	1.52	10.48
	Faridi 2008	Diabetes support using mobile phone versus no device	Diabetes self care activities - foot care	−0.60	−1.30	0.10
	Faridi 2008	Diabetes support using mobile phone versus no device	Diabetes self care activities - specific diet	−0.10	−1.16	0.96
	Faridi 2008	Diabetes support using mobile phone versus no device	Diabetes self care activities - medications	−0.10	−0.92	0.72
	Faridi 2008	Diabetes support using mobile phone versus no device	Diabetes self-efficacy scale - exercise	−0.70	−1.50	0.10
	Faridi 2008	Diabetes support using mobile phone versus no device	Diabetes self-efficacy scale - diet	−0.40	−1.33	0.53
	Faridi 2008	Diabetes support using mobile phone versus no device	Diabetes self-efficacy scale - self-treat	−0.60	−1.49	0.29
	Faridi 2008	Diabetes support using mobile phone versus no device	Diabetes self-efficacy scale - routines	−0.20	−0.99	0.59
	Faridi 2008	Diabetes support using mobile phone versus no device	Diabetes self-efficacy scale - total	−0.50	−1.09	0.09
	Faridi 2008	Diabetes support using mobile phone versus no device	Diabetes self-efficacy scale - certainty	−0.90	−1.99	0.19
	Franklin 2006	SMS support system versus no SMS	Diabetes self-efficacy scale -Score	6.10	0.45	11.75
	Faridi 2008	Diabetes support using mobile phone versus no device	Physical activity – walking	−1955.00	−4685.55	775.55
	Franklin 2006	SMS support system versus no SMS	diabetes knowledge scales	−0.50	−1.60	0.60
	Faridi 2008	Diabetes support using mobile phone versus no device	Yale physical activity scale - standing	0.30	−0.92	1.52
	Faridi 2008	Diabetes support using mobile phone versus no device	Yale physical activity scale - sitting	0.30	−0.88	1.48
	Faridi 2008	Diabetes support using mobile phone versus no device	Yale physical activity scale - leisurely walk	0.30	−11.55	12.15
	Faridi 2008	Diabetes support using mobile phone versus no device	Yale physical activity scale -vigorous activity	−1.40	−14.64	11.84
	Faridi 2008	Diabetes support using mobile phone versus no device	Yale physical activity scale -moving around	−2.70	−5.26	−0.14
	Franklin 2006	SMS support system versus no SMS	diabetes social support – insulin	2.30	0.38	4.22
	Franklin 2006	SMS support system versus no SMS	diabetes social support - diet	6.20	4.22	8.18
	Franklin 2006	SMS support system versus no SMS	diabetes social support - exercise	4.40	2.69	6.11
	Franklin 2006	SMS support system versus no SMS	diabetes social support - blood glucose testing	4.70	2.70	6.70
	Benhamou 2007 [43]	Diabetes management using PDA	Quality of life (general) - Score	0.50	−4.23	5.23
	Benhamou 2007	Diabetes management using PDA	Satisfaction with life – Score	0.40	−6.64	7.44
	Franklin 2006	SMS support system versus no SMS	visual analogue adherence score	6.80	−2.58	16.18
**Hypertension**	Marquez Contreras 2004	Advisory SMS messages versus no SMS	proportion of pills taken to no. of pills should have taken	8.90	0.18	17.62
	Carrasco 2008	Hypertension management on mobile phone versus no device	Quality of life (mental) - SF36 score	−0.10	−2.35	2.15
	Carrasco 2008	Hypertension management on mobile phone versus no device	Quality of life (physical) - SF36 score	3.00	1.04	4.96
	Carrasco 2008	Hypertension management on mobile phone versus no device	State trait anxiety inventory - state anxiety	−3.60	−6.01	−1.19
	Carrasco 2008	Hypertension management on mobile phone versus no device	State trait anxiety inventory - trait anxiety	−1.70	−3.88	0.48
	Carrasco 2008	Hypertension management on mobile phone versus no device	Self-monitoring - Systolic blood pressure	−0.80	−4.09	2.49
	Carrasco 2008	Hypertension management on mobile phone versus no device	Self-monitoring - heart rate	0.40	−2.61	3.41
	Carrasco 2008	Hypertension management on mobile phone versus no device	Self-monitoring - Diastolic blood pressure	0.70	−1.27	2.67
**Chemotherapy monitoring**	Kearney 2009 [155]	Questionnaire by SMS versus Paper	Chemotherapy symptom - sore mouth/throat	1.26	0.84	1.89
	Kearney 2009	Questionnaire by SMS versus Paper	Chemotherapy symptom – diarrhoea	1.13	0.64	1.97
	Kearney 2009	Questionnaire by SMS versus Paper	Chemotherapy symptom - fatigue	0.82	0.65	1.03
	Kearney 2009	Questionnaire by SMS versus Paper	Chemotherapy symptom - vomiting	0.92	0.44	1.90
	Kearney 2009	Questionnaire by SMS versus Paper	Chemotherapy symptom - nausea	0.88	0.64	1.22
	Kearney 2009	Questionnaire by SMS versus Paper	Chemotherapy symptom - hand foot syndrome	2.17	0.89	5.30
	Walker 2004 [157]	Data recording on handheld computer versus Paper	Number of patients with an error in the number of vials not accounted for	1.06	0.67	1.67
	Kearney 2009	Questionnaire by SMS versus Paper	Chemotherapy distress - sore mouth/throat	0.19	−0.03	0.41
	Kearney 2009	Questionnaire by SMS versus Paper	Chemotherapy distress - diarrhoea	−0.28	−0.61	0.05
	Kearney 2009	Questionnaire by SMS versus Paper	Chemotherapy distress - fatigue	−0.13	−0.46	0.20
	Kearney 2009	Questionnaire by SMS versus Paper	Chemotherapy distress - vomiting	0.03	−0.19	0.25
	Kearney 2009	Questionnaire by SMS versus Paper	Chemotherapy distress - nausea	0.14	−0.01	0.29
	Kearney 2009	Questionnaire by SMS versus Paper	Chemotherapy distress -hand foot syndrome	0.00	−0.18	0.18
	Kearney 2009	Questionnaire by SMS versus Paper	Chemotherapy severity - sore mouth/throat	−0.20	−0.62	0.22
	Kearney 2009	Questionnaire by SMS versus Paper	Chemotherapy severity - diarrhoea	0.01	−0.31	0.33
	Kearney 2009	Questionnaire by SMS versus Paper	Chemotherapy severity - fatigue	−0.28	−0.69	0.13
	Kearney 2009	Questionnaire by SMS versus Paper	Chemotherapy severity- vomiting	0.27	−0.04	0.58
	Kearney 2009	Questionnaire by SMS versus Paper	Chemotherapy severity- nausea	0.04	−0.23	0.31
	Kearney 2009	Questionnaire by SMS versus Paper	Chemotherapy severity -hand foot syndrome	0.24	0.03	0.45
**Lung transplant monitoring**	Devito Dabbs 2009 [38]	Personal assistant for tracking health on PDA for patients post-lung transplant	Perceived self-care agency	21.34	18.19	24.49
**Antenatal support**	Jareethum 2008 [49]	Pre-natal support via SMS versus no SMS	Anxiety	−2.15	−3.42	−0.88
	Jareethum 2008	Pre-natal support via SMS versus no SMS	Confidence level	1.12	0.51	1.73
	Jareethum 2008	Pre-natal support via SMS versus no SMS	Satisfaction level	1.25	0.78	1.72
	Jareethum 2008	Pre-natal support via SMS versus no SMS	Anxiety	−1.01	−2.28	0.26
	Jareethum 2008	Pre-natal support via SMS versus no SMS	Confidence level	0.56	−0.06	1.18
	Jareethum 2008	Pre-natal support via SMS versus no SMS	Satisfaction level	1.19	0.37	2.01
**Psychological support**	Pijnenborg 2010 [152]	SMS reminder to improve goal achievement versus no treatment	Completion of activities	51.00	37.49	64.51
	Pijnenborg 2010	SMS reminder to improve goal achievement versus no treatment	Appointment attendance	9.00	−4.37	22.37
	Pijnenborg 2010	SMS reminder to improve goal achievement versus no treatment	Reduction in undesired behaviour	23.00	14.53	31.47
	Pijnenborg 2010	SMS reminder to improve goal achievement versus no treatment	Medication adherence	17.00	4.76	29.24
	Pijnenborg 2010	SMS reminder to improve goal achievement versus no treatment	Training programmes	14.00	−1.21	29.21
	Grassi 2009 [153]	Audio relaxation sessions versus none	State trait anxiety inventory	−7.07	−12.07	−2.07
	Grassi 2009	Video relaxation sessions versus none	State trait anxiety inventory	0.17	−5.54	5.88
	Grassi 2009	Audio+Video relaxation sessions versus none	State trait anxiety inventory	0.23	−5.17	5.63
	Grassi 2009	Audio+Video relaxation sessions versus none	Visual analog scale - relax	0.23	−0.36	0.82
	Grassi 2009	Audio relaxation sessions versus none	Visual analog scale - relax	1.70	1.05	2.35
	Grassi 2009	Video relaxation sessions versus none	Visual analog scale - relax	0.13	−0.57	0.83
	Grassi 2009	Audio+Video relaxation sessions versus none	Visual analog scale - anxiety	−0.50	−1.10	0.10
	Grassi 2009	Audio relaxation sessions versus none	Visual analog scale - anxiety	−1.03	−1.54	−0.52
	Grassi 2009	Video relaxation sessions versus none	Visual analog scale - anxiety	−0.10	−0.72	0.52
	Patel 2006 [150]	Handheld video game to reduce anxiety pre-surgery versus no device	Modified Yale preoperative anxiety scale	−9.80	−11.65	−7.95
	Patel 2006	Handheld video game to reduce anxiety pre-surgery versus no device	Post-hospital behaviour questionnaire: assesses post-operative anxiety-related behaviour.	0.40	0.05	0.75

LCI, lower confidence interval; UCI, upper confidence interval.

### Health Behaviour Change

**Figure 4:**
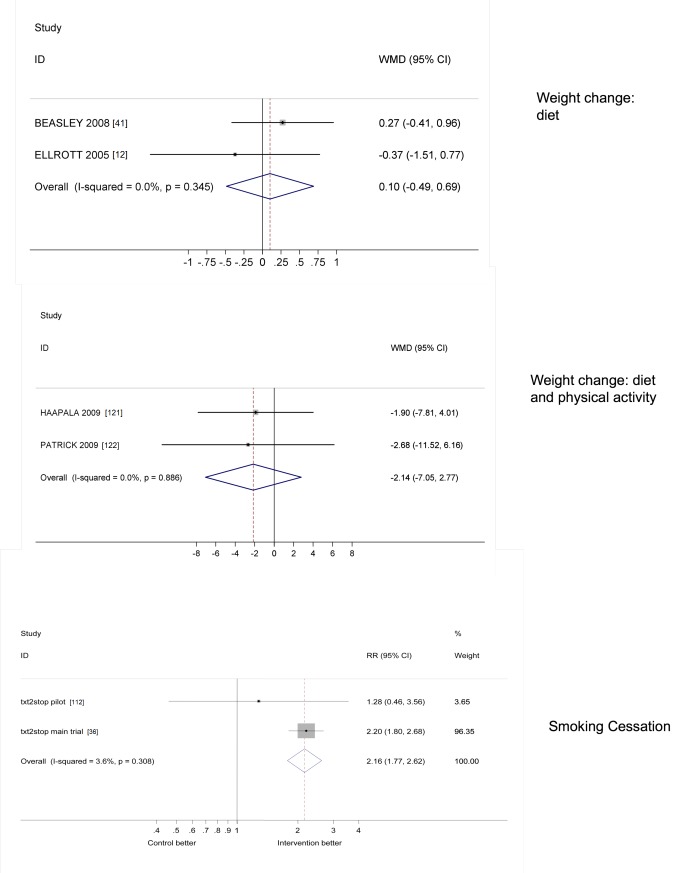
Health behavior change interventions.

Pooled effects for health behaviour change are reported in Figure 4 and outcomes are reported in Table 18.

**Table 18:** Primary outcomes for health behaviour change interventions.

Clinical Area	Trial	Intervention	Outcome	RR/SMD	LCI	UCI
**Smoking**	Rodgers 2005 [39]	5 SMS/day versus no SMS	Smoking cessation - 6 wk	2.84	1.12	7.16
	Vidrine 2006 [114]	Counselling sessions on mobile phone versus usual care	Smoking cessation - 3 mo	2.74	0.78	9.55
	Vidrine 2006	Counselling sessions on mobile phone versus usual care	24-h point prevalence of smoking cessation (at 3-mo follow-up - biochemical)	3.59	1.30	9.94
**Physical activity**	Newton 2009 [42]	Motivational SMS versus standard care	Body mass index (kg/m^2^)	−0.01	−0.03	0.01
	Prestwich 2010 [96]	SMS goal reminder versus no SMS	Waist-to-hip ratio	0.00	−0.03	0.03
	Prestwich 2009 [118]	SMS plan reminder versus no SMS	Waist-to-hip ratio	−0.01	−0.03	0.02
	Prestwich 2010	SMS goal reminder versus no SMS	Weight change	1.19	−4.47	6.85
	Prestwich 2009	SMS plan reminder versus no SMS	Weight change	0.29	−5.39	5.98
	Newton 2009	Motivational SMS versus standard care	Systolic blood pressure - change (mmHg)	2.10	0.30	3.90
	Newton 2009	Motivational SMS versus standard care	Diastolic blood pressure - change (mmHg)	1.30	0.12	2.48
	Newton 2009	Motivational SMS versus standard care	Blood sugar control - change (% HbA1C)	0.37	0.28	0.46
	Newton 2009	Motivational SMS versus standard care	Insulin intake - change (units per kg)	0.00	−0.02	0.03
	Liu 2008 [116]	Endurance exercises accompanied by music on mobile phone versus booklet and DVD	Post-exercise breathlessness - Borg scale score	−0.70	−0.81	−0.59
	Newton 2009	Motivational SMS versus standard care	Physical activity - walking (steps per day) - change	818.00	533.17	1102.83
**Diet and physical activity**	Haapala 2009 [121]	SMS versus no SMS	Body weight - change (percent)	0.00	−2.81	2.81
	Haapala 2009	SMS versus no SMS	Waist circumference – change (cm)	−2.00	−6.93	2.93
	Shay 2009 [124]	Diary on PDA	Body fat - change (percent)	0.40	−1.55	2.35
	Shay 2009	Diary on PDA	Body weight - change (kg)	0.10	−2.88	3.08
	Shay 2009	Diary on PDA	Waist circumference - change (inches)	1.10	−0.58	2.78
	Turner-Mcgrievy 2009 [126]	Weight-loss podcast	Body mass index (kg/m^2^)	−0.50	−2.25	1.25
	Turner-Mcgrievy 2009	Weight-loss podcast	Weight (kg)	0.30	−5.86	6.46
	Burke 2010 [40]	PDA plus feedback	Body weight - change (>5% weight loss)	−2.00	−4.06	0.06
	Burke 2010	PDA only	Body weight - change (>5% weight loss)	−0.20	−2.35	1.95
	Burke 2010	PDA plus feedback	Waist circumference - change (percent)	−2.30	−4.24	−0.36
	Burke 2010	PDA only	Waist circumference - change (percent)	−0.90	−2.79	0.99
**Diet**	Beasley 2008 [41]	DietMatePro software on PDA	Waist circumference - change (inches)	0.50	0.08	0.92
	Beasley 2008	DietMatePro software on PDA	Body weight - change (pounds)	−0.60	0.91	2.11

LCI, lower confidence interval; UCI, upper confidence interval, SMD, standardised mean difference.

#### Primary objective outcomes

Two health behaviour change trial had low risk of bias. SMS-based smoking cessation interventions more than doubled biochemically-verified smoking cessation at 6 months [21],[36] (pooled effect estimate relative risk [RR] 2.16 [95% CI 1.77–2.62, *p*<0.0001]).

For other trials, of three smoking cessation trials reporting biochemicallyverified smoking cessation outcomes, two showed statistically significant improvement [36],[39]. There were no statistically or clinically significant changes in weight for trials using SMS messages to reduce calorie intake and increase physical activity (standard mean difference [SMD] −2.14 [95% CI −7.05 to 2.77] kg) or for trials using application software to reduce calorie intake (SMD −0.10 [95% CI −0.49 to 0.69] kg). One trial of a diet and physical activity intervention and one other trial of an intervention targeting diet only showed statistically significant reductions in waist circumference [40],[41]. The other 13 anthropometric outcomes for trials targeting physical activity only, diet only, or diet and physical activity showed no statistically significant effects (Table 18). One trial [42] targeting physical activity reported four disease control outcomes: one showed a statistically significant benefit in diabetes control (HBA1C) [42], one showed no effect on insulin requirements [42], and two showed no change in systolic and diastolic blood pressure. One objective measure of physical activity showed a statistically significant increase in the number of steps taken per day as assessed by a pedometer. There were no primary outcomes reported for sexual health or alcohol consumption [42].

#### Secondary outcomes

Table 15 shows the effect estimates for secondary outcomes for health behaviour change interventions. For smoking cessation support, eight of the 14 self-reported smoking outcomes showed statistically significant benefits and none showed statistically significant harms. For physical activity, diet, and diet and physical activity trials, seven of the 14 self-reported physical activity outcomes reported showed statistically significant benefits and none showed statistically significant harms. None of the 17 dietary intake outcomes reported showed statistically significant effects. There were six reported outcomes regarding cognitive mediators of behaviour change. Of these, three showed statistically significant benefits and none showed statistically significant harms. For the trial targeting safer sexual behaviour, the cognitive mediator of behaviour change (self-efficacy regarding condom use) reported showed a statistically significant benefit.

### Disease Management

#### Primary outcomes

**Figure 5:**
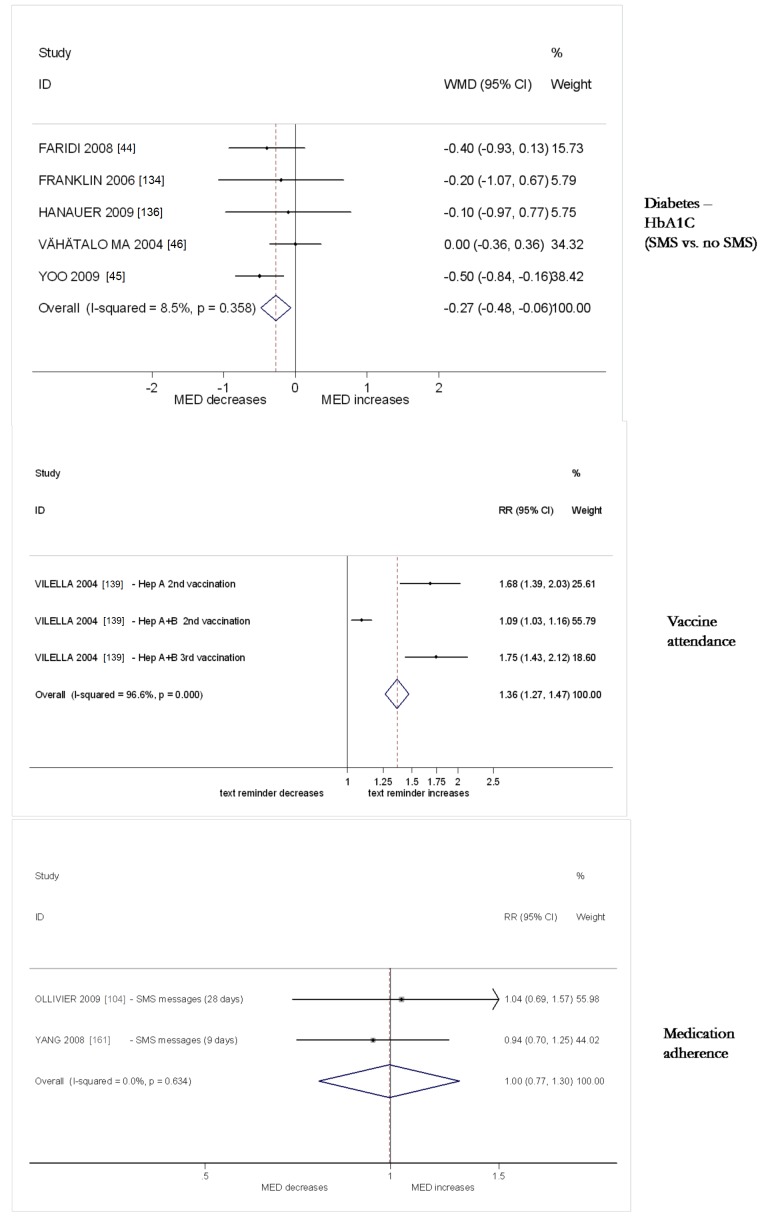
Self-management of disease.

Pooled effects are reported in Figure 5 and outcomes are reported in Table 19.

**Table 19:** Primary outcomes for disease management.

Clinical Area	Trial	Intervention	Outcome	MD/RR	LCI	UCI
Adherence	Lester 2010 [37]	SMS reminder versus no SMS	Viral load of <400 copies/ml	0.85	0.72	0.99
	Lester 2010	SMS reminder versus no SMS	Mortality	0.79	0.47	1.32
Asthma	Ostojic 2005 [47]	Asthma monitoring using SMS versus no reminder	Lung function - PEF variability	−11.12	−19.56	−2.68
	Ostojic 2005	Asthma monitoring using SMS versus no reminder	Lung function - FEV1	3.00	−15.91	21.91
	Ostojic 2005	Asthma monitoring using SMS versus no reminder	Lung function - FVC	−1.37	−16.33	13.59
Diabetes	Vahatalo Ma 2004 [46]	Diabetes management using mobile phone versus no device	Percentage weight loss (1 y)	−0.60	−5.07	3.87
	Benhamou 2007 [43]	Diabetes management using PDA versus no device	HbA1C blood concentration	−0.16	−0.48	0.16
	Holman 1996 [137]	Diabetes management using pocket computer	HbA1C blood concentration	0.00	−0.62	0.62
	Schrezenmeir 2006 [142]	Diabetes management using pocket computer	Hb1AC blood concentration	−11.90	−13.59	−10.21
	Benhamou 2007	Diabetes management using PDA versus no device	Blood sugar control – glycaemia	−7.00	−17.38	3.38
	Holman 1996	Diabetes management using pocket computer versus no device	Blood sugar control - pre-prandial glucose	−1.40	−1.90	−0.90
	Holman 1996	Diabetes management using pocket computer versus no device	Blood sugar control - fructosamine	−11.00	−53.15	31.15
	Schrezenmeir 2006	Diabetes management using pocket computer versus no device	Blood glucose (average)	−1.00	−1.12	−0.88
	Schrezenmeir 2006	Diabetes management using pocket computer versus no device	Blood glucose in the morning	−1.30	−1.50	−1.10
	Schrezenmeir 2006	Diabetes management using pocket computer versus no device	Blood glucose in the evening	−1.10	−1.24	−0.96
	Schrezenmeir 2006	Diabetes management using pocket computer versus no device	Blood glucose amplitudes breakfast - lunch	−0.30	−0.47	−0.13
	Vahatalo Ma 2004	Diabetes management using mobile phone versus no device	Daily insulin requirements	6.40	1.09	11.71
	Yoo 2009 [45]	Diabetes management using mobile phone versus no device	Fasting plasma glucose levels	0.00	−0.66	0.66
	Holman 1996	Diabetes management using pocket computer versus no device	Ultralente insulin dose (per day)	2.50	−5.31	10.31
	Holman 1996	Diabetes management using pocket computer versus no device	Soluble insulin dose (per day)	0.00	−4.04	4.04
	Yoo 2009	Diabetes management using mobile phone versus no device	Homeostasis model assessment insulin resistance	0.20	−0.30	0.70
	Faridi 2008 [44]	Diabetes support using mobile phone versus no device	Systolic blood pressure	−7.10	−21.94	7.74
	Faridi 2008	Diabetes support using mobile phone versus no device	Diastolic blood pressure	−6.60	−14.40	1.20
	Yoo 2009	Diabetes management using mobile phone versus no device	Lipid profile - total cholesterol	−0.40	−0.68	−0.12
	Yoo 2009	Diabetes management using mobile phone versus no device	Lipid profile - LDL cholesterol	−0.10	−0.34	0.14
	Yoo 2009	Diabetes management using mobile phone versus no device	Lipid profile - HDL cholesterol	0.00	−0.11	0.11
	Yoo 2009	Diabetes management using mobile phone versus no device	Left brachial-ankle pulse wave velocity	−39.00	−150.55	72.55
	Yoo 2009	Diabetes management using mobile phone versus no device	Right brachial-ankle pulse wave velocity	−32.00	−142.20	78.20
	Franklin 2006 [135]	SMS support system versus no SMS	Health service utilisation - number of clinic visits	0.30	−0.22	0.82
Hypertension	Carrasco 2008 [48]	Hypertension management on mobile phone versus no device	Diastolic blood pressures	0.20	−2.24	2.64
	Marquez Contreras 2004 [147]	Advisory SMS messages versus no SMS	Diastolic blood pressure	1.84	−2.14	5.82
	Carrasco 2008	Hypertension management on mobile phone versus no device	Systolic blood pressure	−4.30	−8.35	−0.25
	Marquez Contreras 2004	Advisory SMS messages versus no SMS	Systolic blood pressure	1.10	−4.37	6.57
	Marquez Contreras 2004	Advisory SMS messages versus no SMS	Weight in kg	−2.76	−8.17	2.65
Heart failure	Scherr 2009 [156]	Telemonitoring using mobile phone versus usual care	hospitalization for worsening CHF or death from cardiovascular cause	0.53	0.26	1.08
	Scherr 2009	Telemonitoring using mobile phone versus usual care	Cardiovascular health - ejection fraction	1.00	0.59	1.70
Antenatal support	Jareethum 2008 [49]	Pre-natal support via SMS versus no SMS	Caesarean section	1.13	0.34	3.82
	Jareethum 2008	Pre-natal support via SMS versus no SMS	Normal vaginal delivery	0.98	0.78	1.24
	Jareethum 2008	Pre-natal support via SMS versus no SMS	Fetal birth-weight	−137.00	−412.87	138.87
	Jareethum 2008	Pre-natal support via SMS versus no SMS	Gestational age at delivery	0.10	−0.45	0.65
	Jareethum 2008	Pre-natal support via SMS versus no SMS	Preterm delivery	0.18	0.01	3.64

CHF, chronic heart failure; HDL, high density lipoprotein; LCI, lower confidence interval; LDL, low density lipoprotein; PEF, peak expiratory flow; FEV1, forced expiratory volume in one second; FVC; forced vital capacity; UCI, upper confidence interval.

A trial with low risk of bias dealt with adherence to prescribed medication, in which an intervention that used text messages to maintain contact, monitor, and respond to medication issues in patients on antiretrovirals statistically significantly reduced HIV viral load <400 copies (RR 0.85 [95% CI 0.72–0.99]) [37] but did not reduce mortality (RR 0.79 [95% CI 0.47 to 1.32]). The other trial with low risk of bias did not report any primary outcomes [38].

Diabetes self-management intervention reduced HBA1C (pooled mean difference [MD] −0.27% [95% CI −0.48 to −0.06]) [43]. For 12 other diabetes control outcomes, six showed statistically significant benefits. Two trials [44] reported two blood pressure outcomes; neither showed reductions that were statistically significant. One trial reported three lipid profile outcomes, of which one showed a statistically significant reduction [45]. One trial reported no effect on weight loss [46]. No trials reported outcomes of hypoglycaemia.

For patients with asthma, one trial [47]reported three lung function outcomes, of which one showed a statistically significant improvement and none showed statistically significant negative effects. For patients with hypertension, of four blood pressure outcomes reported in two trials, one showed a statistically significant reduction [48] and none showed statistically significant negative effects. There were no statistically significant effects reported for a trial for monitoring of heart failure. For antenatal support there were no statistically significant effects reported for five outcomes reported in one trial [49].

#### Secondary outcomes

Secondary outcomes are reported in Tables 16 and 17.

One trial at low risk of bias was an adherence intervention that reduced self-reported non adherence to antiretroviral medication (RR 0.80 [95% CI 0.69–0.94]).The other trial with low risk of bias reported an increase in self care agency in patients with lung transplants [38].

Other trials included SMS reminders that had no effect on the percentage of pills taken daily in two trials (RR 1.00 [95% CI 0.77–1.30]) with *I* ^2^ = 0%. Of six other adherence outcomes reported by four trials, three showed statistically significant benefits and none showed statistically significant harms. In one trial, the pooled effect of SMS reminders on attendance for vaccination at different time points was RR 1.19 (95% CI 1.15–1.23) with *I* ^2^ = 99.7% (*p*>0.001). For cardiopulmonary resuscitation (CPR), of 37 outcomes reported in nine trials, 18 showed statistically significant benefits and none showed statistically significant reductions in the quality of CPR. For diabetes self-management interventions, of 26 medical process outcomes reported, five showed statistically significant benefits and two showed statistically significant reductions in beneficial medical processes. Of 11 cognitive or mediator outcomes, five showed statistically significant benefits and none showed statistically significant harms. For hypertension, of three self-monitoring outcomes, none had statistically significant effects and one of two quality of life outcomes showed a statistically significant benefit. One of two anxiety outcomes showed a statistically significant benefit and there was no effect on self-reported adherence to hypertension medication. There were no statistically significant effects on 19 outcomes relating to chemotherapy symptom monitoring. One lung transplant monitoring intervention reported a statistically significant increase in perceived self-care agency. For one trial of ante-natal support, four of the six outcomes regarding anxiety, confidence, and satisfaction showed statistically significant benefits and none showed statistically significant harms. For psychological support interventions, three of the seven anxiety outcomes showed statistically significant reductions in anxiety and five of the nine other outcomes showed statistically significant benefits. There were no statistically significant reductions reported in psychological health outcomes.

## Discussion

### Key Findings

This review of randomised controlled trials for mobile technology interventions delivered to health care consumers found 59 trials that investigated the use of mobile technologies to improve disease management and 26 trials that investigated their use to change health behaviours. The trial demonstrated mixed evidence regarding the benefits of interventions, and nearly all trials were conducted in high-income countries. Four trials had a low risk of bias. One trial with low risk of bias reported clinically important reductions in viral load from an intervention that used text messages to maintain contact, monitor, and respond to medication issues with patients prescribed antiretrovirals in Kenya [37]. Trials demonstrate that automated multifaceted smoking cessation support delivered by text message more than doubles biochemically verified smoking cessation [21],[36]. However, our meta-analyses show that, to date, mobile technology-based interventions for diabetes control that have statistically significant effects are small and of borderline clinical importance. Simple medication reminders delivered by SMS message show no benefits. The effect estimates for diet and diet with physical activity interventions on weight were consistent with no or small benefits. There is suggestive evidence of benefit for reminders for vaccine appointment attendance and cardiopulmonary resuscitation training. There is suggestive evidence of short-term benefits for interventions for asthma control, physical activity, and psychological support interventions, which could be clinically important if these effects were demonstrated in the long term. Our review found that few trials were at low risk of bias in all areas assessed. Few trials were conducted in low- or middle-income countries.

#### Strengths and weaknesses of the systematic review in relation to other systematic reviews

To our knowledge, this is the first systematic review of all mobile technology interventions delivered to health care consumers. Our review updates earlier reviews and expands them either by using a more exhaustive search strategy, by presenting effect estimates and 95% confidence intervals for all reported outcomes [28],[29],[31], or by conducting meta-analysis, where sufficient trials report similar outcomes [28],[29],[31]. Nineteen trials (25%) did not provide sufficient data to calculate effect estimates and authors did not respond to requests for data; this could have resulted in bias in the systematic review findings. It was beyond the scope of our review to review all internet or video-based interventions, which in principal can be viewed on many modern mobile phones. Our review aimed to examine the effects of interventions delivered by mobile technologies alone, so we excluded interventions combining mobile technologies with other interventions such as face-to-face counselling. These interventions could be subject to a separate systematic review. A wide range of factors could influence the effectiveness of mobile technology interventions including trial quality [50]; participant factors such as demographics (gender/age) or disease status (disease management/disease prevention interventions); the setting (low-/middle- or high-income country), intervention factors such as components, intensity, timing, type of mobile device (e.g., PDA or mobile phone); or the mobile technology function used (e.g., SMS, video, application software). It was not possible to explore the factors influencing heterogeneity statistically, as there were few trials of similar interventions reporting the same outcomes, resulting in limited power for such analyses. The examination of funnel plots in exploring publication bias was limited. as few trials contributed to some pooled analyses.

We used Abraham and Michie's typology [34] of behaviour change tools to code the intervention content according to author descriptions in the papers or protocols. The typology could be improved by modifying it for mobile technology-based interventions and specific behavioural domains, as has been done for smoking cessation [51]. We coded the authors' descriptions of the interventions as reported in the papers, which varied in the level of detail provided. The coding of intervention content would have been more complete and accurate if the text message and other intervention content was obtained and directly coded, as has been done for the txt2stop intervention [51]. There was inadequate power to explore the impact of different behaviour change tools in specific behavioural domains on effect estimates.

#### Systematic review findings in relation to findings from trials published since our search was completed

On 1 November 2012, we re-ran our search to identify new trials published since the search conducted for the systematic review was completed.

### Health Behaviour Change

We identified 14 new trials of health behaviour change interventions. Primary outcomes were as follows: One trial reported increases in smoking cessation at 1 wk for mobile phone-based telephone counselling intervention [52]. A trial involving video-based smoking cessation support showed no benefits [53]. Three trials reported no statistically significant effects on weight of a diary-plus-mobile-phone daily feedback messages compared to a diary [54], a text message based intervention [55], and a twitter, podcast, and mobile phone application based intervention [56]. A mobile phone-based counselling intervention had no effect on postponing subsequent pregnancies on teen mothers [57]. There was no effect on plaque score of text message based oral health education [58].

Secondary outcomes were as follows: One trial reported pregnant smokers receiving text messages were more likely to set a quit date [59]. One trial reported a reduction in portion sizes for a mobile phone and web based planning intervention designed to reduce weight [60]. One trial reported an increase in testing for HIV in those receiving a text message intervention involving ten or more messages [61]. One trial reported increases in sexually transmitted infection testing amongst those receiving text message based sexual health interventions (odds ratio [OR] 2.51 [95% CI 1.11–5.69]) [62]. One factorial trial reported a reduction in sexual partners in participants receiving safer sex text messages, but no effects of sun protection messages on hat wearing [63]. One trial reported an increase in condom use with “risky partners such as sex workers or with other men” [64]. One trial reported a reduction in self-reported heavy drinking days in young people allocated to a text messages-based intervention [65].

### Disease Management

We identified 37 new trials of disease management interventions. Primary outcomes were as follows: One trial with unclear risk of bias reported improved cure rates for tuberculosis in those receiving medication reminders [66]. One trial employing text messages increased the proportion of urban women delivering with a skilled attendant (OR 5.73 [95% CI 1.51–21.81]); the risk of bias in this trial was unclear [67]. One small trial reported a statistically and clinically important >1% absolute reduction in HBA1C in the intervention group compared to control group [68].Four trials of a diabetes care intervention, three involving text messages and the other involving video messages, reported statistically significant decreases in HBA1C, which were of borderline clinical significance [69]–[73]. One trial with unclear risk of bias reported increases in receipt of vaccinations in those allocated to receive text messages reminders [74]. One trial found no effect of a detailed text message compared to a simple text message inviting women for mammography screening [75]. One trial with unclear/high risk of bias reported an improvement in cardiovascular risk profiles for patients receiving telemonitoring via mobile phone with text message-based advice (RR = 1.4; 95% CI 1.1–1.7). The intervention increased the proportion of patients achieving blood pressure <140/90 (RR 1.4 [1.1–1.9]) and increased the proportion of patients achieving HBA1C of <7% (RR 1.16 [1.6–2.4]), but there were no statistically significant differences in LDL cholesterol or smoking [76]. One trial with unclear risk of bias reported a statistically significant reduction in systolic and diastolic blood pressure compared to baseline in an intervention group receiving monitoring of salt excretion but no statistically significant change in the control group [77]. Two trials reported no statistically significant beneficial effects of either a text message-based intervention or a mobile phone-based monitoring and feedback intervention on asthma control [78],[79]. A trial providing asthma patients with alerts regarding health risk weather forecast had no statistically significant benefits on reducing exacerbations of asthma [80].

Secondary outcomes were as follows: Three trials reported statistically significant increases in adherence to antiretroviral medication with text message reminders [81]–[83], and one trial reported increases in adherence that were not statistically significant [84]. One trial has demonstrated improved quality of life for patients with heart failure receiving a mobile phone based telemonitoring intervention [85]. Two trials reported increases in emotional self-awareness in young people receiving risk assessment and management of youth mental health problems: one reported benefits in mild depressive symptoms [86],[87], and another reported that delivering cognitive behavioural therapy messages by mobile phone was feasible and acceptable [88]. One trial reported improved recall of goals in a trials delivering text messages to patients undergoing brain rehabilitation [89]. One trial reported that an educational package of text messages increased continuation of the combined oral contraceptive pill [90], but simple daily reminders regarding taking the combined oral contraceptive has no effect in another trials [91]. One trial reported an increase in self-efficacy for self-managing cystic fibrosis [92]. A mobile phone-based map did not shorten the time taken to retrieve an automated external defibrillator in out-of-hospital cardiac arrest victims [93]. Three trials reported short term increases in physical activity in those receiving a text message-based intervention designed to increase activity [94]–[96]. Four trials reported benefits in cardiopulmonary resuscitation processes with mobile phone-based audio instructions [97], video instruction [98], and a feedback application [99]. One trial reported improved quality of life with a mobile phone-based asthma self-care system [100], and another reported increases in self-reported adherence to asthma medication [101].

In summary, while interventions using mobile technologies for patients are a fast-moving field, the findings of many trials published since the search for the systematic review was completed are generally consistent with the reported results of trials in the systematic review, particularly for primary outcomes. Trials report benefits in adherence to antiretroviral medication and smoking cessation; one small trial reported a significant reduction in HBA1c for a diabetes control intervention, and there were no or small effects of interventions on weight and no reported benefits of daily medication reminders. There is suggestive evidence of benefit for attendance for vaccination and cardiopulmonary resuscitation interventions. There are short term benefits for physical activity interventions and psychological support interventions that could be clinically significant if sustained.

Novel trial findings published since our search for the systematic review was completed suggest that text messages can increase the proportion of women receiving skilled delivery in urban areas in Kenya. There are now mixed results for asthma control interventions, with some trials reporting benefits. There is suggestive evidence that multifaceted adherence interventions can increase adherence to the combined oral contraceptive pill and TB medication resulting in increased TB cure rates. There is suggestive evidence that a heart failure intervention can improve quality of life and that tele-monitoring data sent by mobile combined with text message advice can reduce blood pressure and HBA1C. There is suggestive evidence that sexual health interventions can increase testing for HIV and sexually transmitted infections.

### Implications of the Study for Health Care Delivery

Multifaceted adherence interventions demonstrate an increase in adherence to anti-retrovirals and a decrease in viral load when evaluated in Kenya [37]. An automated texting intervention for smoking cessation support was effective when evaluated in the UK. Both interventions may require some adaptation as well as translation for use in other countries. Health services should consider implementing similar interventions in similar settings.

The finding that simple SMS reminders for adherence to medication have at best small effects is consistent with the findings from existing adherence research, which has previously demonstrated that uni-faceted interventions have very modest benefits [102]–[104]. Likewise, it is unsurprising that interventions delivered by mobile technologies appear to have small effects on weight, given the paucity of effective behavioural change interventions for obesity [105],[106].

There is currently insufficient high quality evidence of beneficial effects on clinical outcomes to warrant implementation of interventions for other areas of health behaviour change or self-management of diseases.

### Unanswered Questions and Future Research

The benefits of text message support on adherence to antiiretrovirals and smoking cessation should be established in high-, middle-, and low-income countries. The cost-effectiveness of antiretroviral adherence interventions should be established. A cost-effectiveness analysis of mobile phone text messaging interventions for smoking cessation showed that the intervention is cost-effective [107]. Further high quality trials should robustly establish the effects of multifaceted text messaging interventions on medication adherence in other areas, TB cure rates, and on the proportion of births with a skilled attendant at delivery in urban areas in low- and middle-income countries. Adequately powered high quality trials of optimised interventions are required in areas of health behaviour change and self-management of diseases where there is currently suggestive evidence of benefit.

In areas where, to date, interventions have shown small benefits of borderline or no clinical importance future research should focus on improving interventions, drawing on existing guidance for the development of complex interventions, prior to considering further evaluation by randomised controlled trial [108]. There is a considerable body of existing research regarding effective face-to-face interventions for self-management of diseases, and much of this literature suggests multifaceted interventions are required [104],[109]–[111]. Mobile technology-based interventions for health care consume rs developed to date may not be optimal; some are uni-faceted and may need to incorporate additional components found in other effective interventions for self-management of diseases, but modified for delivery by mobile technologies. For health behaviour change interventions, further research is required to elucidate the effects of different behaviour change tools on behaviours and to explore if interventions, which include a wider range of behaviour change tools, have greater effects.

A range of questions regarding the effects of mobile technologies remain open to question including which functions are most effective (SMS, video, oral instruction, application software), which behaviour change techniques are effective, and whether the effectiveness of interventions is influenced by setting or participant demographics.

The majority of the research to date has been conducted in high-income countries, so trials of interventions in low- and middle-income countries are required, particularly in view of the high coverage of mobile technologies in these settings.

### Conclusion

Multifaceted mobile technology text messaging interventions have been shown to increase adherence to antiretroviral medication in a low-income setting and increase smoking cessation in a high-income setting; these interventions should be considered for inclusion in services in similar settings. Their effects in other settings should be established. For other mobile technology health interventions delivered to health care consumers. the effects of optimised interventions on long term, clinically important outcomes must be robustly established in randomised controlled trials.

## Supporting Information

Table S1 **Excluded studies.**
(DOCX)

Text S1 **PRISMA 2009 checklist.**
(DOC)

Text S2 **The effectiveness of M-health technologies for improving health and health services: a systematic review protocol.**
(PDF)

Text S3 **MEDLINE (Ovid) search strategy.**
(DOC)

## Abbreviations

ART: antiretroviral
PDA: personal digital assistant
RR: relative risk
SMS: text message
WMD: weighted mean difference
