# Supplementary material for: The Effectiveness of Mobile-Health Technology-Based Health Behaviour Change or Disease Management Interventions for Health Care Consumers: A Systematic Review
Source: PLoS Med. 2013 Jan 15;10(1):e1001362. doi: 10.1371/journal.pmed.1001362 (PMC3548655; doi:10.1371/journal.pmed.1001362)
Supplement: Table S1 — Excluded studies. (DOCX) [file pmed.1001362.s001.docx]

**Table S1** Excluded Studies

| **Author** | **Reason for Exclusion** | **Protocol** | **Ongoing trial** | **Not an intervention trial** | **Not a trial** | **Not an MED** | **MED with another intervention** | **No relevant outcome** | **Study already included/ assessed** | **Full text not found** |
| --- | --- | --- | --- | --- | --- | --- | --- | --- | --- | --- |
| Adelaide Health Technology, A. | this is a summary paper on the use of sms messages to improve appointment attendance. It appears the referenced studies have already been screened | . | . | . | . | . | . | . | ✓ | . |
| Alhajji 2009^1^ | Not a controlled study | . | . | . | ✓ | . | . | . | . | . |
| Andrade 2005 ^2^ | Not an MED as defined in the protocol | . | . | . | . | ✓ | . | . | . | . |
| Annesi 1998 ^3^ | Not a mobile technology intervention. | . | . | . | . | ✓ | . | . | . | . |
| Anon 2008 ^4^ | Study has not finished yet | . | ✓ | . | . | . | . | . | . | . |
| Armstrong 2009 ^5^ | This is a conference presentation of paper 192 (also conference abstract 26254) | . | . | . | . | . | . | . | ✓ | . |
| Armstrong 2008 ^5^ | This is a conference abstract for study 192. | . | . | . | . | . | . | . | ✓ | . |
| Attof 2007 ^6^ | It is a before and after study. Data were collected on patient care outcomes during a period of usual practice for 3 months in 2005 then .the nutritional PDA was introduced in 2006 and further patient outcome data collected. | . | . | . | ✓ | . | . | . | . | . |
| Aziz 2005 ^7^ | The doctors had access to the PDA every alternate week. During weeks 1, 3 and 5, study participants adopted the conventional pager system for communication, and used PDAs during weeks 2, 4 and 6 - this is non-randomised and effectively a before and after study. | . | . | . | ✓ | . | . | . | . | . |
| Bacchus 1994 ^8^. | Quick Medical Reference is a computer program (determined by reading: CMAJ 1999;161(6):725-8 Effectiveness of the Quick Medical Reference as a diagnostic tool) | . | . | . | . | ✓ | . | . | . | . |
| Baker 2002 ^9^ | Not delivered using an MED | . | . | . | . | ✓ | . | . | . | . |
| Bakken 2006 ^10^ | This paper describes a device. | . | . | ✓ | . | . | . | . | . | . |
| Baranowski 2003 ^11^ | Not delivered on an MED | . | . | . | . | ✓ | . | . | . | . |
| Barry 1997 ^12^ | Intervention is not mobile. | . | . | . | . | ✓ | . | . | . | . |
| Bartholomew 2000 ^13^ | The intervention is not mobile. | . | . | . | . | ✓ | . | . | . | . |
| Bartley 2003 ^14^ | This is a conference presentation of 5363 | . | . | . | . | . | . | . | ✓ | . |
| Bartley 2004 ^15^ | This is not a controlled study so cannot be used to look at medication adherence. | . | . | . | ✓ | . | . | . | . | . |
| Basak 2008 ^16^ | The video game was played on a PC (not mobile). | . | . | . | . | ✓ | . | . | . | . |
| Bauer 2003 ^17^ | Pilot study, describing 2 patients in the intervention group only. | . | . | . | ✓ | . | . | . | . | . |
| Beale 2007 ^18^ | The intervention is provided on a "mini-PC" - study is similar to 1616 - where the intervention was on a PC. | . | . | . | . | ✓ | . | . | . | . |
| Bernabe-Ortiz 2008 ^19^ | research application only no health or health service outcome | . | . | ✓ | . | . | . | . | . | .. |
| Bird 2006 ^20^ | uses an historical control group . | . | . | . | ✓ | . | . | . | . | . |
| Blanchet 2009 ^21^ | This is a review article. | . | . | . | ✓ | . | . | . | . | . |
| Blive 2001^22^ | Not an MED | . | . | . | . | ✓ | . | . | . | . |
| Bollschweiler 2008 ^23^ | the intervention receives a Multimedia-Based Information Program (MM-IP) groups it does not appear to be delivered on a med. | . | . | . | . | ✓ | . | . | . | . |
| Bolten 1991 ^24^ | Not delivered on an MED | . | . | . | . | ✓ | . | . | . | . |
| Bower 2000 ^25^ | no control group | . | . | . | ✓ | . | . | . | . | . |
| Bramley 2005 ^26^ | This is a subgroup analysis of the trial presented in paper 4594 | . | . | . | . | . | . | . | ✓ | . |
| Breitfeld 1999 ^27^ | Pilot study with no controls. | . | . | . | ✓ | . | . | . | . | . |
| Brendryen 2008 ^28^ | The intervention is a web-based programme with SMS & mobile interactive voice response used only to monitor lapses/relapse after the 6 week web-based behaviour change programme. | . | . | . | . | ✓ | . | . | . | . |
| Brendryen 2006^29^ | The intervention is a web-based programme with SMS & mobile interactive voice response used only to monitor lapses/relapse after the 6 week web-based behaviour change programme. | . | . | . | . | ✓ | . | . | . | . |
| Brendryen 2008^30^ | The intervention is a web-based programme with SMS & mobile interactive voice response used only to monitor lapses/relapse after the 6 week web-based behaviour change programme. | . | . | . | . | . | . | ✓ | . | . |
| Bridges.Org 2003^31^ | Agreed to exclude because control and intervention groups are not concurrent | . | . | . | ✓ | . | . | . | . | . |
| Bridges.Org | Not a controlled study | . | . | . | ✓ | . | . | . | . | . |
| Broeren 2008 ^32^ | The intervention is a computer (non-mobile) game | . | . | . | . | ✓ | . | . | . | . |
| Brown 1997^33^ | The intervention is not mobile. | . | . | . | . | ✓ | . | . | . | . |
| Buitrago 2007 ^34^ | Paper could not be retrieved | . | . | . | . | . | . | . | . | ✓ |
| Bullard 2004^35^ | The mobile device is actually a laptop. | . | . | . | . | ✓ | . | . | . | . |
| Burayidi 2003^36^ | Not delivered on an MED | . | . | . | . | ✓ | . | . | . | . |
| Burke 2009^37^ | This is the protocol and baseline characteristics of the trial participants only. The trial is still ongoing | ✓ | . | . | . | . | . | . | . | . |
| Bushnell 2003^38^ | Not an MED. | . | . | . | . | ✓ | . | . | . | . |
| Cadario 2007^39^ | Not delivered on a MED. | . | . | . | . | ✓ | . | . | . | . |
| Cambpell 2009^40^ | The intervention is not mobile. | . | . | . | . | ✓ | . | . | . | . |
| Caro 2001^41^ | Study does not describe an MED. | . | . | . | . | ✓ | . | . | . | . |
| Chen 2005^42^ | This describes the development of a smart phone and the ease of use. | . | . | ✓ | . | . | . | . | . | . |
| Chib 2008^43^ | Computer games were played on a desktop computer. Confirmed by author through email correspondence. | . | . | . | . | ✓ | . | . | . | . |
| Chin 1995^44^ | Not MED or controlled study | . | . | . | . | ✓ | . | . | . | . |
| Cole 2006^45^ | Intervention and control MED completed concurrently | . | . | . | . | ✓ | . | . | . | . |
| Cole 2006^46^ | The intervention is a PC based game | . | . | . | . | ✓ | . | . | . | . |
| Collier 2005^47^ | Not a controlled study | . | . | . | ✓ | . | . | . | . | . |
| Collins | Research application only, no health or health service outcome. | . | . | ✓ | . | . | . | . | . | . |
| Coopmans 2005^48^ | This is a PhD thesis. The results are also in a paper which we have extracted: 2075_Coopmans. | . | . | . | . | . | . | . | ✓ | . |
| Crawford 2002^49^ | Questionnaire validation study, no health outcomes | . | . | ✓ | . | . | . | . | . | . |
| Creveling 1998^50^ | The intervention is not mobile (a computer game). | . | . | . | . | ✓ | . | . | . | . |
| Culley 2010^51^ | This is a before and after study. | . | . | . | ✓ | . | . | . | . | . |
| Curl 1994^52^ | This is a before and after study on a single ward | . | . | . | ✓ | . | . | . | . | . |
| Demiris 2007^53^ | Not a controlled study | . | . | . | ✓ | . | . | . | . | . |
| Depompei 2008^54^ | This is a before and after study, getting a reminder message was not random. | . | . | . | ✓ | . | . | . | . | . |
| Despont-Gros 2005^55^ | not an med as defined per protocol | . | . | . | . | ✓ | . | . | . | . |
| Donnelly 2008^56^ | There is no control group. | . | . | . | ✓ | . | . | . | . | . |
| Downer 2005^57^ | Cohort study with historical control (not concurrent) | . | . | . | ✓ | . | . | . | . | . |
| Downer 2006^58^ | It is a cohort study with historical control (not concurrent ) | . | . | . | ✓ | . | . | . | . | . |
| Drummond 1995^59^ | research application only no health or health service outcome | . | . | ✓ | . | . | . | . | . | . |
| Dykes 2006^60^ | Not a controlled study | . | . | . | ✓ | . | . | . | . | . |
| Elkin 2002^61^ | Not a trial of the MED - PDA only used to collect data in both intervention and comparison groups | . | . | . | . | ✓ | . | . | . | . |
| Enders 2003^62^ | The outcome is not a health outcome, there is no control group. | . | . | . | ✓ | . | . | . | , | . |
| Estellat 2008^63^ | not a med as defined by the protocol. The DP system comprises four components: the Hewlett Packard™ (HP) digital pen 200; CRF with paper printed (on HP color LaserJet printers) with a special pattern, based on dots slightly displaced from an orthogonal grid, unique for each page and each CRF (Anoto™technology); the software HPWorkflow Connect 200; and the Vision Objects™ Intelligent Character Recognition (ICR) system | . | . | . | . | ✓ | . | . | . | . |
| Finlay 2006^64^ | This study is testing the validity and reliability of a program but is not a trial | . | . | ✓ | . | . | . | . | . | . |
| Fjeldsoe 2010^65^ | As per protocol: there are other treatment differences between the treatment and control groups besides the delivery of the MED. | . | . | . | . | . | ✓ | . | . | . |
| Fletcher 2003^66^ | Not a health outcome | . | . | . | . | . | . | ✓ | . | . |
| Forster 1991^67^ | research application only no health or health service outcome | . | . | ✓ | . | . | . | . | . | . |
| Freynhagen 2006^68^ | Not a controlled study | . | . | . | ✓ | . | . | . | . | . |
| Galliher 2008^69^ | research application only no health or health service outcome | . | . | ✓ | . | . | . | . | . | . |
| Gorini 2009^70^ | This is a before and after study. | . | . | . | ✓ | . | . | . | . | . |
| Grad 2005^71^ | The intervention group in addition to the pda with its info retreiver software also receives an additional course EBM that the control group does not receive | . | . | . | . | ✓ | . | . | . | . |
| Grassi 2007^72^ | Duplicate study and results as paper 765 (which is included). | . | . | . | . | . | . | . | ✓ | . |
| Grechus 2000^73^ | Computer game not delivered on an MED | . | . | . | . | ✓ | . | . | . | . |
| Haller 2009^74^ | research application only no health or health service outcome | . | . | ✓ | . | . | . | . | . | . |
| Haynes 1998^75^ | Not a controlled study | . | . | . | ✓ | . | . | . | . | . |
| Hee-Sung 2007^76^ | The intervention involves use of internet with a phone or a wire connection. | . | . | . | . | ✓ | . | . | . | . |
| Heiberg 2007^77^ | Questionnaire validation study, no proximal health outcomes | . | . | ✓ | . | . | . | . | . | . |
| Helwig 1998^78^ | There is no control group. | . | . | . | ✓ | . | . | . | . | . |
| Hodgson 2005^79^ | Not a controlled study | . | . | . | ✓ | . | . | . | . | . |
| Hufford 2002^80^ | research application only no health or health service outcome | . | . | ✓ | . | . | . | . | . | . |
| Huss 2003^81^ | The intervention is a computer game (the paper makes no mention that the game is mobile). | . | . | . | . | ✓ | . | . | . | . |
| Hyland 1993^82^ | Intervention and control MED completed concurrently | . | . | . | . | . | . | . | . | . |
| Jamison 2002^83^ | Questionnaire validation study, no proximal health outcomes | . | . | ✓ | . | . | . | . | . | . |
| Jamison 2001^84^ | Questionnaire validation study, no proximal health outcomes | . | . | ✓ | . | . | . | . | . | . |
| Jaspan 2007^85^ | research application only no health or health service outcome | . | . | ✓ | . | . | . | . | . | . |
| Johnson 1993^86^ | Not delivered on MED | . | . | . | . | ✓ | . | . | . | . |
| Johnson 1995^87^ | Not delivered on MED | . | . | . | . | ✓ | . | . | . | . |
| Johnston 2004^88^ | There is no control group (all student had used the PDA). | . | . | . | ✓ | . | . | . | . | . |
| Joy 2003^89^ | No control group. | . | . | . | ✓ | . | . | . | . | . |
| Juniper 2009^90^ | Questionnaire validation study, no proximal health outcomes | . | . | ✓ | . | . | . | . | . | . |
| Junker 2008^91^ | Questionnaire validation study, no proximal health outcomes | . | . | ✓ | . | . | . | . | . | . |
| Kato 2008^92^ | The intervention is a PC game. | . | . | . | . | ✓ | . | . | . | . |
| Kempf 2005^93^ | No control group. This is just a report of a survey conducted with primary care physicians using a PDA. | . | . | . | ✓ | . | . | . | . | . |
| Keshavjee 2003^94^ | This paper describes the reasons for the failure of a trial with a PDA intervention. | . | . | ✓ | . | . | . | . | . | . |
| Kim 2007^95^ | The intervention involved either a cellular phone or personal computer | . | . | . | . | ✓ | . | . | . | . |
| Kim 2007^96^ | Internet access was possible by cellular phone or wired Internet | . | . | . | . | ✓ | . | . | . | . |
| Kim 2008^97^ | The intervention was done by either a cellular phone or wire Internet (non-mobile). | . | . | . | . | ✓ | . | . | . | . |
| Kim 2005^98^ | Intervention involved either a cellular phone or wire Internet (non-mobile). | . | . | . | . | ✓ | . | . | . | . |
| Kim 2008^99^ | The intervention involved either a phone or wired internet (non-mobile). | . | . | . | . | ✓ | . | . | . | . |
| Koff 2006^100^ | The intervention is not mobile. | . | . | . | . | ✓ | . | . | . | . |
| Kozijavkin 2004^101^ | The intervention (computer game) is not mobile. | . | . | . | . | ✓ | . | . | . | . |
| Kramer 2000^102^ | Not a MED. Device too large for an 'ultra portable' computer - 290 x 226 x 25 millimetres and 1451 g in weight. | . | . | . | . | ✓ | . | . | . | . |
| Kreindlerv 2003^103^ | No health outcomes. | . | . | . | . | . | . | ✓ | . | . |
| Krogh 2008^104^ | neither device is an MED the smallest is the The Toshiba Portégé M200 which while a tablet pc I do not regard as ultra portable given that the screen alone is 12 inches and it weight around 4.6 lb. | . | . | . | . | ✓ | . | . | . | . |
| Kurth 2002^105^ | Uses an historic control group - not concurrent | . | . | . | ✓ | . | . | . | . | . |
| Kuruvilla ^106^ | Questionnaire validation study, no proximal health outcomes | . | . | ✓ | . | . | . | . | . | . |
| Kvien 2005^107^ | Questionnaire validation study, no proximal health outcomes | . | . | ✓ | . | . | . | . | . | . |
| Labkoff 1995^108^ | There was no control group (only studied the residents who had PDA's). | . | . | . | ✓ | . | . | . | . | . |
| Larkin 2001^109^ | Not a controlled study. | . | . | . | ✓ | . | . | . | . | . |
| Lauritsen 2004^110^ | research application only no health or health service outcome | . | . | ✓ | . | . | . | . | . | . |
| Ledger 2008^111^ | This is a study protocol. | . | . | . | ✓ | . | . | . | . | . |
| Lefever 2008^112^ | Questionnaire validation study, no proximal health outcomes | . | . | ✓ | . | . | . | . | . | . |
| Leijdekkers 2009^113^ | This describes a trial, preliminary data only. No control group information. | . | . | . | ✓ | . | . | . | . | . |
| Lester 2009^114^ | Trial protocol | ✓ | . | . | . | . | . | . | . | . |
| Maguire 2007^115^ | Conference presentation of 862 | . | . | . | . | . | . | . | ✓ | . |
| Main 2004^116^ | Not a controlled study | . | . | . | ✓ | . | . | . | . | . |
| Marceau 2007^117^ | Not a controlled study | . | . | . | ✓ | . | . | . | . | . |
| Matthew 2007^118^ | Questionnaire validation study, no proximal health outcomes | . | . | ✓ | . | . | . | . | . | . |
| McBride 1999^119^ | Questionnaire validation study, no proximal health outcomes | . | . | ✓ | . | . | . | . | . | . |
| McCann 2009^120^ | Qualitative study of patient experiences of using MED - main study results reported in 862 | . | . | ✓ | . | . | . | . | . | . |
| McClung 2009^121^ | Questionnaire validation study, no proximal health outcomes | . | . | ✓ | . | . | . | . | . | . |
| McCormack 2009^122^ | Exergaming is not carried out on a handheld video game console. Confirmed by author through email correspondence. | . | . | . | . | ✓ | . | . | . | . |
| McDoniel 2009^123^ | Not deilvered on MED. The handheld device referred to in the title is used to estimate calorie balance in patients prior to randomisation but is not being evaluated in the trial. | . | . | . | . | ✓ | . | . | . | . |
| Michalowski 2004^124^ | Descriptive paper. | . | . | ✓ | . | . | . | . | . | . |
| Mignogna 2007^125^ | This is a conference abstract for presentation of the protocol for the trial. | ✓ | . | . | . | . | . | . | . | . |
| Miskelly 2005^126^ | There is no control group. | . | . | . | ✓ | . | . | . | . | . |
| Morgan 2007^127^ | research application only no health or health service outcome | . | . | ✓ | . | . | . | . | . | . |
| Mosso 2008^128^ | Duplicate publication of 732 | . | . | . | . | . | . | . | ✓ | . |
| Murray 2003^129^ | Not a controlled study | . | . | . | ✓ | . | . | . | . | . |
| Neville 2002^130^ | Pilot study, no control group. | . | . | . | ✓ | . | . | . | . | . |
| Newell 2001^131^ | Not a controlled study | . | . | . | ✓ | . | . | . | . | . |
| Newman 1997^132^ | Exclude on the basis that the control gets an MED (1 component) + treatment, whereas the intervention gets MED (2 components) | . | . | . | ✓ | . | . | . | . | . |
| Nguyen 2008^133^ | The intervention participants could use either the PDA or a website (accessed on a non-mobile computer) to enter data. | . | . | . | . | ✓ | . | . | . | . |
| Nguyen 2006^134^ | Not a controlled study | . | . | . | ✓ | . | . | . | . | . |
| Nyholm 2004^135^ | case studies so no control group | . | . | . | ✓ | . | . | . | . | . |
| Palen 2008^136^ | No health outcomes measured. Control and intervention groups measured 1 year apart. | . | . | . | . | . | . | ✓ | . | . |
| Park 2009^137^ | The intervention includes a internet/computer usage component. | . | . | . | . | ✓ | . | . | . | . |
| Patnaik 2009^138^ | research application only no health or health service outcome | . | . | ✓ | . | . | . | . | . | . |
| Peng 2009^139^ | The paper does not specifically mention any MED in the description of the game deliver by computer | . | . | . | . | ✓ | . | . | . | . |
| Perkins 2006^140^ | The outcome is not a health outcome, there is no control group. | . | . | . | ✓ | . | . | . | . | . |
| Prestwich 2008^141^ | This is a conference presentation of the published paper 13973 | . | . | . | . | . | . | . | ✓ | . |
| Pulley 2002^142^ | Not delivered on MED | . | . | . | ✓ | . | . | . | . | . |
| Quinn 2009^143^ | This is a study protocol | ✓ | . | . | . | . | . | . | . | . |
| Raisch 2003^144^ | Not a controlled study | . | . | . | ✓ | . | . | . | . | . |
| Reynolds 2007^145^ | This is a descriptive paper/literature review. | . | . | ✓ | . | . | . | . | . | . |
| Rezaiyan 2007^146^ | The intervention (computer game) is not mobile. | . | . | . | . | ✓ | . | . | . | . |
| Richter 2008^147^ | Questionnaire validation study, no proximal health outcomes | . | . | ✓ | . | . | . | . | . | . |
| Ring 2008^148^ | Questionnaire validation study, no proximal health outcomes | . | . | ✓ | . | . | . | . | . | . |
| Rivellese 1991^149^ | Not an MED | . | . | . | . | ✓ | . | . | . | . |
| Rivera 2008^150^ | research application only no health or health service outcome | . | . | ✓ | . | . | . | . | . | . |
| Rosenberger 2009^151^ | This is a descriptive paper of an intervention trial. | . | . | . | ✓ | . | . | . | . | . |
| Rowan 2007^152^ | research application only no health or health service outcome | . | . | ✓ | . | . | . | . | . | . |
| Ruder 2007^153^ | Not a controlled study | . | . | . | ✓ | . | . | . | . | . |
| Ruland 2002^154^ | No proper control group: both inyervention and control have exposure to the same PDA program. | . | . | . | ✓ | . | . | . | . | . |
| Ruland 2003^155^ | Trial of a non-MED component that differs between the groups. The controls and interventions are not from the same time frame. | . | . | . | . | ✓ | . | . | . | . |
| Russoniello 2009^156^ | Not an MED | . | . | . | . | ✓ | . | . | . | . |
| Ryan 2009^157^. | This is a study protocol. | . | . | . | ✓ | . | . | . | . | . |
| Ryuzaki 2008^158^ | There is no control group. | . | . | . | ✓ | . | . | . | . | . |
| Saleh 2002^159^ | Questionnaire validation study, no proximal health outcomes | . | . | ✓ | . | . | . | . | . | . |
| Samore 2005^160^ | The intervention comprised decision support tools on paper and a handheld computer. It was not possible to separate these two. | . | . | . | . | ✓ | . | . | . | . |
| Sawa 2003^161^ | 1. It is a pilot study with 2 participants (the residents) 2. The completion of the examinations with and without PDA are done one directly after the other i.e. concurrently | . | . | . | ✓ | . | . | . | . | . |
| Schlickum 2009^162^ | Not an MED | . | . | . | . | ✓ | . | . | . | . |
| Seebregts 2009^163^ | Questionnaire validation study, no proximal health outcomes | . | . | ✓ | . | . | . | . | . | . |
| Sellors 2002^164^ | Not an MED | . | . | . | . | ✓ | . | . | . | . |
| Sevick 2008^165^ | The intervention was a PDA+behaviour change meetings. | . | . | . | . | . | ✓ | . | . | . |
| Shea 2004^166^ | Not and MED. Not a controlled study | . | . | . | . | ✓ | . | . | . | . |
| Shelby-James 2007^167^ | Intervention and control MED completed concurrently | . | . | . | ✓ | . | . | . | . | . |
| Silvey 2005^168^ | No health outcomes | . | . | . | . | . | . | ✓ | . | . |
| Snooks 2010^169^ | Protocol - added to ongoing studies list | ✓ | . | . | . | . | . | . | . | . |
| Stack Jr 2003^170^ | Not a controlled study | . | . | . | ✓ | . | . | . | . | . |
| Standen 2006^171^ | Abstract for a conference presentation. Does not explicitly mention any handheld video game console. | . | . | . | . | ✓ | . | . | . | . |
| Staresinic 2002^172^ | Not a controlled study of the MED. | . | . | . | ✓ | . | . | . | . | . |
| Sterling 2003^173^ | a portable pc is used not a MED as defined by protocol | . | . | . | . | ✓ | . | . | . | . |
| Stern 2007^174^ | Trial still ongoing |  |  |  |  |  |  |  |  |  |
| Stone 1998^175^ | research application only no health or health service outcome | . | . | ✓ | . | . | . | . | . | . |
| Strandbygaard | Study already included  . | . | . | . | . | . | . | . | ✓ | . |
| Stratton 1998^176^ | Questionnaire validation study, no proximal health outcomes | . | . | ✓ | . | . | . | . | . | . |
| Tegang 2009^177^ | No health outcomes measured. | . | . | . | . | . | . | ✓ | . | . |
| Trapl 2005^178^ | Not a controlled study | . | . | . | ✓ | . | . | . | . | . |
| Tseng 1998^179^ | Not a health outcome | . | . | . | . | . | . | ✓ | . | . |
| Turnin 2000^180^ | Intervention not delivered on an MED | . | . | . | . | ✓ | . | . | . | . |
| Turnin 1998^181^ | Abstract for a conference presentation. Does not mention an MED with the description of the computer game. | . | . | . | . | ✓ | . | . | . | . |
| Turnin 2001^182^ | Duplicate of 24182 | . | . | . | . | . | . | . | ✓ | . |
| Van Griensven 2006^183^ | research application only no health or health service outcome | . | . | ✓ | . | . | . | . | . | . |
| Vandenkerkhof 2003^184^ | Before and after pilot study (n=1) | . | . | . | ✓ | . | . | . | . | . |
| Vasterling 1993^185^ | The video game intervention is on a non-mobile PC (see page 70). | . | . | . | . | ✓ | . | . | . | . |
| Vidrine 2006^186^ | Already included | . | . | . | . | . | . | . | ✓ | . |
| Walters 2010^187^ | Protocol - added to ongoing study list | ✓ | . | . | . | . | . | . | . | . |
| Wang 2002^188^ | Questionnaire validation study, no proximal health outcomes | . | . | ✓ | . | . | . | . | . | . |
| Wetter 2006^189^ | Three trials mentioned in the abstract but no data provided, 1 was ongoing at the time of writing. |  |  |  |  |  |  |  |  |  |
| Woods 2006^190^ | Not a controlled trial of the MED. MED used to collect data for a trial from all treatment groups. | . | . | . | ✓ | . | . | . | . | . |
| Wright 2008^191^ | not an MED | . | . | . | . | ✓ | . | . | . | . |
| Wright 2001^192^ | No health outcomes | . | . | . | . | . | . | ✓ | . | . |
| Wright 2001^193^ | The experiment is participants using (a) a pocket computer with a physical keyboard or (b) a pocket computer with a touch screen keyboard. | . | . | . | . | ✓ | . | . | . | . |
| Yen 2005^194^ | it is not MED as defined per protocol. The intervention in this study was the useof a digital pen and paper system (io Digital Pen, Logitech, Inc). | . | . | . | . | ✓ | . | . | . | . |
| Yoon 2008^195^ | The intervention is wired internet or a cell-phone. | . | . | . | . | ✓ | . | . | . | . |
| Young 2001^196^ | Only reports participant perceptions regarding use , accuracy and speed , no objective primary outcomes. | . | . | . | . | . | . | ✓ | . | . |
| Zhao 2006^197^ | This is mainly a descriptive paper of a before and after study | . | . | ✓ | . | . | . | . | . | . |
| Zwarenstein 2007^198^ | Protocol paper | ✓ | . | . | ✓ | . | . | . | . | . |

1. Alhajji, M., A. Jeffrey, and A. Datta, *Tablet Pc to Evaluate Respiratory Patient Preference and Satisfaction Using the 18-Element Consultation Specific Questionnaire.* Thorax, 2009. **64**: p. A90-A90.

2. Andrade, A.S., et al., *A programmable prompting device improves adherence to highly active antiretroviral therapy in HIV-infected subjects with memory impairment.* Clin Infect Dis, 2005. **41**(6): p. 875-82.

3. Annesi, J.J., *Effects of computer feedback on adherence to exercise.* Perceptual & Motor Skills, 1998. **87**(2): p. 723-30.

4. Anon (2008) *[Public title] "NeuroCue", a randomized controlled study into the use of an electronic cognitive aid in patients with acquired brain injury; [Official title] "NeuroCue", a randomized controlled study into the effectiveness and usability of a Personal Digital Assistant (PDA) used by patients with acquired brain injury*. NeuroCue//NTR1219//Trial web site:<http://wwwnpunimaasnl/neurocue> **Volume**,

5. Armstrong, A., et al., *A randomized, controlled trial evaluating adherence to sunscreen using electronic monitoring and text message reminders.* Journal of the American Academy of Dermatology, 2009. **60**(3): p. AB88-AB88.

6. Attof, Y., et al., *[From the creation to the appreciation of a personal digital assistant-based clinical decision-support system for the management of artificial nutrition].* Annales Francaises d Anesthesie et de Reanimation, 2007. **26**(12): p. 1031-6.

7. Aziz, O., et al., *Handheld computers and the 21st century surgical team: a pilot study.* BMC Medical Informatics & Decision Making, 2005. **5**: p. 28.

8. Bacchus, C.M., et al., *A randomized crossover trial of Quick Medical Reference (QMR) as a teaching tool for medical interns.* Journal of General Internal Medicine, 1994. **9**(11): p. 616-21.

9. Baker, A.H. and J. Wardle (2002) *Increasing fruit and vegetable intake among adults attending colorectal cancer screening: the efficacy of a brief tailored intervention*. Cancer epidemiology, biomarkers & prevention : a publication of the American Association for Cancer Research, cosponsored by the American Society of Preventive Oncology **Volume**, 203-6

10. Bakken, S., et al., *Mobile decision support for advanced practice nurses.* Studies in Health Technology & Informatics, 2006. **122**: p. 1002.

11. Baranowski, T., et al., *Squire's Quest!: Dietary outcome evaluation of a multimedia game.* American Journal of Preventive Medicine, 2003. **24**(1): p. 52-61.

12. Barry, M.J., et al., *A randomized trial of a multimedia shared decision-making program for men facing a treatment decision for benign prostatic hyperplasia.* Disease Management and Clinical Outcomes, 1997. **1**(1): p. 5-14.

13. Bartholomew, L., et al., *Watch, Discover, Think, and Act: Evaluation of computer-assisted instruction to improve asthma self-management in inner-city children.* Patient Education and Counseling, 2000. **39**(2-3): p. 269-280.

14. Bartley, J., et al. (2003) *Therapeutic compliance in treating women with bacterial vaginosis using oral or vaginal metronidazole documented by personal digital assistant (PDA) technology*. International Journal of Obstetrics & Gynecology **Volume**, 44

15. Bartley, J.B., et al., *Personal digital assistants used to document compliance of bacterial vaginosis treatment.* Sexually Transmitted Diseases, 2004. **31**(8): p. 488-91.

16. Basak, C., et al., *Can training in a real-time strategy video game attenuate cognitive decline in older adults?* Psychology & Aging, 2008. **23**(4): p. 765-77.

17. Bauer, S., et al., *Use of text messaging in the aftercare of patients with bulimia nervosa.* European Eating Disorders Review, 2003. **11**(3): p. 279-290.

18. Beale, I.L., et al., *Improvement in cancer-related knowledge following use of a psychoeducational video game for adolescents and young adults with cancer.* Journal of Adolescent Health, 2007. **41**(3): p. 263-70.

19. Bernabe-Ortiz, A., et al., *Handheld computers for self-administered sensitive data collection: a comparative study in Peru.* BMC Med Inform Decis Mak, 2008. **8**: p. 11.

20. Bird, S.B. and D.R. Lane, *House officer procedure documentation using a Personal Digital Assistant: a longitudinal study.* BMC Medical Informatics & Decision Making, 2006. **6**: p. 5.

21. Blanchet, K.D., *Remote monitoring of asthma.* Telemedicine and e Health, 2009. **15**(3): p. 227-230.

22. Bliven, B.D., S.E. Kaufman, and J.A. Spertus, *Electronic collection of health-related quality of life data: validity, time benefits, and patient preference.* Qual Life Res, 2001. **10**(1): p. 15-22.

23. Bollschweiler, E., et al. (2008) *Improving informed consent of surgical patients using a multimedia-based program? Results of a prospective randomized multicenter study of patients before cholecystectomy*. Annals of Surgery **Volume**, 205-11

24. Bolten, W., et al., *[Validation of electronic by conventional pain diaries].* Zeitschrift fur Rheumatologie, 1991. **50 Suppl 1**: p. 55-64.

25. Bower, D.J. and C.J. Bertling, *Using PalmPilots as a teaching tool during a primary care clerkship. Advanced Education Group.* Acad Med, 2000. **75**(5): p. 541-2.

26. Bramley, D., et al., *Smoking cessation using mobile phone text messaging is as effective in Maori as non-Maori.* New Zealand Medical Journal, 2005. **118**(1216).

27. Breitfeld, P.P., et al., *Pilot study of a point-of-use decision support tool for cancer clinical trials eligibility.* Journal of the American Medical Informatics Association, 1999. **6**(6): p. 466-77.

28. Brendryen, H., F. Drozd, and P. Kraft, *A digital smoking cessation program delivered through internet and cell phone without nicotine replacement (happy ending): randomized controlled trial.* Journal of Medical Internet Research, 2008. **10**(5): p. e51.

29. Brendryen, H. and P. Kraft, *A RCT of an internet and cell-phone based smoking cessation intervention.* Psychology & Health, 2006. **21**: p. 23-24.

30. Brendryen, H. and P. Kraft, *Happy ending: a randomized controlled trial of a digital multi-media smoking cessation intervention.* Addiction, 2008. **103**(3): p. 478-84; discussion 485-6.

31. bridges.org, *Evaluation of the SATELLIFE PDA Project, 2002: Testing the use of handheld computers for heathcare in Ghana, Uganda, and Kenya*. 2003.

32. Broeren, J., et al. (2008) *The possibilities of VR and computer games in an activity center for community dwelling persons with stroke*. International Journal of Stroke **Volume**, 354

33. Brown, S.J., et al., *Educational video game for juvenile diabetes: results of a controlled trial.* Medical Informatics, 1997. **22**(1): p. 77-89.

34. Buitrago, F., *Mobile texts and calls improve the assistance in primary care consultations. [Spanish].* FMC Formacion Medica Continuada en Atencion Primaria, 2007. **14**(9).

35. Bullard, M.J., et al., *Supporting clinical practice at the bedside using wireless technology.* Academic Emergency Medicine, 2004. **11**(11): p. 1186-92.

36. Burayidi, M.A., *The use of electronic messages to promote seat belt use. Report of a pilot study in Wisconsin.* Injury Control & Safety Promotion, 2003. **10**(4): p. 257-60.

37. Burke, L.E., et al., *SMART trial: A randomized clinical trial of self-monitoring in behavioral weight management-design and baseline findings.* Contemporary Clinical Trials, 2009. **30**(6): p. 540-51.

38. Bushnell, D.M., M.L. Martin, and B. Parasuraman, *Electronic versus paper questionnaires: a further comparison in persons with asthma.* J Asthma, 2003. **40**(7): p. 751-62.

39. Cadario, F., et al., *Telecare for teenagers with type 1 diabetes: a trial.* Minerva Pediatrica, 2007. **59**(4): p. 299-305.

40. Cambpell, T.C., et al., *Impact of the comprehensive health enhancement support system (CHESS), an interactive cancer communication system (ICCS) on NSCLC survival: a randomized study comparing CHESS with the internet.* Journal of Thoracic Oncology, 2009. **4**(9): p. S369-S370.

41. Caro, J.J., Sr., et al., *Does electronic implementation of questionnaires used in asthma alter responses compared to paper implementation?* Qual Life Res, 2001. **10**(8): p. 683-91.

42. Chen, M.J., et al., *The development of smart-phone-based home care evaluation support system.* Healthcom 2005: 7th International Workshop on Enterprise Networking and Computing in Healthcare Industry, Proceedings, 2005: p. 267-268.

43. Chib, A.I., *Network influences in health initiatives: Multimedia games for youth in Peru*. 2008, Chib, Arul I : U Southern California, US.

44. Chin, K., et al., *Intellectual work using a video game inhibits post hyperventilation hyperpnoea following voluntary hyperventilation while it stimulates breathing at rest.* Advances in Experimental Medicine and Biology, 1995. **393**: p. 81-84.

45. Cole, E., et al., *A comparative study of mobile electronic data entry systems for clinical trials data collection.* International Journal of Medical Informatics, 2006. **75**(10-11): p. 722-9.

46. Cole, S.W., et al., *Clinical trial of Re-Mission: A video game for young people with cancer.* Cyberpsychology & Behavior, 2006. **9**(6): p. 665-666.

47. Collier, J.M. and J. Bernhardt (2005) *Clinical trial data collection using a personal digital assistant*. Internal Medicine Journal **Volume**, A17

48. Coopmans, V.C., *Certified registered nurse anesthetist performance and perceptions: Use of a handheld, computerized, decision making aid during critical events in a high-fidelity human simulation environment*. 2005, Coopmans, Vicki C : Virginia Commonwealth U , US.

49. Crawford, E., et al., *The evaluation of a hand-held computerised visual analogue scale rating system for appetite.* Proceedings of the Nutrition Society, 2002. **61**(OCB): p. 164A.

50. Creveling, C.C., et al., *A video game computer sub-program to modify the disinhibition process in ADHD adolescents: Part II. Experimental Group II and the sub-program.* Archives of Clinical Neuropsychology, 1998. **13**(1): p. 69-70.

51. Culley, C. and J.J. Evans, *SMS text messaging as a means of increasing recall of therapy goals in brain injury rehabilitation: A single-blind within-subjects trial.* Neuropsychological Rehabilitation, 2010. **20**(1): p. 103-119.

52. Curl, M. and D. Robinson, *Hand-held computers in clinical audit: a comparison with established paper and pencil methods.* Int J Health Care Qual Assur, 1994. **7**(3): p. 16-20.

53. Demiris, G., et al., *Telehospice tools for caregivers: A pilot study.* Clinical Gerontologist, 2007. **31**(1): p. 43-57.

54. Depompei, R., et al., *Practical applications for use of PDAs and smartphones with children and adolescents who have traumatic brain injury.* Neurorehabilitation, 2008. **23**(6): p. 487-99.

55. Despont-Gros, C., et al., *The digital pen and paper: Evaluation and acceptance of a new data acquisition device in clinical settings.* Methods of Information in Medicine, 2005. **44**(3): p. 359-368.

56. Donnelly, M.P., et al., *Development of a cell phone-based video streaming system for persons with early stage Alzheimer's disease.* Conference Proceedings: ... Annual International Conference of the IEEE Engineering in Medicine & Biology Society, 2008. **2008**: p. 5330-3.

57. Downer, S.R., J.G. Meara, and A.C. Da Costa, *Use of SMS text messaging to improve outpatient attendance.* Medical Journal of Australia, 2005. **183**(7): p. 366-8.

58. Downer, S.R., et al., *SMS text messaging improves outpatient attendance.* Australian Health Review, 2006. **30**(3): p. 389-96.

59. Drummond, H.E., et al., *Electronic quality of life questionnaires: a comparison of pen-based electronic questionnaires with conventional paper in a gastrointestinal study.* Qual Life Res, 1995. **4**(1): p. 21-6.

60. Dykes, P.C., et al., *The feasibility of digital pen and paper technology for vital sign data capture in acute care settings.* AMIA .. 2006. **Annual Symposium Proceedings/AMIA Symposium.**: p. 229-33.

61. Elkin, D., et al., *Influence of hospitalists on patient spectrum and length of stay on inpatient teaching service: A Palm Pilot-based study.* Journal of General Internal Medicine, 2002. **17**: p. 93-93.

62. Enders, S.J. and J.M. Enders, *Is the information there? Evaluation of personal digital assistant drug information software programs for anticoagulant and antithrombotic therapies.* Pharmacotherapy, 2003. **23**(10): p. 1334-1335.

63. Estellat, C., et al., *Data capture by digital pen in clinical trials: a qualitative and quantitative study.* Contemporary Clinical Trials, 2008. **29**(3): p. 314-23.

64. Finlay, K., et al., *In-training evaluation using hand-held computerized clinical work sampling strategies in radiology residency.* Canadian Association of Radiologists Journal-Journal De L Association Canadienne Des Radiologistes, 2006. **57**(4): p. 232-237.

65. Fjeldsoe, B.S., Y.D. Miller, and A.L. Marshall, *MobileMums: a randomized controlled trial of an SMS-based physical activity intervention.* Annals of Behavioral Medicine, 2010. **39**(2): p. 101-11.

66. Fletcher, L.A., et al., *Handheld computers. A feasible alternative to paper forms for field data collection.* Eval Rev, 2003. **27**(2): p. 165-78.

67. Forster, D., et al., *Evaluation of a computerized field data collection system for health surveys.* Bull World Health Organ, 1991. **69**(1): p. 107-11.

68. Freynhagen, R., et al., *Screening of neuropathic pain components in patients with chronic back pain associated with nerve root compression: A prospective observational pilot study (MIPORT).* Current Medical Research and Opinion, 2006. **22**(3): p. 529-537.

69. Galliher, J.M., et al., *Data collection outcomes comparing paper forms with PDA forms in an office-based patient survey.* Annals of Family Medicine, 2008. **6**(2): p. 154-60.

70. Gorini, A., et al., *A Portable Virtual Reality System as an Alternative Medical Treatment to Reduce Pain-Related Anxiety in Ambulatory Surgical Operations: A Randomized Controlled Study.* Cyberpsychology & Behavior, 2009. **12**(5): p. 613-613.

71. Grad, R.M., et al., *Effect of a PDA-assisted evidence-based medicine course on knowledge of common clinical problems.* Family Medicine, 2005. **37**(10): p. 734-40.

72. Grassi, A., et al., *A relaxing journey: The use of mobile phones for well-being improvement.* Annual Review of CyberTherapy and Telemedicine, 2007. **5**: p. 123-131.

73. Grechus, M. and J. Brown (2000) *Comparison of individulized computer game reinforcement versus peer-interactive board game reinforcement on retention of nutrition label knowledge*. Journal of Health Education **Volume**, 138-142

74. Haller, D.M., et al., *Text message communication in primary care research: a randomized controlled trial.* Family Practice, 2009. **26**(4): p. 325-30.

75. Haynes, G., F. Overdyk, and P. Arvanitis, *Portable data acquisition combined with Web based data access improves preoperative evaluations without compromising confidentiality.* Anesthesiology (Hagerstown), 1998. **89**(3A): p. A1348.

76. Hee-Sung, K., *Impact of Web-based nurse's education on glycosylated haemoglobin in type 2 diabetic patients.* Journal of Clinical Nursing, 2007. **16**(7): p. 1361-6.

77. Heiberg, T., et al., *Daily health status registration (patient diary) in patients with rheumatoid arthritis: A comparison between personal digital assistant and paper-pencil format.* Arthritis Care and Research, 2007. **57**(3): p. 454-460.

78. Helwig, A.L. and C. Flynn, *Using palm-top computers to improve students' evidence-based decision making.* Acad Med, 1998. **73**(5): p. 603-4.

79. Hodgson, Y., *Short Message Service as A Support Tool In Medication Adherence And Chronic Disease Management.* Health Care and Information Review Online, 2005. **9**(3).

80. Hufford, M.R., *Paper vs. Electronic Diaries.* Applied Clinical Trials, 2002. **11**: p. 6.

81. Huss, K., et al., *Computer game for inner-city children does not improve asthma outcomes.* Journal of Pediatric Health Care, 2003. **17**(2): p. 72-8.

82. Hyland, M.E., et al., *Diary keeping in asthma: comparison of written and electronic methods.* BMJ, 1993. **306**(6876): p. 487-9.

83. Jamison, R.N., et al., *Comparative study of electronic vs. paper VAS ratings: a randomized, crossover trial using healthy volunteers.* Pain, 2002. **99**(1-2): p. 341-7.

84. Jamison, R.N., et al., *Electronic diaries for monitoring chronic pain: 1-year validation study.* Pain, 2001. **91**(3): p. 277-85.

85. Jaspan, H.B., et al., *Brief report: Methods for collecting sexual behaviour information from South African adolescents - a comparison of paper versus personal digital assistant questionnaires.* Journal of Adolescence, 2007. **30**(2): p. 353-359.

86. Johnson, F.E., et al., *Evaluation of an electronic message strip to recruit smokers to a smoking cessation program.* J Cancer Educ, 1993. **8**(1): p. 79-83.

87. Johnson, F.E., et al., *Evaluation of alternate messages on an electronic message strip to recruit subjects to a smoking-cessation program.* Journal of Cancer Education, 1995. **10**(1): p. 31-33.

88. Johnston, J.M., et al., *Evaluation of a handheld clinical decision support tool for evidence-based learning and practice in medical undergraduates.* Medical Education, 2004. **38**(6): p. 628-37.

89. Joy, S.V. and J.A. Gouveia-Pisano, *The use of personal digital assistants (PDA's) to improve the detection, evaluation and discussion of men's health issues in a primary care setting.* Journal of General Internal Medicine, 2003. **18**: p. 133-133.

90. Juniper, E.F., J.M. Langlands, and B.A. Juniper (2009) *Patients may respond differently to paper and electronic versions of the same questionnaires*. Respiratory Medicine **Volume**, 932-4

91. Junker, U., et al., *Paper versus electronic rating scales for pain assessment: a prospective, randomised, cross-over validation study with 200 chronic pain patients.* Current Medical Research & Opinion, 2008. **24**(6): p. 1797-806.

92. Kato, P.M., et al., *A video game improves behavioral outcomes in adolescents and young adults with cancer: a randomized trial.* Pediatrics, 2008. **122**(2): p. e305-17.

93. Kempf, C., et al., *[Data recording using a personal digital assistant. Experience of prospective survey on pain in 3196 patients].* Presse Medicale, 2005. **34**(5): p. 343-7.

94. Keshavjee, K., et al., *Technology failure analysis: understanding why a diabetes management tool developed for a Personal Digital Assistant (PDA) didn't work in a randomized controlled trial.* AMIA .. 2003. **Annual Symposium Proceedings/AMIA Symposium.**: p. 889.

95. Kim, H.-S., *A randomized controlled trial of a nurse short-message service by cellular phone for people with diabetes.* International Journal of Nursing Studies, 2007. **44**(5): p. 687-92.

96. Kim, H.-S. and H.-S. Jeong, *A nurse short message service by cellular phone in type-2 diabetic patients for six months.* Journal of Clinical Nursing, 2007. **16**(6): p. 1082-7.

97. Kim, H.-S. and M.-S. Song, *Technological intervention for obese patients with type 2 diabetes.* Applied Nursing Research, 2008. **21**(2): p. 84-9.

98. Kim, H.-S., Y.-S. Yoo, and H.-S. Shim, *Effects of an Internet-based intervention on plasma glucose levels in patients with type 2 diabetes.* Journal of Nursing Care Quality, 2005. **20**(4): p. 335-40.

99. Kim, S.-I. and H.-S. Kim, *Effectiveness of mobile and internet intervention in patients with obese type 2 diabetes.* International Journal of Medical Informatics, 2008. **77**(6): p. 399-404.

100. Koff, P.B., et al. (2006) *Telemonitoring/ehealth management improves quality of life and healthcare expenditures in COPD [Abstract]*. Proceedings of the American Thoracic Society **Volume**, A123 [Poster J87]

101. Kozijavkin, V. and O. Kachmar (2004) *The use of computer games in a post-stroke patients' late rehabilitation. A pilot study*. Stroke **Volume**, e314

102. Kramer, M., et al., *Evaluation of the effect mobile computers on physician acceptance of an online order entry system.* Journal of the American Medical Informatics Association, 2000: p. 1051-1051.

103. Kreindler, D., et al., *Portable mood mapping: the validity and reliability of analog scale displays for mood assessment via hand-held computer.* Psychiatry Research, 2003. **120**(2): p. 165-77.

104. Krogh, P.R., S. Rough, and S. Thomley, *Comparison of two personal-computer-based mobile devices to support pharmacists' clinical documentation.* American Journal of Health-System Pharmacy, 2008. **65**(2): p. 154-157.

105. Kurth, R.J., V. Silenzio, and M.M. Irigoyen, *Use of personal digital assistants to enhance educational evaluation in a primary care clerkship.* Medical Teacher, 2002. **24**(5): p. 488-490.

106. Kuruvilla, P.G., et al., *Assessing quality of life (QL) and patient reported outcomes (PROs) in clinical trials and clinical practice: A study using a hand-held computerized form of the validated LCSS instrument in patients with non-small cell lung cancer (NSCLC).* Journal of Clinical Oncology, 2005. **23**(16): p. 745S-745S.

107. Kvien, T.K., et al., *Performance of health status measures with a pen based personal digital assistant.* Ann Rheum Dis, 2005. **64**(10): p. 1480-4.

108. Labkoff, S.E., et al., *The Constellation Project: experience and evaluation of personal digital assistants in the clinical environment.* Proc Annu Symp Comput Appl Med Care, 1995: p. 678-82.

109. Larkin, M., *Can handheld computers improve the quality of care?* Lancet, 2001. **358**(9291): p. 1438.

110. Lauritsen, K., et al., *Symptom recording in a randomised clinical trial: paper diaries vs. electronic or telephone data capture.* Control Clin Trials, 2004. **25**(6): p. 585-97.

111. Ledger, S., et al., *A randomised controlled trial evaluating the effect of an individual auditory cueing device on freezing and gait speed in people with Parkinson's disease.* BMC Neurology, 2008. **8**: p. 46.

112. Lefever, J.B., et al., *Cell phones and the measurement of child neglect: the validity of the parent-child activities interview.* Child Maltreatment, 2008. **13**(4): p. 320-33.

113. Leijdekkers, P., V. Gay, and E. Barin, *Trial Results of a Novel Cardiac Rhythm Management System Using Smart Phones and Wireless ECG Sensors.* Ambient Assistive Health and Wellness Management in the Heart of the City, Proceeding, 2009. **5597**: p. 32-39.

114. Lester, R.T., et al., *The HAART cell phone adherence trial (WelTel Kenya1): a randomized controlled trial protocol.* Trials [Electronic Resource], 2009. **10**: p. 87.

115. Maguire, R., et al., *A randomised controlled trial of a remote monitoring, mobile phone based, advanced symptom management system in patients with colorectal, lung and breast cancer receiving chemotherapy.* Ejc Supplements, 2007. **5**(4): p. 8069.

116. Main, D.S., et al., *Exploring patient reactions to pen-tablet computers: a report from CaReNet.* Annals of Family Medicine, 2004. **2**(5): p. 421-4.

117. Marceau, L.D., et al., *Electronic diaries as a tool to improve pain management: is there any evidence?* Pain Medicine, 2007. **8 Suppl 3**: p. S101-9.

118. Matthew, A.G., et al., *Serial personal digital assistant data capture of health-related quality of life: A randomized controlled trial in a prostate cancer clinic.* Health and Quality of Life Outcomes, 2007. **5**.

119. McBride, J.S., R.T. Anderson, and J.L. Bahnson, *Using a hand-held computer to collect data in an orthopedic outpatient clinic: a randomized trial of two survey methods.* Medical Care, 1999. **37**(7): p. 647-51.

120. McCann, L., et al., *Patients' perceptions and experiences of using a mobile phone-based advanced symptom management system (ASyMS) to monitor and manage chemotherapy related toxicity.* European Journal of Cancer Care, 2009. **18**(2): p. 156-64.

121. McClung, H.L., et al., *Monitoring energy intake: a hand-held personal digital assistant provides accuracy comparable to written records.* Journal of the American Dietetic Association, 2009. **109**(7): p. 1241-5.

122. McCormack, K., et al., *A Comparison of a Computer Game-Based Exercise System with Conventional Approaches of Exercise Therapy in Rheumatology Patients.* Rheumatology, 2009. **48**: p. I29-I29.

123. McDoniel, S.O., P. Wolskee, and J. Shen, *Treating obesity with a novel hand-held device, computer software program, and internet technology in primary care: The smart motivational trial.* Patient Education and Counseling Aug, 2009(Pagination): p. No Pagination Specified.

124. Michalowski, W., R. Slowinski, and S. Wilk, *MET system: A new approach to m-health in Emergency Triage.* Journal on Information Technology in Healthcare, 2004. **2**(4): p. 237-249.

125. Mignogna, J., M. Jackson, and T.R. Leffingwell, *Ru Drnkn 2nite?: A Randomized Trial of Cellular Text Messaging Prompts for Alcohol Harm-Reduction on Campus.* Annals of Behavioral Medicine, 2007. **33**: p. S219-S219.

126. Miskelly, F., *Electronic tracking of patients with dementia and wandering using mobile phone technology.* Age & Ageing, 2005. **34**(5): p. 497-9.

127. Morgan, P.B., et al. (2007) *Elicitation of subjective responses via sms (text) messaging in contact lens clinical trials*. American Academy of Optometry **Volume**,

128. Mosso, J.L., et al., *Virtual reality on mobile phone to reduce anxiety in outpatient surgery.* Annual Review of CyberTherapy and Telemedicine, 2008. **6**: p. 140-145.

129. Murray, B.J., J. Hutton, and A. Lloyd, *Next generation electronic patient diary applications in life cycle management and economic outcomes studies.* European Pharmaceutical Contractor., 2003.

130. Neville, R., et al., *Mobile phone text messaging can help young people manage asthma.* BMJ, 2002. **325**(7364): p. 600.

131. Newell, A., *A mobile phone text message and Trichomonas vaginalis.* Sex Transm Infect, 2001. **77**(3): p. 225.

132. Newman, M.G., et al., *Comparison of palmtop-computer-assisted brief cognitive-behavioral treatment to cognitive-behavioral treatment for panic disorder.* Journal of Consulting and Clinical Psychology, 1997. **65**(1): p. 178-183.

133. Nguyen, H.Q., et al., *Randomized controlled trial of an internet-based versus face-to-face dyspnea self-management program for patients with chronic obstructive pulmonary disease: pilot study.* Journal of Medical Internet Research, 2008. **10**(2): p. e9.

134. Nguyen, H.Q., et al. (2006) *Exercise and symptom monitoring with a mobile device*. AMIA ... Annual Symposium proceedings / AMIA Symposium. AMIA Symposium **Volume**, 1047

135. Nyholm, D., J. Kowalski, and S.-M. Aquilonius, *Wireless real-time electronic data capture for self-assessment of motor function and quality of life in Parkinson's disease.* Movement Disorders, 2004. **19**(4): p. 446-51.

136. Palen, L.-A., et al., *Rates of missing responses in personal digital assistant (PDA) versus paper assessments.* Evaluation Review, 2008. **32**(3): p. 257-72.

137. Park, M.-J., H.-S. Kim, and K.-S. Kim, *Cellular phone and Internet-based individual intervention on blood pressure and obesity in obese patients with hypertension.* International Journal of Medical Informatics, 2009. **78**(10): p. 704-10.

138. Patnaik, S., E. Brunskill, and W. Thies, *Evaluating the Accuracy of Data Collection on Mobile Phones: A Study of Forms*, in *International Conference on Information and Communication Technologies and Development*. 2009: Doha, Qatar.

139. Peng, W., *Design and evaluation of a computer game to promote a healthy diet for young adults.* Health Communication, 2009. **24**(2): p. 115-27.

140. Perkins, N.A., et al., *Performance of drug-drug interaction software for personal digital assistants.* Ann Pharmacother, 2006. **40**(5): p. 850-5.

141. Prestwich, A., M. Perugini, and R. Hurling, *A randomized controlled trial of implementation intentions and text message interventions to promote walking.* Psychology & Health, 2008. **23**: p. 216-216.

142. Pulley, K.R. and M. Flanders-Stepans (2002) *Smoking hygiene: an educational intervention to reduce respiratory symptoms in breastfeeding infants exposed to tobacco*. Journal of Perinatal Education **Volume**, 28-37

143. Quinn, C.C., et al., *Mobile diabetes intervention study: testing a personalized treatment/behavioral communication intervention for blood glucose control.* Contemporary Clinical Trials, 2009. **30**(4): p. 334-46.

144. Raisch, D.W., et al., *Collection of drug dispensing data in a multi-center clinical trial using hand-held computers.* Controlled Clinical Trials, 2003. **24**: p. P282.

145. Reynolds, P.A., et al., *Portable digital assistants (PDAs) in dentistry: part II--pilot study of PDA use in the dental clinic.* British Dental Journal, 2007. **202**(8): p. 477-83.

146. Rezaiyan, A., E. Mohammadi, and P.A. Fallah, *Effect of computer game intervention on the attention capacity of mentally retarded children.* International Journal of Nursing Practice, 2007. **13**(5): p. 284-8.

147. Richter, J.G., et al., *Self-assessments of patients via Tablet PC in routine patient care: comparison with standardised paper questionnaires.* Annals of the Rheumatic Diseases, 2008. **67**(12): p. 1739-41.

148. Ring, A.E., et al., *A randomized study of electronic diary versus paper and pencil collection of patient-reported outcomes in patients with non-small cell lung cancer.* The Patient: Patient Centered Outcomes Research, 2008. **1**(2): p. 106-113.

149. Rivellese, A.A., et al., *Evaluation of new computerized method for recording 7-day food intake in IDDM patients.* Diabetes Care, 1991. **14**(7): p. 602-4.

150. Rivera, M.L., et al., *Prospective, randomized evaluation of a personal digital assistant-based research tool in the emergency department.* BMC Medical Informatics & Decision Making, 2008. **8**: p. 3.

151. Rosenberger, E.L., et al., *Implementing a palm pilot intervention for primary care providers: lessons learned.* Contemporary Clinical Trials, 2009. **30**(4): p. 321-5.

152. Rowan, P.J., et al., *Evaluating reactivity to ecological momentary assessment during smoking cessation.* Experimental & Clinical Psychopharmacology, 2007. **15**(4): p. 382-9.

153. Ruder, K., *Test messaging for teens :-).* Diabetes Forecast, 2007. **60**(9): p. 28.

154. Ruland, C.M., *Handheld technology to improve patient care: evaluating a support system for preference-based care planning at the bedside.* J Am Med Inform Assoc, 2002. **9**(2): p. 192-201.

155. Ruland, C.M., et al., *Effects of a computerized system to support shared decision making in symptom management of cancer patients: preliminary results.* Journal of the American Medical Informatics Association, 2003. **10**(6): p. 573-9.

156. Russoniello, C.V., K. O'Brien, and J.M. Parks, *The effectiveness of casual video games in improving mood and decreasing stress.* Journal of CyberTherapy and Rehabilitation, 2009. **2**(1): p. 53-66.

157. Ryan, D., et al., *The CYMPLA trial. Mobile phone-based structured intervention to achieve asthma control in patients with uncontrolled persistent asthma: A pragmatic randomised controlled trial.* Primary Care Respiratory Journal, 2009. **18**(4): p. 343-345.

158. Ryuzaki, M., et al., *Weekly and seasonal variations of home blood pressure in hemodialyzed patients evaluated by telemedicine system using cellular phone.* Journal of Hypertension, 2008. **26**: p. S485-S485.

159. Saleh, K.J., et al., *Comparison of commonly used orthopaedic outcome measures using palm-top computers and paper surveys.* Journal of Orthopaedic Research, 2002. **20**(6): p. 1146-51.

160. Samore, M.H., et al., *Clinical decision support and appropriateness of antimicrobial prescribing: a randomized trial.* JAMA, 2005. **294**(18): p. 2305-14.

161. Sawa, T., et al., *Preoperative information management system using wireless PDAs.* AMIA .. 2003. **Annual Symposium Proceedings/AMIA Symposium.**: p. 995.

162. Schlickum, M.K., et al., *Systematic video game training in surgical novices improves performance in virtual reality endoscopic surgical simulators: A prospective randomized study.* World Journal of Surgery, 2009. **33**(11): p. 2360-2367.

163. Seebregts, C.J., et al., *Handheld computers for survey and trial data collection in resource-poor settings: development and evaluation of PDACT, a Palm Pilot interviewing system.* International Journal of Medical Informatics, 2009. **78**(11): p. 721-31.

164. Sellors, J.W., et al., *Comparison of deferral rates using a computerized versus written blood donor questionnaire: a randomized, cross-over study [ISRCTN84429599].* BMC Public Health, 2002. **2**: p. 14.

165. Sevick, M.A., et al., *Design, feasibility, and acceptability of an intervention using personal digital assistant-based self-monitoring in managing type 2 diabetes.* Contemporary Clinical Trials, 2008. **29**(3): p. 396-409.

166. Shea, H.E., C. Preston, and S. Hudson, *Electronic patient diaries in a clinical trial - The holistic approach.* Drug Information Journal, 2004. **38**(3): p. 225-238.

167. Shelby-James, T.M., et al., *Handheld computers for data entry: High tech has its problems too.* Trials, 2007. **8**(5).

168. Silvey, G.M., et al., *Direct comparison of a tablet computer and a personal digital assistant for point-of-care documentation in eye care.* AMIA Annu Symp Proc, 2005: p. 689-93.

169. Snooks, H., et al., *Support and Assessment for Fall Emergency Referrals (SAFER 1) trial protocol. Computerised on-scene decision support for emergency ambulance staff to assess and plan care for older people who have fallen: evaluation of costs and benefits using a pragmatic cluster randomised trial.* BMC Emergency Medicine, 2010. **10**: p. 2.

170. Stack Jr, B.C., et al., *Initial experience with personal digital assistant-based reflectance photoplethysmograph for free tissue transfer monitoring.* Annals of Plastic Surgery, 2003. **51**(2): p. 136-140.

171. Standen, P.J., et al., *An evaluation of the use of microswitch controlled computer games in improving choice reaction time for adults with intellectual disabilities.* Journal of Applied Research in Intellectual Disabilities, 2006. **19**(3): p. 239-239.

172. Staresinic, A., et al. (2002) *Incorporation of handheld computer technology into environmental research [abstract]*. American Journal of Respiratory and Critical Care Medicine **Volume**, A108

173. Sterling, L., G.A. Tait, and J.F. Edmonds, *Interpretation of digital radiographs by pediatric critical care physicians using Web-based bedside personal computers versus diagnostic workstations.* Pediatric Critical Care Medicine, 2003. **4**(1): p. 26-32.

174. Stern, Y.C. (2007) *Pilot Study Using a Video Game to Train Cognitive Control Processes in Healthy Older Adults*. <http://wwwclinicaltrialsgov/ct2/show/NCT00586638?term=NCT00586638&rank=1> **Volume**,

175. Stone, A.A., et al., *A comparison of coping assessed by ecological momentary assessment and retrospective recall.* Journal of Personality & Social Psychology, 1998. **74**(6): p. 1670-80.

176. Stratton, R.J., et al., *Comparison of the traditional paper visual analogue scale questionnaire with an Apple Newton electronic appetite rating system (EARS) in free living subjects feeding ad libitum.* Eur J Clin Nutr, 1998. **52**(10): p. 737-41.

177. Tegang, S.P., et al. (2009) *A comparison of paper-based questionnaires with PDA for behavioral surveys in Africa: Findings from a behavioral monitoring survey in Kenya*. **Volume**,

178. Trapl, E.S., et al., *Use of audio-enhanced personal digital assistants for school-based data collection.* Journal of Adolescent Health, 2005. **37**(4): p. 296-305.

179. Tseng, H.M., et al., *Computer anxiety: a comparison of pen-based personal digital assistants, conventional computer and paper assessment of mood and performance.* British Journal of Psychology, 1998. **89**(Pt 4): p. 599-610.

180. Turnin, M.C., et al., *Learning good eating habits playing computer games at school: A 2000 children evaluation.* Diabetes Research and Clinical Practice, 2000. **50**(Suppl. 1): p. S239.

181. Turnin, M.C., et al., *Learning good eating habits playing computer games at school: A 2000 children experimentation.* Diabetologia, 1998. **41**: p. 971.

182. Turnin, M.C., et al., *Evaluation of microcomputer nutritional teaching games in 1,876 children at school.* Diabetes & Metabolism, 2001. **27**(4 Pt 1): p. 459-64.

183. van Griensven, F., et al., *Palmtop-assisted self-interviewing for the collection of sensitive behavioral data: randomized trial with drug use urine testing.* American Journal of Epidemiology, 2006. **163**(3): p. 271-8.

184. VanDenKerkhof, E.G., et al., *Using a personal digital assistant enhances gathering of patient data on an acute pain management service: A pilot study.* Canadian Journal of Anesthesia, 2003. **50**(4): p. 368-375.

185. Vasterling, J., et al., *Cognitive distraction and relaxation training for the control of side effects due to cancer chemotherapy.* Journal of Behavioral Medicine, 1993. **16**(1): p. 65-80.

186. Vidrine, D.J., R.C. Arduino, and E.R. Gritz, *Impact of a cell phone intervention on mediating mechanisms of smoking cessation in individuals living with HIV/AIDS.* Nicotine & Tobacco Research, 2006. **8 Suppl 1**: p. S103-8.

187. Walters, D.L., et al., *A mobile phone-based care model for outpatient cardiac rehabilitation: the care assessment platform (CAP).* BMC Cardiovascular Disorders, 2010. **10**: p. 5.

188. Wang, D.H., et al., *Validity and reliability of a dietary assessment method: the application of a digital camera with a mobile phone card attachment.* J Nutr Sci Vitaminol (Tokyo), 2002. **48**(6): p. 498-504.

189. Wetter, D. and P.M. Cinciripini (2006) *Palmtop computer-delivered treatments for smoking cessation (SYM4C)*. Society for Research on Nicotine and Tobacco 12th Annual Meeting February 15-18, Orlando, Florida **Volume**, 6

190. Woods, C.A., et al. (2006) *The assessment of contact lens symptomology using wireless handheld communication devices*. American Academy of Optometry **Volume**,

191. Wright, A., et al., *Effectiveness of health maintenance reminders provided directly to patients.* AMIA ... Annual Symposium proceedings / AMIA Symposium. AMIA Symposium., 2008.

192. Wright, P., et al. (2001) *Enhancing an appointment diary on a pocket computer for use by people after brain injury*. International Journal of Rehabilitation Research **Volume**,

193. Wright, P., et al., *Comparison of pocket-computer memory aids for people with brain injury.* Brain Injury, 2001. **15**(9): p. 787-800.

194. Yen, P.-Y. and P. Gorman, *Usability testing of digital pen and paper system in nursing documentation.* AMIA .. 2005. **Annual Symposium Proceedings/AMIA Symposium.**: p. 844-8.

195. Yoon, K.H. and H.S. Kim (2008) *A short message service by cellular phone in type 2 diabetic patients for 12 months*. Diabetes research and clinical practice **Volume**, 256-61

196. Young, P.M., et al., *An evaluation of the use of hand-held computers for bedside nursing care.* International Journal of Medical Informatics, 2001. **62**(2-3): p. 189-93.

197. Zhao, X., et al., *Integration of Information Technology, Wireless Networks, and Personal Digital Assistants for Triage and Casualty.* Telemedicine and e Health, 2006. **12**(4): p. 466-474.

198. Zwarenstein, M.F., et al., *A cluster randomized trial evaluating electronic prescribing in an ambulatory care setting.* Trials, 2007. **8**(28).
