# Supplementary material for: The Effectiveness of Mobile-Health Technology-Based Health Behaviour Change or Disease Management Interventions for Health Care Consumers: A Systematic Review
Source: PLoS Med. 2013 Jan 15;10(1):e1001362. doi: 10.1371/journal.pmed.1001362 (PMC3548655; doi:10.1371/journal.pmed.1001362)
Supplement: Text S3 — MEDLINE (Ovid) search strategy. (DOC) [file pmed.1001362.s004.doc]

**Text S3: MEDLINE (Ovid) search strategy**

1. computers, handheld/

2. (computer$ adj4 handheld).ab,ti.

3. (computer$ adj4 hand-held).ab,ti.

4. (computer$ adj4 palm$).ab,ti.

5. (computer$ adj4 pocket).ab,ti.

6. (computer$ adj4 mobile).ab,ti.

7. (Pocket-PC$ or PocketPC$).ab,ti.

8. (Pocket adj computer$).ab,ti.

9. (Palm adj4 PDA$).ab,ti.

10. (PDA$ adj5 computer$).ab,ti.

11. (Personal adj digital adj assistant$).ab,ti.

12. (PDA$ adj5 phone$).ab,ti.

13. (PDA$ adj5 telephone$).ab,ti.

14. (Tablet adj computer$).ab,ti.

15. (Tablet adj PC$).ab,ti.

16. (Palm-Pilot$ or Palmpilot$).ab,ti.

17. (Palm adj (Pre or Treo or Centro)).ab,ti.

18. (smartbook$ or smart-book$).ab,ti.

19. ((ultra-mobile or ultramobile) and (PC$ or personal computer$)).ab,ti.

20. ((ultra-portable or ultraportable) and (PC$ or personal computer$)).ab,ti.

21. (enterprise adj digitial adj assistant).ab,ti.

22. (EDA$ adj4 computer$).ab,ti.

23. cellular phone/

24. (cellular adj3 phone$).ab,ti.

25. (mobile adj3 phone$).ab,ti.

26. (mobile adj3 telephone$).ab,ti.

27. (cellular adj3 telephone$).ab,ti.

28. (cell adj3 phone$).ab,ti.

29. (cell adj3 telephone$).ab,ti.

30. (smartphone$ or smart-phone$).ab,ti.

31. (blackberr$ or black-berr$).ab,ti.

32. (google adj3 phone$).ab,ti.

33. (nexus adj one adj3 phone$).ab,ti.

34. (application adj software).ab,ti.

35. (MMS or multimedia messaging service).ab,ti.

36. (SMS or short messaging service).ab,ti.

37. (text$ adj message$).ab,ti.

38. MP3 player/

39. MP3 player$.ab,ti.

40. MP4 player$.ab,ti.

41. (MP3 or MP4).ab,ti.

42. (portable adj2 media adj2 player$).ab,ti.

43. (iphone$ or i-phone$).ab,ti.

44. (ipod$ or i-pod$).ab,ti.

45. (podcast$ or pod-cast$).ab,ti.

46. Medical informatics/ or medical informatics applications/

47. audiovisual aids/

48. Multimedia/

49. Public health informatics/

50. User-computer interface/

51. Interactive tutorial/

52. ((mobile adj health) not van$ not unit$).ab,ti.

53. (mhealth or m-health).ab,ti.

54. (elearning or e-learning).ab,ti.

55. Electronic mail/

56. (electronic adj3 mail$).ab,ti.

57. (electronic adj3 messag$).ab,ti.

58. (email$ or e-mail$).ab,ti.

59. Hypermedia/

60. Video games/

61. (computer adj2 gam$).ab,ti.

62. (video adj2 gam$).ab,ti.

63. (electronic adj2 gam$).ab,ti.

64. (playstation adj1 portable).ab,ti.

65. (Sony adj1 PSP).ab,ti.

66. (gameboy adj (advance or micro)).ab,ti.

67. Nintendo DS$.ab,ti.

68. Gamepark.ab,ti.

69. Gizmando.ab,ti.

70. (Tapwave adj zodiac).ab,ti.

71. Video recording/

72. (video or videos).ab,ti.

73. computer graphics/

74. Internet/

75. internet.ab,ti.

76. ("world wide web" or world-wide-web or "world-wide web" or "worldwide web" or website$ or web-site$).ab,ti.

77. (WAP or "wireless application protocol").ab,ti.

78. online.ab,ti.

79. (on?-line not one line).ti,ab.

80. web?based.ab,ti.

81. web-based.ab,ti.

82. bluetooth.ab,ti.

83. (web adj3 technolog$).ab,ti.

84. (chat?room$ or chat-room).ab,ti.

85. (blog$ or web-log$ or weblog$).ab,ti.

86. Blogging/

87. (bulletin adj board$).ab,ti.

88. (message adj board$).ab,ti.

89. (interactive adj5 health adj5 communicat$).ab,ti.

90. (interactive adj3 televis$).ab,ti.

91. (interactive adj3 TV).ab,ti.

92. (interactive adj4 technolog$).ab,ti.

93. (interactive adj7 multimedia).ab,ti.

94. (interactive adj3 software).ab,ti.

95. (e-health$ or ehealth$).ab,ti.

96. (electronic adj health).ab,ti.

97. (consumer adj1 health adj1 informatic$).ab,ti.

98. (virtual adj reality).ab,ti.

99. (virtual adj learning).ab,ti.

100. (surf adj4 web$).ab,ti.

101. (surfing adj4 web$).ab,ti.
